# Differential Regulation of circRNA, miRNA, and piRNA during Early Osteogenic and Chondrogenic Differentiation of Human Mesenchymal Stromal Cells

**DOI:** 10.3390/cells9020398

**Published:** 2020-02-09

**Authors:** Elena Della Bella, Ursula Menzel, Valentina Basoli, Céline Tourbier, Mauro Alini, Martin J. Stoddart

**Affiliations:** 1 AO Research Institute Davos, 7270 Davos Platz, Switzerland; elena.dellabella@aofoundation.org (E.D.B.); ursula.menzel@aofoundation.org (U.M.); Valentina.basoli@aofoundation.org (V.B.); celine.tourbier@aofoundation.org (C.T.); mauro.alini@aofoundation.org (M.A.); 2 Department of Cranio-Maxiofacial Surgery, Medical Center-Albert-Ludwigs-University of Freiburg, Faculty of Medicine, Albert-Ludwigs-University of Freiburg, 79085 Freiburg, Germany; 3 Department of Orthopedics and Trauma Surgery, Medical Center - Albert-Ludwigs-University of Freiburg, Faculty of Medicine, Albert-Ludwigs-University of Freiburg, 79106 Freiburg, Germany

**Keywords:** human mesenchymal stem cells, RNA sequencing, circular RNA, miRNA, piwi-interacting RNA, dexamethasone, differentiation, transcriptome, non-coding RNA

## Abstract

The goal of the present study is to identify the differential expression of circular RNA (circRNA), miRNA, and piwi-interacting RNA (piRNA) after lineage commitment towards osteo- and chondrogenesis of human bone marrow mesenchymal stromal cells (hMSCs). The cells were maintained for 7 days in either osteogenic or chondrogenic medium. RNA sequencing was performed to assess the expression of miRNA and piRNA, while RNA hybridization arrays were used to identify which circRNA were differentially expressed. qPCR validation of a selection of targets for both osteogenic and chondrogenic differentiation was carried out. The differential expression of several circRNA, miRNA, and piRNA was identified and validated. The expression of total and circular isoforms of *FKBP5* was upregulated both in osteo- and chondrogenesis and it was influenced by the presence of dexamethasone. *ZEB1*, *FADS2*, and *SMYD3* were also identified as regulated in differentiation and/or by dexamethasone. In conclusion, we have identified a set of different non-coding RNAs that are differentially regulated in early osteogenic and chondrogenic differentiation, paving the way for further investigation to understand how dexamethasone controls the expression of those genes and what their function is in MSC differentiation.

## 1. Introduction

Bone marrow-derived mesenchymal stromal cells (MSCs) potentially represent a good source of cells for bone and cartilage tissue engineering. MSCs are widely studied and used in several clinical trials, with >80 active trials (clinicaltrials.gov, September 2019) and at least 20 for the treatment of musculoskeletal diseases. Despite all the effort, there is still little evidence that the use of MSCs is effective in musculoskeletal tissue repair. The reasons are manifold, one being the lack of control of a cell’s final differentiation. For example, the formation of mechanically inferior fibrous cartilage, instead of hyaline, is one the most common outcomes for articular cartilage tissue engineering [1]. Moreover, the extensive ex vivo manipulation required to obtain large quantities of cells may alter their phenotype and response [2]. Thus, there is a strong need for a better understanding of the underlying molecular mechanisms in order to achieve an efficient differentiation. Besides the genes coding for lineage-specific proteins, such as transcription factors, surface receptors, or matrix proteins, cellular processes comprise a variety of different non-coding RNAs (ncRNA) which have regulatory roles and actively participate in lineage specification. The study of ncRNA is therefore of utmost importance to understand the molecular mechanisms of differentiation and how to tune their regulation to be able to build and replace tissues.

The most studied type of ncRNA are microRNAs (miRNA), and their role in stem cell maintenance and differentiation has been investigated [3,4,5]. A related class of small ncRNA is represented by piwi-interacting RNA (piRNA). piRNA associates with the Piwi subfamily of Argonaute proteins, protecting genome integrity of germline cells by silencing transposable elements through the formation of RNA-induced silencing complexes with Piwi proteins, also known as piRISC [6,7]. Accordingly, their expression has been identified in testis [8], ovary [9], and epididymis [10]. Nonetheless, there is a growing body of evidence that piRNAs also possess important regulatory functions in somatic and cancer cells [7,11,12,13,14,15,16,17,18].

Circular RNA (circRNA) represents a relatively newly recognized class of long ncRNA. Even though their existence has been long acknowledged, circular forms of RNA were thought to merely represent splicing errors with no biological function. Through the advancement of next generation sequencing, which has allowed the rise in RNA sequencing from diverse samples, circRNAs are now recognized to have distinct biogenesis and to regulate gene expression and biological processes through different mechanisms [19,20], with some miRNA-sponging circRNAs identified [21,22,23,24,25,26]. Moreover, since circRNAs derive from precursor mRNA, they are believed to influence the transcription and/or translation of the linear, protein-coding form [20].

In any case, the role of circRNA in different biological processes is becoming more and more evident. Up to now, a few publications have reported the expression and possible functions of circRNA in MSCs from different organisms, tissue sources, and experimental settings [27,28,29,30,31,32]. circRNA were differentially expressed in osteogenic differentiation of rat bone marrow MSCs engineered with estrogen receptor β-targeting shRNA [33]. In human cells, the involvement of circRNAs in osteogenic differentiation of periodontal ligament stem cells [34,35,36] or maxillary sinus membrane stem cells [37] was reported. One study focused on the osteogenic differentiation of bone marrow stem cells [38]. A map of circRNA expression in clinically relevant tissues, including MSCs and differentiated cells, was also suggested [39] but, until now, there is a paucity of data regarding differential circRNA expression during osteogenic and chondrogenic differentiation of human bone marrow MSCs.

The aim of the present study is to identify differential expression of circRNA, miRNA, and piRNA after lineage commitment towards osteo- and chondrogenesis. Based on previous work highlighting the importance of early events during differentiation, we investigated after day 7 of initiation of differentiation induction. Thus, human MSCs were maintained for 7 days in either osteogenic or chondrogenic medium with relative controls, and RNA sequencing or RNA hybridization arrays were performed. We then identified and validated several differentially regulated circRNA, miRNA, and piRNA, and revealed for some genes the influence of dexamethasone.

## 2. Materials and Methods

### 2.1. Cell Isolation and Culture

MSCs were isolated from human bone marrow, as previously described [40]. The samples were obtained with full ethical approval (Bern Req-2016-00141). Cells from a total of 16 donors were used (11 M/5 F; age mean 60 years; age range 33–80). Cells were cultured maintaining an initial cell density of 3 × 10^3^ cells/cm^2^ and grown until passage 2 (p2) in Minimum Essential Medium Eagle-alpha modification (α-MEM, Gibco, Thermo Fisher, Zürich, Switzerland) with the addition of 10% MSC-qualified FBS (Pan-Biotech, Aidenbach, Germany), 100 U/mL penicillin, 100 μg/mL streptomycin (Gibco) and 5 ng/mL basic fibroblast growth factor (bFGF, Fitzgerald Industries International, Acton, MA, USA). Cultures were kept in a 37 °C/5% CO_2_ humidified atmosphere and the medium was refreshed every second day.

For initial RNA sequencing and hybridization arrays, MSCs from three donors were used. Cells were profiled for standard MSC markers with flow cytometry, as previously described [41]. For further validation, MSCs from 6 additional donors for osteogenic differentiation and chondrogenic differentiation were isolated and differentiated following the same procedure (final *n* = 9). At p3, cells were induced to either osteo- or chondrogenic differentiation or cultured under control conditions.

For osteogenic differentiation, cells were seeded at a density of 1.5 × 10^4^ cells/cm^2^ in 6-well plates. After overnight cell attachment, the medium was changed: control (CRL monolayer) samples were cultured in Dulbecco’s Modified Eagle Medium (DMEM) 1 g/L glucose (Gibco), 10% heat-inactivated FBS (Gibco), 100 U/mL penicillin and 100 μg/mL streptomycin (Gibco). Osteogenic differentiation was induced by the addition of 50 µg/mL ascorbic acid 2-phosphate, 5 mM β-glycerol phosphate, and 10 nM dexamethasone (all from Sigma-Aldrich) to the control medium. Samples were prepared in triplicate for each donor and condition.

For chondrogenic differentiation, cells were seeded into pellet culture, using 2 × 10^5^ cells/pellet. Cells were directly seeded into either control (CRL pellet) or chondrogenic medium. Control medium was composed of DMEM 4.5 g/L glucose, 100 U/mL penicillin, 100 μg/mL streptomycin, 1% Corning ITS+ Premix Universal Culture Supplement, and 1% non-essential amino acids (NEAA). Chondrogenic medium was prepared from control medium with the addition of 50 µg/mL ascorbic acid 2-phosphate, 100 nM dexamethasone, and 10 ng/mL TGF-β1 (Fitzgerald Industries). The medium was refreshed every second day to keep stable levels of differentiation factors in the media and samples were collected at day 7 for RNA isolation.

Moreover, cultures were maintained up to day 21 for assessing final differentiation outcomes (Alizarin Red staining for osteogenic differentiation and Safranin-O/Fast Green staining on cryosections for chondrogenic differentiation). The RNA samples derived from those three donors were not used for sequencing but for validation purposes only. A summary of donor cohorts used for all experiments is reported in Table S1.

### 2.2. RNA Isolation

Total RNA was isolated from day 7 control (monolayer), day 7 control (pellet), day 7 osteogenic differentiation (OSTEO), and day 7 chondrogenic differentiation (CHONDRO). For control (monolayer) and OSTEO samples, RNA was isolated from 1 well in triplicate for each donor. For control (pellet) and CHONDRO samples, 4 pellets were pooled in one sample and three samples were collected for each condition. Total RNA was isolated using standard TRIreagent (Molecular Research Center Inc., Cincinnati, OH, USA) extraction with 1-Bromo-3-chloropropane (Sigma-Aldrich, St. Louis, MO, USA). After phase separation, RNA was precipitated from the aqueous phase with the addition of 2-propanol (Sigma-Aldrich) with an overnight incubation at −20 °C to improve the recovery of small RNA species. Total RNA concentration was measured with NanoDrop 1000 (Thermo Fisher) and purity was assessed by evaluation of the A260/280 and A260/230 ratios.

### 2.3. RNA Sequencing and Hybridization

Two replicates for each group were selected for sequencing (*n* = 24 samples). Two µg of total RNA for each sample was collected and dried in RNAstable^TM^ tubes (Biomatrica, San Diego, CA, USA) following the manufacturer’s instruction. As previously described [42], samples were sent to ArrayStar (Rockville, MD, USA) for library generation and sequencing and RNA microarray hybridization.

Libraries for RNAseq were denatured as single-stranded DNA molecules, captured on Illumina flow cells, amplified in situ as clusters, and sequenced for 51 cycles on an Illumina NextSeq500 system per the manufacturer’s instructions.

For miRNA, the Solexa CHASTITY quantity filtered reads were harvested after sequencing as clean reads. The adaptor sequences were trimmed with cutadapt and the adaptor-trimmed reads (≥ 15 nt) were left. miRDeep2 software was used to quantify known miRNA and predict the novel miRNAs. The CPM value for miRNA and the differentially expressed miRNA were calculated and filtered with R package edgeR. Fold change (cutoff 1.5), *p*-value (0.05), and CPM (1 mean in one group) were used for filtering differentially expressed miRNAs. Hierarchical clustering was performed. miRNA target prediction was performed by targetscan (www.targetscan.org) and miRDB (www.mirdb.org), then the GO and KEGG pathway analyses were performed based on the top 10 differentially expressed miRNAs.

For piRNA, the quality of sequencing was examined by FastQC software (https://www.bioinformatics.babraham.ac.uk/projects/fastqc/) and the trimmed reads (that passed Illumina quality filter, trimmed 3’-adaptor bases by cutadapt) were aligned to piRBase (www.pirbase.org), a manually curated database for human piRNA [43,44], using NovoAlign software (v2.07.11, Novocraft Technologies Sdn Bhd, Selangor, Malaysia). The maximum mismatch of ≤2 reads were kept. The expression profiling and differential expression of piRNAs were calculated based on normalized TPM. Hierarchical clustering, scatter plots, and classification analysis were performed with the differentially expressed piRNAs in R or Perl environment for statistical computing and graphics. piRNA annotation and references were gathered from piRBase.

For circRNAs, samples were digested with RNase R (Epicentre Inc., Madison, WI, USA) for enrichment in circRNA content, which was then amplified and transcribed into fluorescent cRNA utilizing a random priming method (Arraystar Super RNA Labeling Kit; Arraystar). The labeled cRNAs were hybridized onto the Arraystar Human circRNA Array V2 (8 × 15K, Arraystar) and the arrays were scanned with an Agilent Scanner G2505C. Image analysis was performed with Agilent Feature Extraction software (version 11.0.1.1, Agilent Technologies Inc. Santa Clara, CA, USA). Quantile normalization and subsequent data processing were performed using the R software limma package [45]. Differentially expressed circRNAs with statistical significance between two groups were identified through Volcano Plot filtering. Hierarchical clustering was performed to show circRNA expression patterns among samples.

Quality controls have been performed by the company at key steps to ensure data quality. RNA sample quality control was carried out using denaturing agarose gel electrophoresis to check for RNA integrity and gDNA contamination. All total RNA samples had intense, sharp, and well-defined 28S and 18S ribosomal RNA bands, indicating minimal RNA degradation. DNA contamination as a high molecular weight smear or band was absent. For RNAseq, the quality of the sequencing libraries was determined for size distribution and library DNA concentration by an Agilent 2100 Bioanalyzer using the Agilent DNA 1000 chip kit (Agilent Technologies Inc., part #5067-1504). The sequencing read quality of each sample was analyzed by a Q Score plot, with a Q30 above 90% for all the samples. For arrays, the cRNA yield for each sample was >1.65 μg and the labeling specific activity was >9.0 pmol Cy3 dye per μg cRNA, which were all above the technical QC specifications. The microarray raw scan data all passed spot finding, spatial distribution of outliers, signal statistics, and control stats criteria.

### 2.4. RT-qPCR for Validation of Gene Expression from Total RNA and Linear RNA-Depleted Samples

For total gene expression, cDNA was synthesized using TaqMan Reverse Transcription reagents (Applied Biosystems, Foster City, CA, USA) from 500 ng of total RNA, following the manufacturer’s instructions. The expression of protein-coding linear transcripts, corresponding to the circRNA with differential expression in our dataset, was assessed by real-time PCR. Also, differentiation markers were analyzed, such as *RUNX2*/*SOX9* and *ALPL* or *SOX9*, *COL2A1*, and *ACAN* in osteogenic and chondrogenic differentiation, respectively. Amplification of target genes was achieved using TaqMan Gene Expression Master Mix (Applied Biosystems) in a QuantStudio 6 Flex real-time PCR system (Applied Biosystems) employing the following protocol: 2 min at 50 °C; 10 min at 95 °C; 40 cycles of 15 sec at 95 °C, 1 min at 60 °C. The details about the assays used are collected in Table S2. The genes were tested using the TaqMan gene expression assay (Thermo Fisher), with the exception of *RPLP0* (reference gene), *ACAN*, *COL1A1*, *COL2A1*, and *RUNX2*, whose sequences were synthesized by Microsynth AG (Balgach, Switzerland). Results of gene expression were expressed as 2^−ΔCt^, with *RPLP0* used as a reference gene.

To evaluate gene expression in linear RNA-depleted samples, 1 µg of total RNA was treated with RNase R for 2 h at 37 °C, immediately followed by the cDNA synthesis described above. The gene expression levels of *RPLP0*, *FKBP5*, *ZEB1*, *FADS2*, and *SMYD3* were assessed using the same protocols described above.

### 2.5. Validation of Dexamethasone-Dependent Targets

In order to understand if the expression of common differentially expressed circRNA in osteo- and chondrogenesis could be ascribable to the use of dexamethasone only, independently from the differentiation pathway undertaken, we prepared cell cultures of four additional donors. MSCs were isolated and expanded as described above. After expansion, cells at p3 were seeded into monolayer or pellet culture, as described earlier for controls for osteo- and chondrogenic differentiation. The cells were either maintained in control media or with the addition of 10 nM (in case of monolayer culture) or 100 nM dexamethasone (for pellet culture) in order to reflect the different dosage used previously for the two differentiation protocols. Moreover, additional groups containing 10 nM or 100 nM of the selective nonsteroidal glucocorticoid receptor agonist (+)-ZK216348 (Axon Medchem, Groningen, the Netherlands) were also included to identify whether the mechanism of action observed for dexamethasone could be attributed to transactivation or transrepression pathway. Cells were maintained in culture for 7 days, and then samples were collected for RNA isolation. Gene expression analysis of *FKBP5*, *FADS2*, *ZEB1*, and *SMYD3* was performed as described above.

### 2.6. Validation of miRNA and piRNA Expression

The expression of a selection of miRNA and piRNA was validated in the original set of samples via qPCR analysis. cDNA synthesis was performed with a miRCURY LNA RT Kit (Qiagen, Hilden, Germany), using 10 ng of starting RNA according to the manufacturer’s instructions. After reverse transcription, qPCR was performed using assays specific for the following miRNA or piRNA: hsa-let-7a-5p, hsa-miR-21-5p, hsa-let-7i-5p, hsa-miR-5690, hsa-miR-125b-5p, hsa-miR-1246, piR-hsa-20757 (DQ590548), piR-hsa-23209 (DQ592931), piR-hsa-23210 (DQ592932), piR-hsa-2107 (DQ571813), and piR-hsa-24672 (DQ594453) (all from Qiagen). The cycling conditions were set as follows: 2 min at 95 °C; 40 cycles of 10 sec at 95 °C, and 60 sec at 56 °C. The protocol was concluded by a melt-curve analysis to check for amplicon specificity. Then, hsa-let-7a-5p was used for the normalization of miRNA, which was differentially expressed in osteogenic differentiation, while hsa-miR-21-5p was used for normalization of miRNA in chondrogenic differentiation. In our set of samples, those two miRNAs resulted in the most stable in expression among a panel that also included hsa-miR-199a-3p, hsa-let-7f-5p, hsa-miR-7e-5p, and hsa-miR-100-5p. For piRNA, it was not possible to identify any suitable endogenous control. For this reason, the results were expressed as 2^−Ct^ and values compared between differentiation vs. undifferentiated controls. This provides only an initial indicative data to further validate those targets and to undertake a path leading to the identification of “housekeeping” piRNA and a proper qPCR analysis of piRNA.

### 2.7. Statistical Analysis

GraphPad Prism v.8 (GraphPad Software, San Diego, CA, USA) was used for statistical analysis. A two-tailed ratio paired Student’s *t*-test was used to compare expression levels between day 7 differentiation vs. its relative control. For the validation of dexamethasone-dependent targets in monolayer or pellet cultures, the results were analyzed with a two-way ANOVA and Tukey’s multiple comparison test to assess the effect of culture type, treatment, and their interaction on gene expression.

## 3. Results

### 3.1. MSC Immunophenotype and Osteo/Chondrogenic Differentiation Potential

As expected, MSCs showed the classical surface CD marker expression, with positivity for CD44, CD73, CD90, and CD105, while being negative for CD11b, CD19, CD34, and CD45. Results are summarized in Figure S1A,B.

Figure S1 also shows the differentiation potential of MSCs towards the osteogenic and chondrogenic lineages, with Alizarin Red and Safranin-O staining, respectively. Moreover, analysis of day 7 gene expression shows the regulation of the *RUNX2*/*SOX9* ratio [46] and *ALPL* expression in early osteogenesis, and upregulation of *SOX9*, *COL2A1*, and *ACAN* in early chondrogenic differentiation.

### 3.2. Differential Expression of circRNA in Early Osteo- and Chondrogenesis

Bioinformatic analysis of hybridization arrays revealed 21 upregulated circRNAs and 21 downregulated in day 7 osteogenic differentiation compared to day 7 control (monolayer), while 130 were upregulated and 97 downregulated during chondrogenic differentiation (Figure S2). Table 1 and Table 2 show a selection of the differentially expressed circRNA in osteogenic and in chondrogenic differentiation, respectively. The full list can be found in Tables S3 and S4. The data are available in NCBI’s Gene Expression Omnibus [47] and are accessible through GEO series accession number GSE135588 (https://www.ncbi.nlm.nih.gov/geo/query/acc.cgi?acc=GSE135588), subseries GSE135883 (https://www.ncbi.nlm.nih.gov/geo/query/acc.cgi?acc=GSE135883).

As shown in Table 1 and Table S3, the top seven upregulated circRNA in osteogenic differentiation are derived from the same two genes: *FKBP5* (4 circRNA) and *FADS2* (3 circRNA). Overall, the 21 upregulated circRNAs are originated from 14 protein-coding genes and 1 long non-coding RNA. The *ZEB1* gene also gives origin to 2 circRNA. Downregulated circRNAs in osteogenesis likewise comprise multiple circRNA derived from a single gene, such as *SMYD3* (*n* = 4) or *PDK1* (*n* = 3), with a total of 12 genes and 1 intergenic sequence originating the 21 downregulated circles.

Similarly, in chondrogenic differentiation (Table 2 and Table S4) many circRNAs share the same precursor gene, such as *FKBP5* (*n* = 3), *FADS2* (*n* = 3), *ZEB1* (*n* = 5), *PH4B* (*n* = 2), or *RUNX2* (*n* = 2) within upregulated circRNAs, or *FGFR1* (*n* = 2) and *SMYD3* (*n* = 3) among those downregulated. Interestingly, *ADAMTS6* is represented with two circRNA, one upregulated and one downregulated in chondrogenic differentiation.

**Figure 1:**
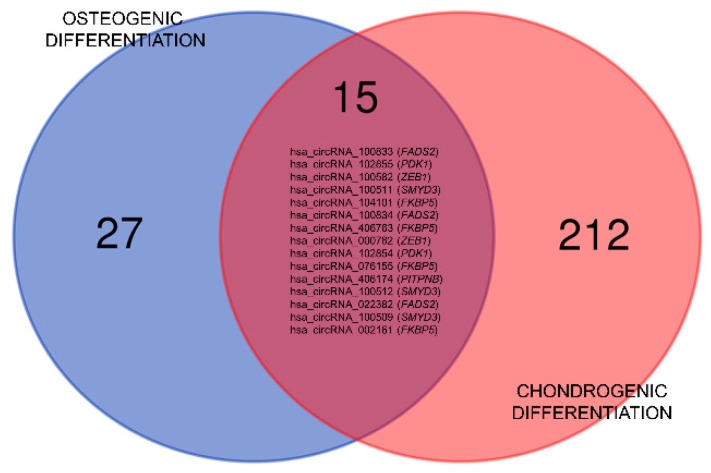
Venn diagram summarizing circRNA differential regulation in osteogenic and in chondrogenic differentiation. The chart was generated with the help of the following online tool: http://bioinformatics.psb.ugent.be/webtools/Venn/. The number of unique elements was 254 (42 for osteogenic and 227 for chondrogenic differentiation).

Of note, it was observed that five genes originate circRNA that are differentially expressed in both osteo- and chondrogenesis. In particular, *FKBP5*, *FADS2*, and *ZEB1* were upregulated and *SMYD3* was downregulated in both, while *PDK1* was upregulated in osteo- and downregulated in chondrogenic differentiation. The Venn chart in Figure 1 summarizes the common circRNAs in early osteo- and chondrogenesis.

For validation purposes, we first ranked differentially expressed circRNA based on fold change; then, we selected a panel of species to be validated based mainly on fold change and gene derivation of the circRNA. FDR values were also taken into account, although it was not used as an absolute discriminating factor between including a gene into the validation group or not.

### 3.3. Validation of Gene Expression from Total RNA and Linear RNA-Depleted Samples

Since circRNAs derive from precursor mRNA and the expression of linear and circular forms is co-regulated [20], we analyzed total gene expression for a selection of circRNAs.

**Figure 2:**
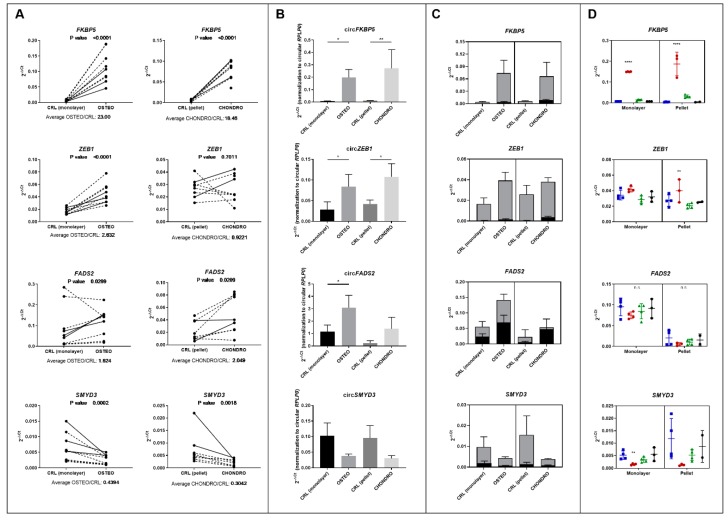
(**A**) Validation of total gene expression levels (*n* = 9) corresponding to differentially expressed circular RNAs in both osteogenic (left) and chondrogenic differentiation (right). Full lines represent results from the donors used for microarray (*n* = 3), while dashed lines correspond to additional donors tested (*n* = 6 for osteogenic and *n* = 6 for chondrogenic differentiation). For each gene of interest (GOI), the results are shown as 2^−ΔCt^, where ΔCt is calculated as the difference between GOI and reference gene (*RPLP0*) Ct values. The *p*-value and the average fold change between day 7 differentiation and day 7 controls are reported for each gene. (**B**) The expression of the same genes was tested after RNase R treatment (*n* = 3 donors) to validate the differential expression of the circular RNA. The expression of circular forms of genes was normalized to *RPLP0* analyzed in RNase R treated samples (circular *RPLP0*). (**C**) Proportion of circular transcripts on overall gene expression. The grey bar indicates the total amount of transcript for each gene, the black bar shows the amount of circular RNA. For this purpose, both circular and total transcripts were normalized to total *RPLP0* (*n* = 3 donors). (**D**) Effect of dexamethasone on the expression of total *FKBP5*, *ZEB1*, *FADS2*, and *SMYD3* in monolayer or pellet cultures. Blue bars and symbols: negative control (no treatment); red: dexamethasone treatment (10 nM in monolayer, 100 nM in pellet culture); green: (+)-ZK216348 treatment (10 nM in monolayer, 100 nM in pellet culture); black: vehicle control (DMSO). *n* = 4 donors. *: *p* < 0.05; **: *p* < 0.01; ****: *p* < 0.0001.

The results shown in Figure 2 focus on the analysis of gene expression for *FKBP5*, *FADS2*, *ZEB1*, and *SMYD3*, corresponding to the differentially expressed circRNAs in both osteo-and chondrogenic differentiation. Total gene expression is shown in Figure 2A, confirming their differential regulation in both differentiation cultures, except for *ZEB1* in chondrogenic differentiation. The analysis of the same genes in RNaseR treated-derived samples allowed us to evaluate circRNA expression (Figure 2B), with circular *FKBP5* and *ZEB1* being upregulated in both osteogenic and chondrogenic differentiation, while circular *FADS2* was significantly increased in osteogenesis only. Figure 2C shows the proportion of circular isoforms on total gene expression. In the case of *FADS2*, a high percentage of total gene expression is represented by the circular isoforms, especially in chondrogenic differentiation where they account for around 90% of the total transcript.

Moreover, in order to understand if total *FKBP5*, *FADS2*, *ZEB1*, and *SMYD3* expression was influenced by dexamethasone, we tested the action of dexamethasone only on either monolayer or pellet control culture, without the addition of any other differentiation agent. In addition, a group treated with the same concentrations of (+)-ZK216348 was included to distinguish between the transactivation or transrepression pathway of the glucocorticoid signaling pathway.

As depicted in Figure 2D, the results showed that *FKBP5* was affected only by the treatment type, with a marked upregulation observed for dexamethasone-treated cells both in monolayer and pellet cultures (fold change of 21.4 and 46.75, respectively, in comparison to the negative control), while no effect of (+)-ZK216348 was observed. A trend towards an upregulation of *ZEB1* and a downregulation of *SMYD3* was also observed, although it reached statistical significance only in pellet culture and in monolayer, respectively (*p* < 0.01). On the contrary, no influence of both dexamethasone or (+)-ZK216348 has been observed on *FADS2* expression, with only the culture type significantly affecting the levels of gene expression.

### 3.4. Validation of Total Gene Expression for Specific Osteo- or Chondrogenesis Targets

**Figure 3:**
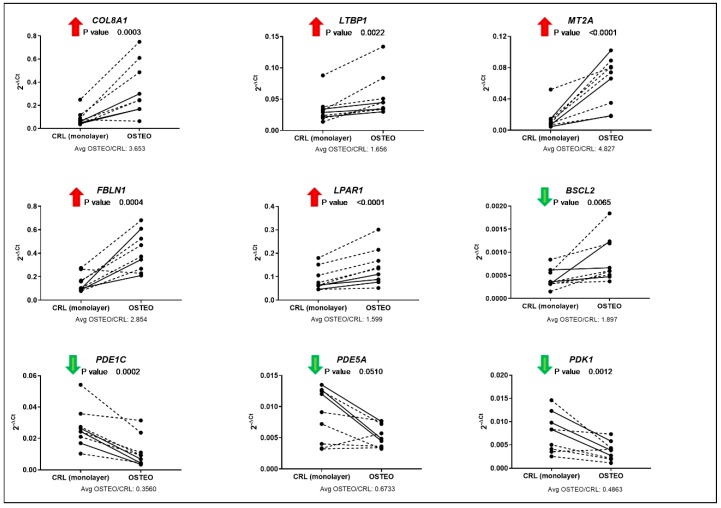
qPCR validation of linear targets corresponding to differentially expressed circular RNAs in osteogenic differentiation, as identified by microarray analysis. Full lines represent results from the same donors used for microarray, while dashed lines correspond to additional donors tested. For each gene of interest (GOI), the results are shown as 2^−ΔCt^, where ΔCt is calculated as the difference between GOI and reference gene (*RPLP0*) Ct values. The *p*-value and the average fold change between day 7 osteogenic differentiation and day 7 control (monolayer) are reported for each gene. The arrow next to the gene symbol indicates if the corresponding circRNA resulted as up- or downregulated from the microarray analysis.

As shown in Figure 3, the differential expression of total *COL8A1*, *LTBP1*, *MT2A*, *FBLN1*, *LPAR1*, *BSCL2*, *PDE1C*, and *PDK1* was confirmed by qPCR analysis for osteogenic differentiation, showing the same trend of regulation for the circular, as detected by microarrays, and total transcripts. The only exception was represented by the *BSCL2* gene, whose circular transcript was downregulated in osteogenic differentiation, but total gene expression resulted upregulated. The expression of *AGPS* and *TDRD12* did not show differential expression between control and osteogenic differentiation, while *PDE5A* showed a trend towards a downregulation even though the *p*-value remained above 0.05.

**Figure 4:**
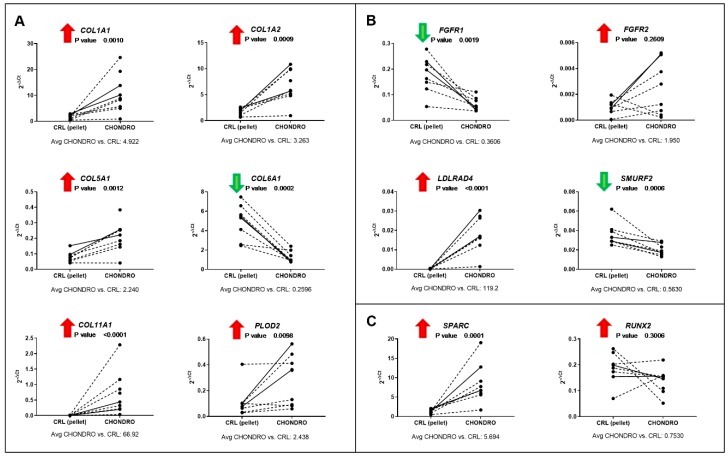
qPCR validation of linear targets corresponding to differentially expressed circular RNAs in chondrogenic differentiation and associated to (**A**) collagen fibril synthesis; (**B**) FGF and TGF-β signaling pathways; (**C**) endochondral ossification pathway. Full lines represent results from the same donors used for microarray, while dashed lines correspond to additional donors tested. For each gene of interest (GOI), the results are shown as 2^−ΔCt^, where ΔCt is calculated as the difference between GOI and reference gene (*RPLP0*) Ct values. The *p*-value and the average fold change between day 7 chondrogenic differentiation and day 7 control (pellet) expression are reported for each gene. The arrow next to the gene symbol indicates if the corresponding circRNA resulted as up- or downregulated from the microarray analysis.

In early chondrogenic commitment, the differential expression of *COL1A1*, *COL1A2*, *COL5A1*, *COL6A1*, *COL11A1*, *PLOD2*, *FGFR1*, *LDLRAD4*, *SMURF2*, and *SPARC* was validated by qPCR analysis (Figure 4). Total gene expression showed the same direction of regulation as for the circular form, i.e., an upregulated circRNA corresponded to an overall higher expression of the parental gene. 

The fold expression between osteogenic or chondrogenic differentiation and the relative controls are reported in Figure 3 and Figure 4.

### 3.5. Differential Expression of microRNA in Early Osteo- and Chondrogenesis

Analysis of RNAseq results identified 47 upregulated and 57 downregulated miRNAs in day 7 osteogenic differentiation compared to control (monolayer). During chondrogenic differentiation, 102 miRNAs were upregulated and 108 downregulated. Figure S3 shows the hierarchical clustering of miRNA expression, while Figure S4 is the volcano plot for the identification of differentially expressed miRNAs. The data are available in GEO series GSE135588 (https://www.ncbi.nlm.nih.gov/geo/query/acc.cgi?acc=GSE135588), subseries GSE135586 (https://www.ncbi.nlm.nih.gov/geo/query/acc.cgi?acc=GSE135586).

Tables S5 and S6 show the full list of differentially expressed miRNA in osteogenic and in chondrogenic differentiation, respectively, while Figure S5 reports the pathway enrichment analysis for differentially expressed miRNAs in osteogenic (Figure S5A,B) and chondrogenic differentiation (Figure S5C,D). The results showed that target genes of upregulated miRNA in osteogenic differentiation are most enriched in pathways involved in neurotransmission, calcium, insulin, and AMPK signaling and in proteoglycans-related pathways, with one of the targets being CD44. The focal adhesion pathway (KEGG hsa04510) is enriched in targets of both upregulated and downregulated miRNAs in osteogenic differentiation. For chondrogenic differentiation, it is interesting to note the enrichment in NF-kB and rheumatoid arthritis pathways for the targets of downregulated miRNA.

Table 3 reports the comparison of differentially expressed circRNA, which present binding sites for at least one of the differentially regulated miRNAs, with an exhaustive list for osteogenic differentiation. For chondrogenic differentiation, since we identified a plethora of possible interactions, only a selection of the circRNA-miRNA comparison is reported in Table 3, focusing on the circRNA derived from validated targets. 

### 3.6. Differential Expression of Piwi-Interacting RNA in Early Osteo- and Chondrogenesis

RNA sequencing identified 8 upregulated and 46 downregulated piRNA in osteogenic differentiation vs. monolayer control, while 73 were upregulated and 58 downregulated in chondrogenic differentiation (Figure S6). Tables S7 and S8 show the full list of differentially expressed piRNA in osteogenic and in chondrogenic differentiation, respectively. The data are available in GEO series GSE135588 (https://www.ncbi.nlm.nih.gov/geo/query/acc.cgi?acc=GSE135588), subseries GSE135587 (https://www.ncbi.nlm.nih.gov/geo/query/acc.cgi?acc=GSE135587).

### 3.7. Validation of miRNA and piRNA Expression

**Figure 5:**
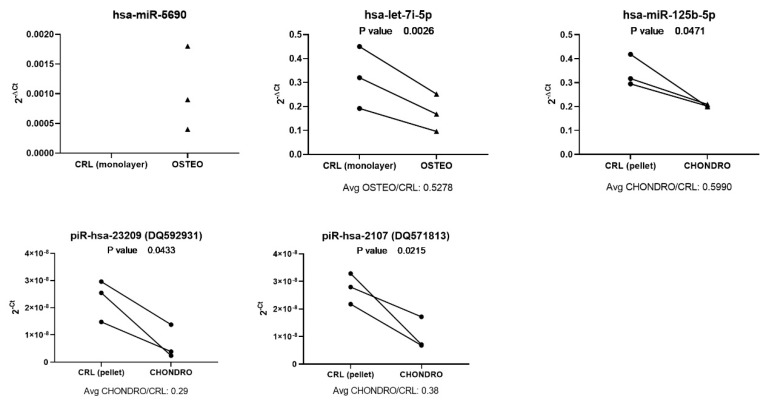
qPCR validation of miRNA and piRNA expression in early osteogenic or chondrogenic differentiation.

The expression of different miRNA and piRNA has been analyzed by qPCR to validate their expression during osteo- or chondrogenesis. RNAseq analysis showed a downregulation of let-7i-5p expression during osteogenesis. A decrease during osteogenesis was also observed from qPCR analysis, which was consistent among donors as suggested by applying a ratio paired *t*-test, which resulted in a *p*-value <0.01. In addition, the overexpression of hsa-miR-5690 was confirmed in osteogenic differentiation (Figure 5), as no expression was detectable in day 7 control (monolayer) and with Ct values between 33 and 35 for osteogenic samples. Even though the same miRNA was upregulated in early chondrogenesis as from sequencing data, in this set of samples, the differential expression of hsa-miR-5690 was not confirmed, being mostly undetectable (data not shown). Moreover, in chondrogenic differentiation, the downregulation of hsa-miR-125b-5p and piR-hsa-23209 (DQ592931) and piR-hsa-2107 (DQ571813) was also observed by qPCR analysis, in comparison to the day 7 control (pellet; Figure 5).

## 4. Discussion

In the present study, we have identified the differential expression of miRNA, piRNA, and circRNA in both osteogenic and chondrogenic differentiation of human MSCs. We have identified a set of differentially expressed circRNAs that, together with their parental gene, are implicated in a wide range of pathways important for cell fate determination, such as regulation of cyclic nucleotides for osteogenic differentiation, and collagen synthesis, FGF or TGF-β signaling pathways in the commitment towards chondrogenic differentiation. While those pathways are known to regulate MSC differentiation, the involvement of circRNA has not been previously shown, and this opens the possibility to study how circRNA can act as regulators of signaling pathways and lineage commitment. In particular, the regulation of *BSCL2* in osteogenic differentiation is worth further investigation, as the circular and the total transcript showed opposite regulation. Moreover, its potential target hsa-miR-199b-5p was upregulated in the RNAseq analysis; this miRNA has been shown before to be upregulated during osteogenic differentiation and to target GSK-3β, with a positive effect on β-catenin activation [48]. Another interesting target is represented by the *LPAR1* gene, encoding for a lysophosphatidic acid receptor, whose absence has been previously shown to decrease bone mass [49]. Furthermore, the downregulation of circular *FOXP1* in osteogenic differentiation (identified by RNAseq) is in accordance with a recent study that suggested a role for this circRNA in maintaining MSCs in a multipotent state [50]. 

The higher expression of *COL1A1* and *COL1A2* can potentially be explained with activation of pathways leading to endochondral differentiation, together with the observed upregulation of *SPARC* (which encodes for osteonectin, a bone extracellular matrix protein with affinity for collagen I and calcium). This is one of the well-known major limitations in chondrogenic pellet culture and controlling hypertrophy is one of the main challenges to tackle for cartilage tissue engineering [1]. Nevertheless, the expression of collagen type I is not limited to the hypertrophic cartilage, but it can also reflect its production in the superficial layers of the pellet. Moreover, we should keep in mind that a higher expression of the circular transcripts of *COL1A1* and *COL1A2* might regulate type I collagen fibril production. All the hypotheses are appealing but they should be tested with focused experiments and, after the initial identification of pathways, this will be one of the subjects for future research. 

The expression of *FKBP5*, *FADS2*, *ZEB1*, and *SMYD3* was evaluated in more detail. Indeed, they showed a similar regulation in osteogenic and in chondrogenic differentiation, and both differentiation protocols included dexamethasone, which was absent in the controls. The study of the expression of those genes included different steps. First, the differential expression of total *FKBP5*, *FADS2*, *ZEB1*, and *SMYD3* was assessed by qPCR. Then, their levels were also measured in linear RNA-depleted samples. Moreover, after the validation of total and circular gene expression, we tested the hypothesis according to which dexamethasone may influence their regulation. To address this research question, we tested their expression in basal monolayer or pellet cultures, with or without treatment with dexamethasone or with the nonsteroidal selective glucocorticoid receptor agonist (+)-ZK216348, which retains transrepressional, but no significant transactivational, activity [51]. 

*FKBP5* encodes for FK506 Binding Protein 5, an immunophilin that mediates the effects of immunosuppressant drugs [52]. Furthermore, it binds and inhibits the glucocorticoid receptor in a complex with heat shock proteins, regulating drug sensitivity [53,54], making it a likely candidate as target of dexamethasone in MSCs. The protein encoded by *FADS2*, Δ-6 desaturase, is the rate-limiting enzyme for the synthesis of arachidonic acid from linoleic acid [55,56]. Only a few data have been published correlating glucocorticoids and *FADS2* gene expression or protein activity; however, the effect of dexamethasone on *FADS2* transcription may be tissue- and context-dependent, while it was suggested to have an inhibitory effect on enzyme activity [55,57,58,59]. *ZEB1* is a transcriptional repressor: it inhibits *IL2* gene expression [60] and it is reported as a central component in adipocytic differentiation [61] and for the repression of stemness-inhibiting miRNAs [62]. *SMYD3* is a histone methyltransferase that activates the expression of oncogenes or cell-cycle associated genes, resulting in a proliferation of cancer cells [63]. 

Altogether, we demonstrate that total and circular *FKBP5* are consistently upregulated in both osteogenic and chondrogenic differentiation and this effect is strongly associated with dexamethasone treatment, with likely involvement of the transactivation pathway.

The circular isoforms of *ZEB1* were also upregulated in both differentiation lineages in the microarray donor cohort, with overall gene expression being significantly upregulated in early osteogenesis. For *SMYD3*, the decrease in the levels of the circular transcript did not reach statistical significance, although the trend towards a downregulation was consistent among donors. However, total *SMYD3* was significantly less expressed in both osteo- and chondrogenic differentiation. The expression of *ZEB1* and *SMYD3* appeared to be at least partially controlled by dexamethasone. *ZEB1* showed a trend towards a dexamethasone-dependent upregulation, which, however, reached statistical significance in pellet culture only. Similarly, *SMYD3* was also downregulated by the glucocorticoid in monolayer culture. For these genes, the transactivation pathway is likely involved in their regulation, as (+)-ZK216348 did not show the same effect on gene expression levels. 

On the contrary, *FADS2* total expression was not confirmed, although a donor-specific response might be hypothesized, and its expression is influenced neither by dexamethasone or (+)-ZK216348. Circular forms of *FADS2* RNA appear to be upregulated in the microarray donor cohort. Since Δ-6 desaturase is involved in the production of eicosanoids, it is possible that this may be part of a mechanism of regulation of arachidonic acid and prostaglandin production. In this context, it should be mentioned that PGE2 has been shown to increase BMP-2 expression in human MSCs [64] and that high concentrations of arachidonic acid have also been associated with the inhibition of osteoblastogenesis and promotion of adipogenesis [65]. 

Regarding miRNAs, miR-5690 was the most upregulated miRNA on day 7 of osteogenic differentiation, and this was confirmed by qPCR analysis. Among the predicted targets of this miRNA, there are different components of Wnt signaling, such as *WNT1*, *WNT3*, and *TCF7L1*. miR-5690 also targets *NOTCH3* and *OSCAR*, the latter encoding for an osteoclast-associated receptor which stimulates osteoclastogenesis. miR-5690 expression was previously shown in endothelial cells [66], but to our knowledge, it has never been associated with bone formation or cell differentiation. On the contrary, miR-125b-5p, which we showed to be downregulated in early chondrogenic differentiation, has been previously indicated to be associated with the attenuation of IL-1β target gene expression in osteoarthritic chondrocytes [67,68] and LPS-induced inflammation in ATDC5 cell line [69]. Moreover, it is downregulated by the activation of the NF-κB pathway [68] and targets *SP7* (Osterix), an important transcription factor in osteogenic differentiation [70]. It is not completely known what the role of miR-125b-5p downregulation is in chondrogenic differentiation, although it may point to an involvement of the NF-κB pathway and hypertrophy.

Other papers have identified miRNAs associated with bone differentiation. For example, in the paper by Eguchi et al. [71], the authors identified some miRNAs that can play an important role during the osteogenesis of murine and human MSCs. In particular, the miR-30 family and miR-541 expression seem to regulate the expression of various genes crucial in cell differentiation. In our case, we found that different members of the miR-30 family could be potentially targeted by circRNAs, especially those who are downregulated in osteogenic differentiation. Also, circRNA in chondrogenic differentiation potentially targets this family. Moreover, RNAseq identified miR-30a-3p as upregulated in osteogenic differentiation. As for miR-541, the authors suggest an inhibitory function of this miRNA in osteogenesis as its antagomiR may increase osteopontin and ALP expression ultimately improving calcium deposition. However, miR-541 differential expression between osteogenic differentiation and the monolayer controls was not detected in our day 7 samples.

For the circRNA–miRNA comparison, the pairs with opposite direction (upregulated circRNA with downregulated miRNA and vice versa) are especially interesting as those circRNA could have a miRNA-sponging function. In osteogenic differentiation, *FLBN1*, *MT2A*, and *BSCL2* represent three promising targets. In particular, both circular and total transcripts of *FBLN1* are overexpressed in osteogenic differentiation, and the let-7i-5p, a potential target of the circular transcript, is downregulated. In early chondrogenesis, an important role could be exerted by the circRNAs originated from *SMYD3* gene. With dexamethasone controlling *SMYD3* transcript formation at least in pellet culture, the overall transcript downregulation appears to be associated to a higher expression of miR-181a-5p, miR-181b-5p, and miR-671-5p (as identified from RNAseq). However, since SMYD3 protein has been associated to a specific transcriptional regulation of miRNA [72], whether this association is by a diminished miRNA sponging activity or not has yet to be demonstrated.

Finally, RNA sequencing identified several differentially regulated piRNAs. Even though piRNA are usually referred to as “guardians of the genome” for their role in protecting the germline from the activation of transposable elements, their regulation in somatic cells is becoming more evident in adult stem cells, cancer, and other diseases [7,11,12,13,14,16,17,18,73,74]. In this study, we have identified a total of 54 differentially expressed piRNA in osteogenic differentiation and 131 in chondrogenic differentiation. In particular, the downregulation of two piRNA in chondrogenic differentiation was observed also by qPCR analysis: piR-hsa-23209 (DQ592931, previously reported in [8,9,13,15]) and piR-hsa-2107 (DQ571813, whose expression has been shown in [8,75]). After a search in piRNA dedicated databases such as piRBase (www.piRBase.org), piRNAdb (www.pirnadb.org), and piRNAQuest (http://bicresources.jcbose.ac.in/zhumur/pirnaquest/index.html), we found that DQ592931 overlaps with the region encoding for the Ro60-associated Y4 RNA on chromosome 6. DQ571813 shows alignment in nine different genomic regions on different chromosomes, where the piRNA may lie within intronic regions of the protein-coding *VAC4* gene on chromosome 16 or associated with tRNA repeats (chromosomes 1-2-5-6-16-17). For both piRNAs, no potential target has been identified yet. Thus, functional studies will be needed to understand their role in differentiation and how to use them both as biomarkers or as functional targets for regenerative medicine strategies. Moreover, a limitation in the present piRNA validation analysis is represented by the lack of proper endogenous control for gene expression normalization. Although the RNA input was kept constant among all samples, we will need further confirmation of the results after the identification of a proper reference piRNA. Another limitation in the field of piRNA research is the lack of a standardized classification. The same piRNA could have different names based on the reference database. For example, the aliases for piRNA identified with the NCBI’s accession number DQ571813 are hsa_piRNA_30146, hsa_piR_001312, piR-31925, or piR-hsa-2107. The same limitation is identified for circRNA: it can indeed be very difficult to compare different studies on circRNA since it is not always possible to link the circRNA named according to different databases. Overall, an effort in building a common nomenclature for piRNA and circRNA and increasing the functional annotations and identification of targets will be of great benefit, helping researchers in every field to discover new mechanisms of molecular regulation. An effort in this direction for circRNA is represented by Circbank [76], which suggests a novel naming system and collects functional annotations.

## 5. Conclusions

In conclusion, we have identified various non-coding RNAs that are differentially regulated in early osteogenic and chondrogenic differentiation. A set of 4 common genes that give rise to circRNA in both osteo- and chondrogenesis led to the identification of dexamethasone-dependent targets and more information on the relative amounts of circular transcript on total gene expression. This paves the way for further investigation to understand how dexamethasone controls the expression of those genes and what their function in MSC differentiation is. Overall, functional characterization and validation for the markers herein presented will be a necessary step towards understanding their role in mesenchymal stromal cell differentiation. 

## Figures and Tables

**Table 1:** Selection of differentially-expressed circRNA in osteogenic differentiation. CircRNAs are sorted and ranked by fold change. The results of hybridization arrays identified 21 upregulated and 21 downregulated circular RNAs in osteogenic differentiation vs. undifferentiated control.

Rank	probeID	P-value	FDR	FC (abs)	Reg	circRNA	Gene Symbol	miRNA Response Elements
#1	ASCRP3005562	6.047E-08	0.000720802	5.4280811	up	hsa_circRNA_104101	*FKBP5*	hsa-miR-1468-5p hsa-miR-642a-5p hsa-miR-561-5p hsa-miR-29b-1-5p hsa-miR-708-5p
#2	ASCRP3000836	1.1812E-06	0.007039952	3.9089404	up	hsa_circRNA_002161	*FKBP5*	hsa-miR-619-5p hsa-miR-20b-3p hsa-miR-367-3p hsa-miR-153-5p hsa-miR-665
#3	ASCRP3000835	8.88526E-06	0.02118246	3.3616827	up	hsa_circRNA_076155	*FKBP5*	hsa-miR-6845-3p hsa-miR-1468-5p hsa-miR-642a-5p hsa-miR-6834-3p hsa-miR-561-5p
#4	ASCRP3000434	0.004935888	0.960907885	2.8301448	up	hsa_circRNA_022382	*FADS2*	hsa-miR-5586-5p hsa-miR-4726-5p hsa-miR-4640-5p hsa-miR-3138 hsa-miR-8080
#5	ASCRP3003129	0.019088529	0.960907885	2.8255694	up	hsa_circRNA_100833	*FADS2*	hsa-miR-765 hsa-miR-495-3p hsa-miR-665 hsa-miR-193b-5p hsa-miR-124-5p
#6	ASCRP3001707	1.86216E-06	0.007398982	2.6965752	up	hsa_circRNA_406763	*FKBP5*	hsa-miR-1273g-3p hsa-miR-1285-3p hsa-miR-619-5p hsa-miR-1183 hsa-miR-512-5p
#7	ASCRP3013118	0.003143209	0.960907885	2.304461	up	hsa_circRNA_100834	*FADS2*	hsa-miR-873-5p hsa-miR-23b-5p hsa-miR-181a-2-3p hsa-miR-93-3p hsa-miR-299-3p
#8	ASCRP3005871	0.046552395	0.960907885	2.2820422	up	hsa_circRNA_103249	*FBLN1*	hsa-miR-671-5p hsa-let-7b-5p hsa-let-7e-5p hsa-let-7i-5p hsa-miR-185-3p
#9	ASCRP3001839	0.000150493	0.172024477	2.0442825	up	hsa_circRNA_406308	*COL8A1*	hsa-miR-153-5p hsa-miR-126-5p hsa-miR-1277-5p hsa-miR-548az-5p hsa-miR-3607-3p
#10	ASCRP3013548	0.004719482	0.960907885	2.0061379	up	hsa_circRNA_405468	*MT2A*	hsa-miR-6791-5p hsa-miR-6859-5p hsa-miR-450a-1-3p hsa-miR-4257 hsa-miR-1236-5p
#12	ASCRP3009465	0.007384491	0.960907885	1.9128242	up	hsa_circRNA_057186	*AGPS*	hsa-miR-3938 hsa-miR-942-5p hsa-miR-3935 hsa-miR-646 hsa-miR-4753-3p
#13	ASCRP3007511	0.005784275	0.960907885	1.8653692	up	hsa_circRNA_100832	*FADS1*	hsa-miR-218-1-3p hsa-miR-193a-5p hsa-miR-764 hsa-miR-876-3p hsa-miR-138-5p
#14	ASCRP3009116	0.007604484	0.960907885	1.8116207	up	hsa_circRNA_405763	*TDRD12*	hsa-miR-4438 hsa-miR-182-5p hsa-miR-3188 hsa-miR-7-5p hsa-miR-1226-3p
#15	ASCRP3002616	0.035838981	0.960907885	1.6842185	up	hsa_circRNA_104864	*LPAR1*	hsa-miR-7-5p hsa-miR-588 hsa-miR-135a-3p hsa-miR-608 hsa-miR-140-3p
#16	ASCRP3003960	0.009707576	0.960907885	1.679094	up	hsa_circRNA_402206	*LTBP1*	hsa-miR-432-5p hsa-miR-6797-3p hsa-miR-3127-3p hsa-miR-6804-5p hsa-miR-18a-3p
#17	ASCRP3003038	0.011416289	0.960907885	1.6747001	up	hsa_circRNA_000782	*ZEB1*	hsa-miR-141-3p hsa-miR-200a-3p hsa-miR-148a-5p hsa-miR-136-5p hsa-miR-494-5p
#20	ASCRP3001742	0.044814485	0.960907885	1.5884727	up	hsa_circRNA_100582	*ZEB1*	hsa-miR-141-3p hsa-miR-200a-3p hsa-miR-148a-5p hsa-miR-452-3p hsa-miR-877-3p
#1	ASCRP3007208	0.002787677	0.923030978	0.4292865	down	hsa_circRNA_100512	*SMYD3*	hsa-miR-182-5p hsa-miR-181a-5p hsa-miR-532-3p hsa-miR-1224-3p hsa-miR-181b-5p
#2	ASCRP3006013	0.007116011	0.960907885	0.4726294	down	hsa_circRNA_100511	*SMYD3*	hsa-miR-532-3p hsa-miR-516a-3p hsa-miR-516b-3p hsa-miR-182-5p hsa-miR-181a-5p
#3	ASCRP3004046	0.000140327	0.172024477	0.4904122	down	hsa_circRNA_100835	*BSCL2*	hsa-miR-646 hsa-miR-199b-5p hsa-let-7g-5p hsa-let-7e-5p hsa-miR-658
#6	ASCRP3012578	7.30204E-05	0.108800441	0.5413913	down	hsa_circRNA_103729	*PDE5A*	hsa-miR-670-3p hsa-miR-583 hsa-miR-455-3p hsa-miR-510-3p hsa-miR-500a-5p
#7	ASCRP3008231	0.009384685	0.960907885	0.5464973	down	hsa_circRNA_100509	*SMYD3*	hsa-miR-516b-3p hsa-miR-516a-3p hsa-miR-671-5p hsa-miR-425-3p hsa-miR-584-5p
#8	ASCRP3007154	0.004585789	0.960907885	0.5603480	down	hsa_circRNA_100510	*SMYD3*	hsa-miR-516a-3p hsa-miR-516b-3p hsa-miR-182-5p hsa-miR-181a-5p hsa-miR-362-5p
#9	ASCRP3001523	0.002025898	0.832713912	0.5671141	down	hsa_circRNA_103415	*FOXP1*	hsa-miR-370-3p hsa-miR-558 hsa-miR-127-5p hsa-miR-93-3p hsa-miR-17-3p
#10	ASCRP3009267	0.000205329	0.180247633	0.5766464	down	hsa_circRNA_102854	*PDK1*	hsa-miR-362-5p hsa-miR-500a-5p hsa-miR-30a-5p hsa-miR-30e-5p hsa-miR-21-3p
#14	ASCRP3003982	2.29715E-05	0.045636674	0.6169628	down	hsa_circRNA_406933	*PDE1C*	hsa-miR-138-5p hsa-miR-1248 hsa-miR-3653-5p hsa-miR-6755-3p hsa-miR-26b-3p
#16	ASCRP3007600	0.009304848	0.960907885	0.6298330	down	hsa_circRNA_103414	*FOXP1*	hsa-miR-370-3p hsa-miR-558 hsa-miR-127-5p hsa-miR-93-3p hsa-miR-17-3p
#19	ASCRP3010569	0.000767043	0.481218738	0.6493059	down	hsa_circRNA_102856	*PDK1*	hsa-miR-637 hsa-miR-362-5p hsa-miR-500a-5p hsa-miR-30a-5p hsa-miR-511-5p
#21	ASCRP3000965	0.001161463	0.604800463	0.6630723	down	hsa_circRNA_102855	*PDK1*	hsa-miR-637 hsa-miR-362-5p hsa-miR-500a-5p hsa-miR-30a-5p hsa-miR-511-5p

**Table 2:** Selection of differentially expressed circRNA in chondrogenic differentiation. CircRNAs are sorted and ranked by fold change. The results of hybridization arrays identified 130 upregulated and 197 downregulated circular RNAs in osteogenic differentiation vs. undifferentiated control. The full table, including all the differentially expressed circRNA in chondrogenic differentiation, is available in the supplementary file (Table S4).

Rank	probeID	P-value	FDR	FC (abs)	Reg	circRNA	Gene Symbol	miRNA Response Elements
#1	ASCRP3005562	1.63024E-06	0.003238743	5.3480122	up	hsa_circRNA_104101	FKBP5	hsa-miR-1468-5p hsa-miR-642a-5p hsa-miR-561-5p hsa-miR-29b-1-5p hsa-miR-708-5p
#2	ASCRP3000434	0.000396713	0.055633194	4.6683484	up	hsa_circRNA_022382	FADS2	hsa-miR-5586-5p hsa-miR-4726-5p hsa-miR-4640-5p hsa-miR-3138 hsa-miR-8080
#3	ASCRP3000836	0.000102233	0.031215885	4.496583	up	hsa_circRNA_002161	FKBP5	hsa-miR-619-5p hsa-miR-20b-3p hsa-miR-367-3p hsa-miR-153-5p hsa-miR-665
#4	ASCRP3009795	1.8396E-07	0.001096402	3.9770883	up	hsa_circRNA_081069	COL1A2	hsa-miR-4733-3p hsa-miR-665 hsa-miR-5096 hsa-miR-412-3p hsa-miR-4459
#6	ASCRP3006708	7.97805E-05	0.030676897	3.7163892	up	hsa_circRNA_102121	COL1A1	hsa-miR-412-3p hsa-miR-214-3p hsa-miR-194-3p hsa-miR-761 hsa-miR-362-5p
#7	ASCRP3003129	0.000497582	0.061309307	3.5929669	up	hsa_circRNA_100833	FADS2	hsa-miR-765 hsa-miR-495-3p hsa-miR-665 hsa-miR-193b-5p hsa-miR-124-5p
#8	ASCRP3000835	0.000417696	0.057894568	3.1908719	up	hsa_circRNA_076155	FKBP5	hsa-miR-6845-3p hsa-miR-1468-5p hsa-miR-642a-5p hsa-miR-6834-3p hsa-miR-561-5p
#15	ASCRP3002750	8.236E-07	0.001963462	2.7030084	up	hsa_circRNA_400670	FGFR2	hsa-miR-449c-5p hsa-miR-885-3p hsa-miR-216b-3p hsa-miR-3147 hsa-miR-4691-3p
#16	ASCRP3009380	0.000395625	0.055633194	2.6823979	up	hsa_circRNA_400294	COL11A1	hsa-miR-4668-3p hsa-miR-4659a-3p hsa-miR-4659b-3p hsa-miR-2113 hsa-miR-548aq-3p
#18	ASCRP3004582	6.12173E-05	0.026061058	2.544377	up	hsa_circRNA_103987	SPARC	hsa-miR-194-3p hsa-miR-22-5p hsa-miR-342-5p hsa-miR-328-3p hsa-miR-30d-3p
#22	ASCRP3013118	0.0007731	0.075807131	2.2866119	up	hsa_circRNA_100834	FADS2	hsa-miR-873-5p hsa-miR-23b-5p hsa-miR-181a-2-3p hsa-miR-93-3p hsa-miR-299-3p
#23	ASCRP3001707	0.006322882	0.219733971	2.2821711	up	hsa_circRNA_406763	FKBP5	hsa-miR-1273g-3p hsa-miR-1285-3p hsa-miR-619-5p hsa-miR-1183 hsa-miR-512-5p
#26	ASCRP3004478	0.001383209	0.110656729	2.1118736	up	hsa_circRNA_102558	TGFB1	hsa-miR-219a-2-3p hsa-miR-514a-5p hsa-miR-602 hsa-miR-1301-3p hsa-miR-150-3p
#33	ASCRP3002980	0.000198815	0.036800403	2.0231353	up	hsa_circRNA_007482	COL5A1	hsa-miR-6746-5p hsa-miR-639 hsa-miR-608 hsa-miR-8082 hsa-miR-4534
#34	ASCRP3013535	0.000013325	0.015778114	2.0144106	up	hsa_circRNA_047037	LDLRAD4	hsa-miR-4767 hsa-miR-2277-5p hsa-miR-6737-3p hsa-miR-5008-3p hsa-miR-6089
#41	ASCRP3000681	1.45603E-05	0.015778114	1.9222447	up	hsa_circRNA_035152	FBN1	hsa-miR-181b-5p hsa-miR-181d-5p hsa-miR-181a-5p hsa-miR-181c-5p hsa-miR-6841-3p
#42	ASCRP3003323	0.000124468	0.032253525	1.9115005	up	hsa_circRNA_008421	FBN1	hsa-miR-519c-3p hsa-miR-519a-3p hsa-miR-519b-3p hsa-miR-6762-5p hsa-miR-136-5p
#46	ASCRP3012377	0.000184393	0.036347001	1.8787574	up	hsa_circRNA_001552	ZEB1	hsa-miR-548a-5p hsa-miR-153-5p hsa-miR-548d-5p hsa-miR-136-5p hsa-miR-568
#52	ASCRP3012658	8.81337E-05	0.031215885	1.8418985	up	hsa_circRNA_404737	ZEB1	hsa-miR-1277-5p hsa-miR-3171 hsa-miR-2054 hsa-miR-548ab hsa-miR-3149
#55	ASCRP3000740	0.000138804	0.033621593	1.822582	up	hsa_circRNA_002765	ZEB1	hsa-miR-6728-3p hsa-miR-1909-5p hsa-miR-1277-5p hsa-miR-6877-3p hsa-miR-8089
#63	ASCRP3010383	0.004955326	0.20113116	1.7629155	up	hsa_circRNA_102231	P4HB	hsa-miR-1301-3p hsa-miR-103a-2-5p hsa-miR-541-5p hsa-miR-133a-5p hsa-miR-598-5p
#71	ASCRP3008500	2.9687E-07	0.001179563	1.6937109	up	hsa_circRNA_405335	FBN1	hsa-miR-181b-5p hsa-miR-181d-5p hsa-miR-181a-5p hsa-miR-4801 hsa-miR-181c-5p
#78	ASCRP3005846	0.005602103	0.215854454	1.673856	up	hsa_circRNA_100094	HSPG2	hsa-miR-185-3p hsa-miR-671-3p hsa-miR-638 hsa-miR-675-5p hsa-miR-552-3p
#80	ASCRP3005992	0.000153855	0.034452544	1.6644833	up	hsa_circRNA_104118	RUNX2	hsa-miR-335-3p hsa-miR-216a-5p hsa-miR-450a-2-3p hsa-miR-125a-3p hsa-miR-502-5p
#91	ASCRP3003055	0.011456522	0.286111338	1.6111455	up	hsa_circRNA_402986	PLOD2	hsa-miR-6884-3p hsa-miR-7151-5p hsa-miR-6887-3p hsa-miR-6873-3p hsa-miR-1285-5p
#92	ASCRP3012598	0.008597966	0.246958447	1.6044765	up	hsa_circRNA_046265	P4HB	hsa-miR-4763-3p hsa-miR-6808-5p hsa-miR-5047 hsa-miR-4692 hsa-miR-1207-5p
#94	ASCRP3010648	0.001722361	0.12220558	1.5990799	up	hsa_circRNA_073237	VCAN	hsa-miR-4778-3p hsa-miR-122-5p hsa-miR-2115-5p hsa-miR-6854-5p hsa-miR-335-3p
#97	ASCRP3000965	0.004929519	0.20113116	1.5916847	up	hsa_circRNA_102855	PDK1	hsa-miR-637 hsa-miR-362-5p hsa-miR-500a-5p hsa-miR-30a-5p hsa-miR-511-5p
#99	ASCRP3003038	0.001851474	0.125885791	1.5899916	up	hsa_circRNA_000782	ZEB1	hsa-miR-141-3p hsa-miR-200a-3p hsa-miR-148a-5p hsa-miR-136-5p hsa-miR-494-5p
#103	ASCRP3001742	0.001136042	0.096473031	1.5793354	up	hsa_circRNA_100582	ZEB1	hsa-miR-141-3p hsa-miR-200a-3p hsa-miR-148a-5p hsa-miR-452-3p hsa-miR-877-3p
#105	ASCRP3009267	0.036604648	0.45977598	1.5758009	up	hsa_circRNA_102854	PDK1	hsa-miR-362-5p hsa-miR-500a-5p hsa-miR-30a-5p hsa-miR-30e-5p hsa-miR-21-3p
#124	ASCRP3005963	0.001148645	0.096473031	1.5235693	up	hsa_circRNA_104119	RUNX2	hsa-miR-7-5p hsa-miR-663a hsa-miR-149-3p hsa-miR-885-3p hsa-miR-661
#56	ASCRP3006502	0.002984365	0.160966674	0.5802392	down	hsa_circRNA_062035	COL6A1	hsa-miR-5001-5p hsa-miR-4685-5p hsa-miR-661 hsa-miR-4739 hsa-miR-6089
#60	ASCRP3007208	0.00578642	0.217584002	0.6086586	down	hsa_circRNA_100512	SMYD3	hsa-miR-182-5p hsa-miR-181a-5p hsa-miR-532-3p hsa-miR-1224-3p hsa-miR-181b-5p
#77	ASCRP3010338	0.005350258	0.210791919	0.6319221	down	hsa_circRNA_102171	SMURF2	hsa-miR-539-5p hsa-let-7f-2-3p hsa-miR-216a-3p hsa-miR-578 hsa-miR-587
#79	ASCRP3008231	0.011029053	0.282723243	0.6357313	down	hsa_circRNA_100509	SMYD3	hsa-miR-516b-3p hsa-miR-516a-3p hsa-miR-671-5p hsa-miR-425-3p hsa-miR-584-5p
#89	ASCRP3006013	0.003248887	0.167106571	0.6532234	down	hsa_circRNA_100511	SMYD3	hsa-miR-532-3p hsa-miR-516a-3p hsa-miR-516b-3p hsa-miR-182-5p hsa-miR-181a-5p
#91	ASCRP3013164	0.000112608	0.031215885	0.6539596	down	hsa_circRNA_083999	FGFR1	hsa-miR-6769a-5p hsa-miR-6769b-5p hsa-miR-3692-3p hsa-miR-130a-5p hsa-miR-1226-5p
#97	ASCRP3005052	0.002076144	0.135233004	0.6664074	down	hsa_circRNA_084010	FGFR1	hsa-miR-759 hsa-miR-298 hsa-miR-4435 hsa-miR-92b-5p hsa-miR-490-5p

**Table 3:** Comparison between miRNA-binding sites of differentially expressed circRNA with the list of differentially expressed miRNAs in osteogenic differentiation. The pairs that presented an opposite direction of regulation are highlighted in bold.

**CircRNA ID**	**Best Transcript**	**CircRNA Regulation in OSTEO vs. CRL Monolayer (Fold Change)**	**Binding Site For**	**miRNA Regulation in OSTEO vs. CRL Monolayer (Fold change)**
**hsa_circRNA_103249**	***FBLN1***	**Upregulated (2.28)**	**hsa-let-7i-5p**	**Downregulated (0.56)**
**hsa_circRNA_405468**	***MT2A***	**Upregulated (2.00)**	**hsa-miR-450a-1-3p**	**Downregulated (0.62)**
hsa_circRNA_406308	*COL8A1*	Upregulated (2.04)	hsa-miR-548az-5p	Upregulated (1.86)
hsa_circRNA_405763	*TDRD12*	Upregulated (1.81)	hsa-miR-7-5p	Upregulated (1.64)
hsa_circRNA_104864	*LPAR1*	Upregulated (1.68)	hsa-miR-7-5p	Upregulated (1.64)
hsa_circRNA_057186	*AGPS*	Upregulated (1.91)	hsa-miR-942-5p	Upregulated (1.59)
**hsa_circRNA_100835**	***BSCL2***	**Downregulated (0.49)**	**hsa-miR-199b-5p**	**Upregulated (2.65)**
**CircRNA ID**	**Best Transcript**	**CircRNA Regulation in CHONDRO vs. CRL Pellet (Fold Change)**	**Binding Site For**	**miRNA Regulation in CHONDRO vs. CRL Pellet (Fold Change)**
hsa_circRNA_102121	*COL1A1*	Upregulated (3.72)	hsa-miR-214-3p	Upregulated (1.85)
**hsa_circRNA_081069**	***COL1A2***	**Upregulated (3.98)**	**hsa-miR-665**	**Downregulated (0.37)**
**hsa_circRNA_100833**	***FADS2***	**Upregulated (3.59)**	**hsa-miR-665** **hsa-miR-495-3p**	**Downregulated (0.37)** **Downregulated (0.54)**
**hsa_circRNA_100834**	***FADS2***	**Upregulated (2.29)**	hsa-miR-23b-5p **hsa-miR-299-3p**	Upregulated (4.13) **Downregulated (0.61)**
hsa_circRNA_035152	*FBN1*	Upregulated (1.92)	hsa-miR-181a-5p hsa-miR-181b-5p hsa-miR-181d-5p	Upregulated (3.89) Upregulated (3.70) Upregulated (1.82)
hsa_circRNA_405335	*FBN1*	Upregulated (1.69)	hsa-miR-181a-5p hsa-miR-181b-5p hsa-miR-181d-5p	Upregulated (3.89) Upregulated (3.70) Upregulated (1.82)
hsa_circRNA_400670	*FGFR2*	Upregulated (2.70)	hsa-miR-449c-5p	Upregulated (4.81)
**hsa_circRNA_002161**	***FKBP5***	**Upregulated (4.50)**	**hsa-miR-665**	**Downregulated (0.37)**
hsa_circRNA_406763	*FKBP5*	Upregulated (2.28)	hsa-miR-1285-3p	Upregulated (1.82)
**hsa_circRNA_104101**	***FKBP5***	**Upregulated (5.35)**	**hsa-miR-29b-1-5p** **hsa-miR-708-5p**	**Downregulated (0.46)** **Downregulated (0.48)**
hsa_circRNA_100094	*HSPG2*	Upregulated (1.67)	hsa-miR-675-5p	Upregulated (24.65)
hsa_circRNA_047037	*LDLRAD4*	Upregulated (2.01)	hsa-miR-2277-5p	Upregulated (1.52)
hsa_circRNA_102854	*PDK1*	Upregulated (1.58)	hsa-miR-30e-5p hsa-miR-21-3p	Upregulated (1.56) Upregulated (2.29)
hsa_circRNA_104119	*RUNX2*	Upregulated (1.52)	hsa-miR-7-5p	Upregulated (1.42)
**hsa_circRNA_104118**	***RUNX2***	**Upregulated (1.66)**	**hsa-miR-335-3p** **hsa-miR-450a-2-3p**	**Downregulated (0.21)** **Downregulated (0.60)**
**hsa_circRNA_073237**	***VCAN***	**Upregulated (1.60)**	**hsa-miR-335-3p**	**Downregulated (0.21)**
hsa_circRNA_100582	*ZEB1*	Upregulated (1.58)	hsa-miR-148a-5p	Upregulated (2.57)
hsa_circRNA_000782	*ZEB1*	Upregulated (1.59)	hsa-miR-148a-5p	Upregulated (2.57)
**hsa_circRNA_100511**	***SMYD3***	**Downregulated (0.65)**	**hsa-miR-181a-5p**	**Upregulated (3.89)**
**hsa_circRNA_100512**	***SMYD3***	**Downregulated (0.61)**	**hsa-miR-181a-5p** **hsa-miR-181b-5p**	**Upregulated (3.89)** **Upregulated (3.70)**
**hsa_circRNA_100509**	***SMYD3***	**Downregulated (0.64)**	**hsa-miR-671-5p**	**Upregulated (1.80)**
**hsa_circRNA_102171**	***SMURF2***	**Downregulated (0.63)**	**hsa-let-7f-2-3p**	**Upregulated (1.72)**

## Supplementary Materials

The following are available online at https://www.mdpi.com/2073-4409/9/2/398/s1. Figure S1: Immunophenotype and differentiation potential of human bone marrow MSCs. Figure S2: Volcano plots were used for the visualization of differentially expressed circRNAs. Figure S3: Hierarchical clustering of differentially expressed miRNAs. Figure S4: Volcano plots were used for the visualization of differentially expressed miRNAs. Figure S5: KEGG pathway analysis for the target genes of differentially expressed miRNAs. Figure S6: Volcano plots were used for the visualization of differentially expressed piRNAs. Table S1: Summary of donor cohorts used for experiments. Table S2: List of assays used for qPCR validation of linear transcripts corresponding to differentially expressed circular RNAs. Table S3: Differentially expressed circRNA in osteogenic differentiation. Table S4: Full list of differentially expressed circRNA in chondrogenic differentiation. Table S5: Differentially expressed miRNA in osteogenic differentiation. Table S6: Differentially expressed miRNA in chondrogenic differentiation. Table S7: Differentially expressed piRNA in osteogenic differentiation. Table S8: Differentially expressed piRNA in chondrogenic differentiation.
