# Supplementary material for: Differential Regulation of circRNA, miRNA, and piRNA during Early Osteogenic and Chondrogenic Differentiation of Human Mesenchymal Stromal Cells
Source: Cells. 2020 Feb 9;9(2):398. doi: 10.3390/cells9020398 (PMC7072123; doi:10.3390/cells9020398)
Supplement: Supplementary file 1 [file cells-09-00398-s001.pdf]

A

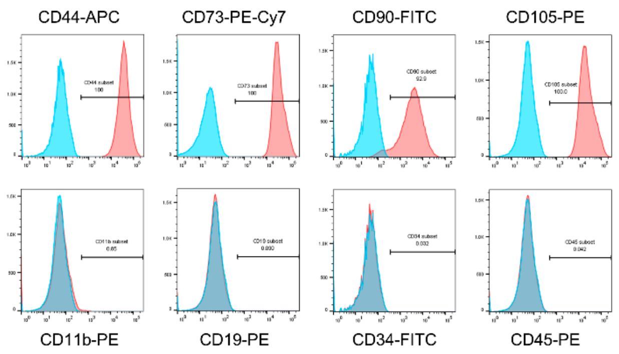

B

Donor #	%CD44	%CD73	%CD90	%CD105	%CD11b	%CD19	%CD34	%CD45
#1	100	100	92.9	100	0.85	0.030	0.032	0.042
#2	100	100	91.0	99.6	0.52	0.041	0.027	0.041
#3	100	100	95.2	100	0.91	0.030	0.038	0.27

C

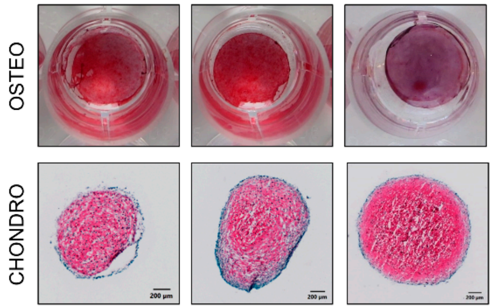

D

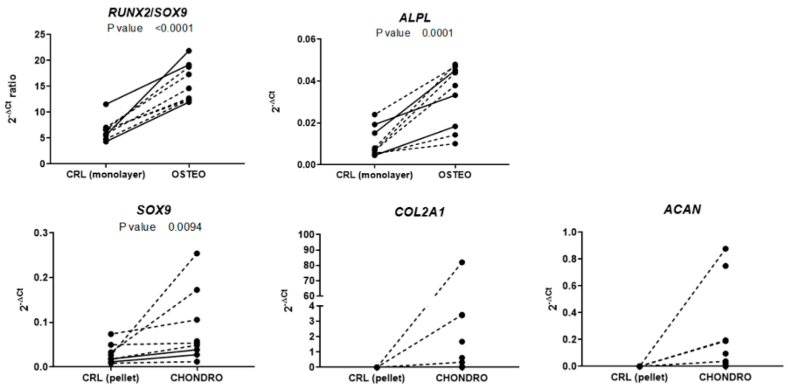

Figure S1: Immunophenotype and differentiation potential of human bone marrow MSCs. A) Representative histograms depicting flow cytometric analysis of MSCs under study. B) Summary of CD marker expression for three donors. C) Representative images for Alizarin Red staining or Safranin O/Fast green staining at day 21 for osteogenic (n=3 donors) and chondrogenic differentiation (n=3 donors), respectively. D) Analysis of day 7 gene expression of differentiation markers for all the donors included in the study. *RUNX2*/*SOX9* ratio and *ALPL* for osteogenic differentiation, and *SOX9*, *COL2A1*, and *ACAN* for chondrogenic differentiation. The expression of *COL2A1* and *ACAN* was undetectable in 5/9 and 4/9 CRL pellet samples, respectively.

Figure S2: Volcano plots were used for the identification of differentially expressed circRNAs in A) Day 7 osteogenic differentiation vs day 7 control (monolayer); and B) Day 7 chondrogenic differentiation vs day 7 control (pellet culture). X-axis represents the log2 of fold change expression (cut-off value: ± 0.585, corresponding to a fold change of 1.5), while y-axis shows the -log10 of the p-values (cut-off value: 1.30, corresponding to a p-value of 0.05). In each graph, upregulated circRNAs are represented as red squares on the right-hand side of the volcano plot, while downregulated circRNAs are represented as red squares on the left side.

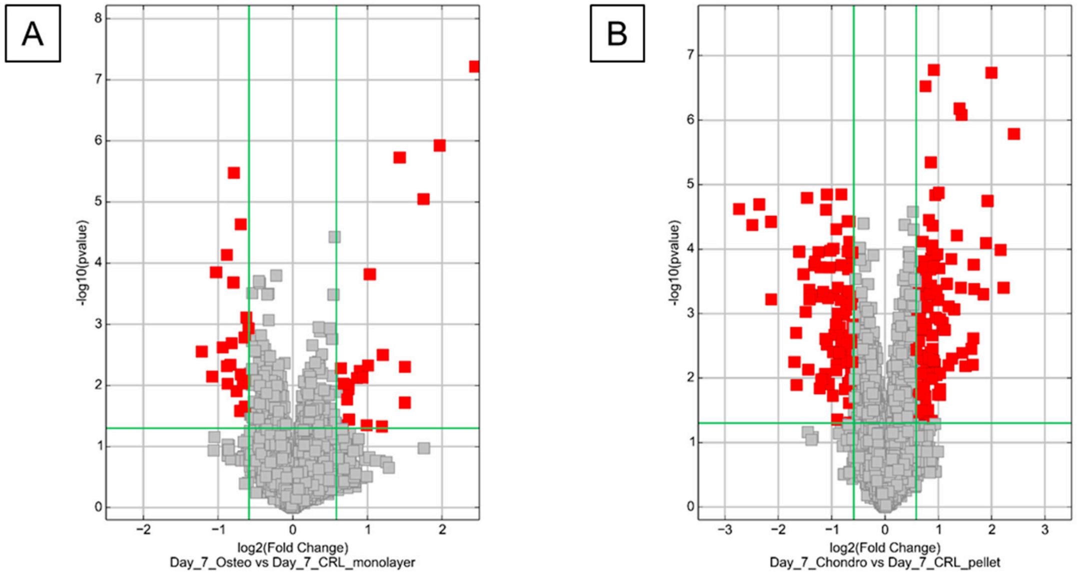

Figure S3: Hierarchical clustering of differentially expressed miRNAs. Each row represents a miRNA and each column represents a sample. A) day 7 osteogenic differentiation vs day 7 control (monolayer); B) Day 7 chondrogenic differentiation vs day 7 control (pellet culture).

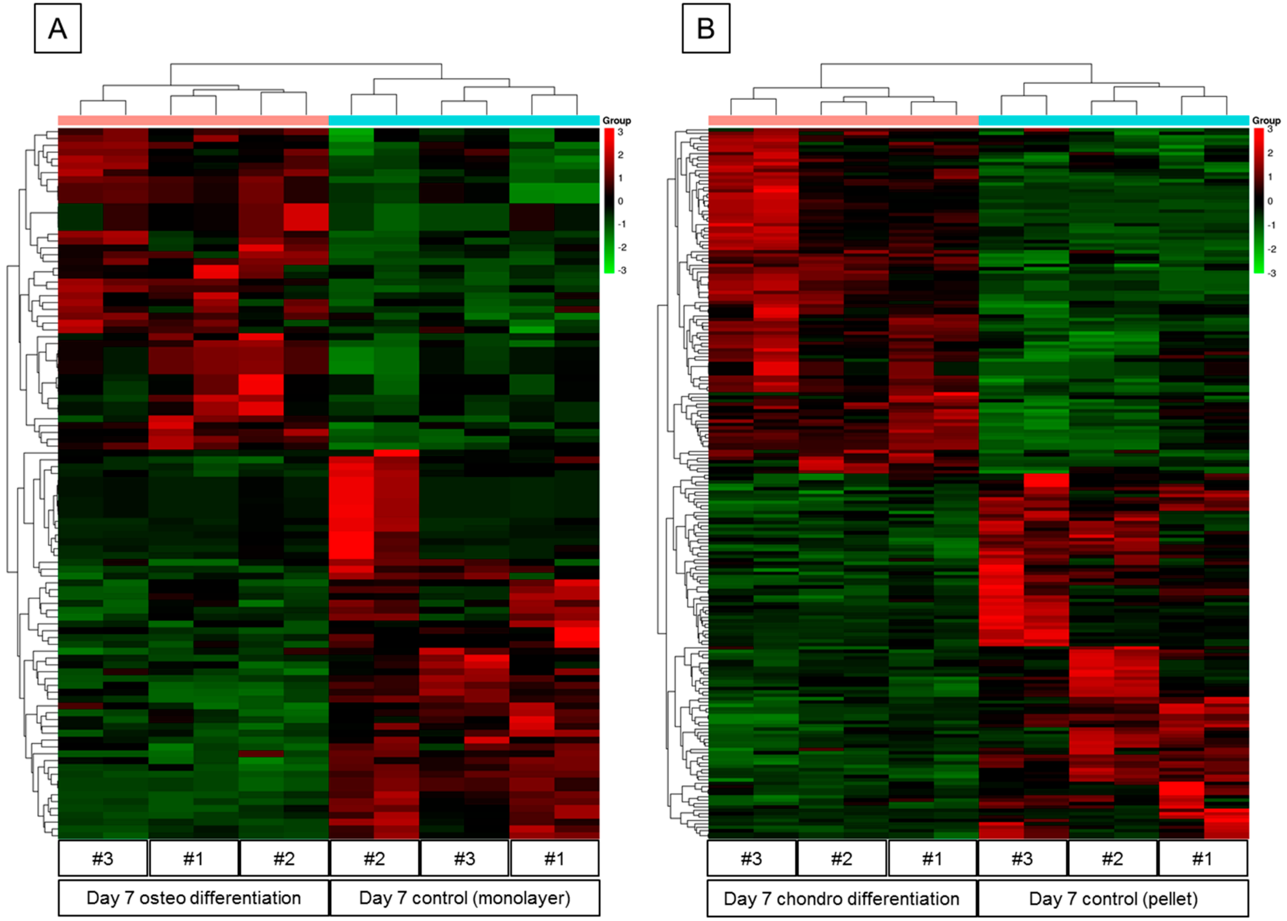

Figure S4: Volcano plots were used for the visualization of differentially expressed miRNAs in A) Day 7 osteogenic differentiation vs day 7 control (monolayer); and B) Day 7 chondrogenic differentiation vs day 7 control (pellet culture). X-axis represents the log2 of fold change expression (cut-off value: ± 0.585, corresponding to a fold change of 1.5), while y-axis shows the -log10 of the p-values (cut-off value: 1.30, corresponding to a p-value of 0.05). Upregulated miRNAs are represented as red dots on the right-hand side of the volcano plot, while downregulated miRNAs are represented as green dots on the left side.

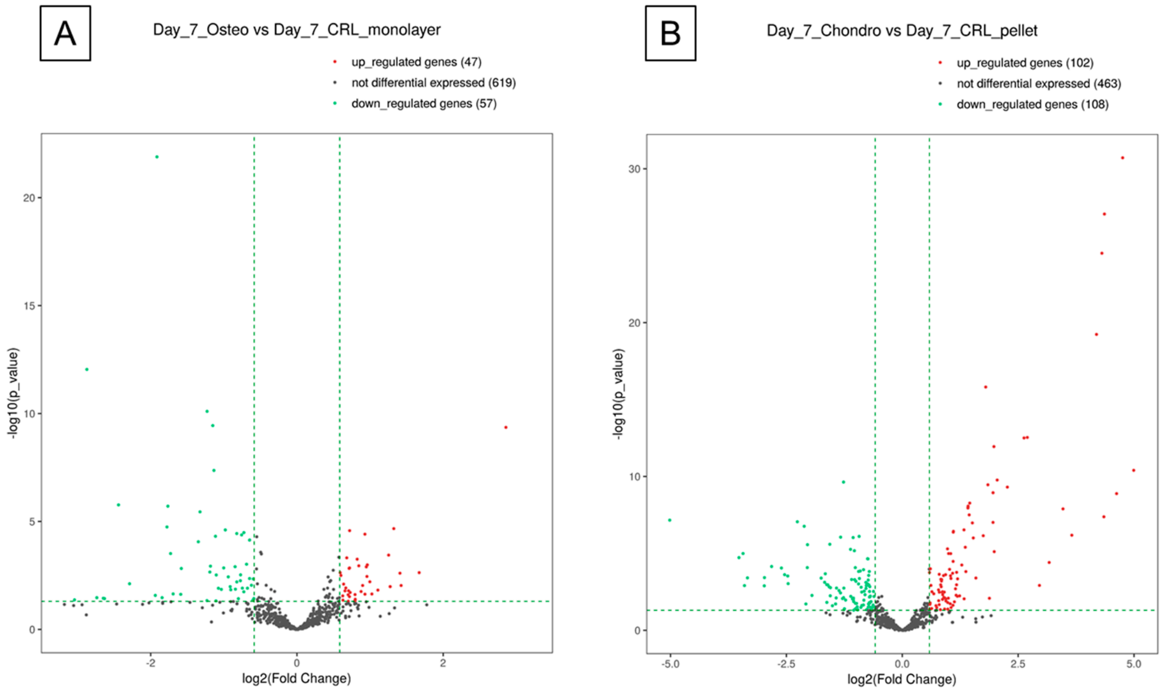

Figure S5: KEGG pathway analysis for the target genes of differentially expressed miRNAs. A) Pathway enrichment for target genes of upregulated miRNA in day 7 osteogenic differentiation vs day 7 control (monolayer); B) Pathway enrichment for target genes of downregulated miRNA in day 7 osteogenic differentiation vs day 7 control (monolayer); C) Pathway enrichment for target genes of upregulated miRNA in day 7 chondrogenic differentiation vs day 7 control (pellet culture); D) Pathway enrichment for target genes of downregulated miRNA in day 7 chondrogenic differentiation vs day 7 control (pellet culture). The bar plots show the top ten enrichment score (-log10(P-value)) value of the significant pathways. The enrichment p-value of the pathway ID used Fisher's exact test for calculation. Pathways are ordered from top to bottom by p-value, with the most significant pathway on the top.

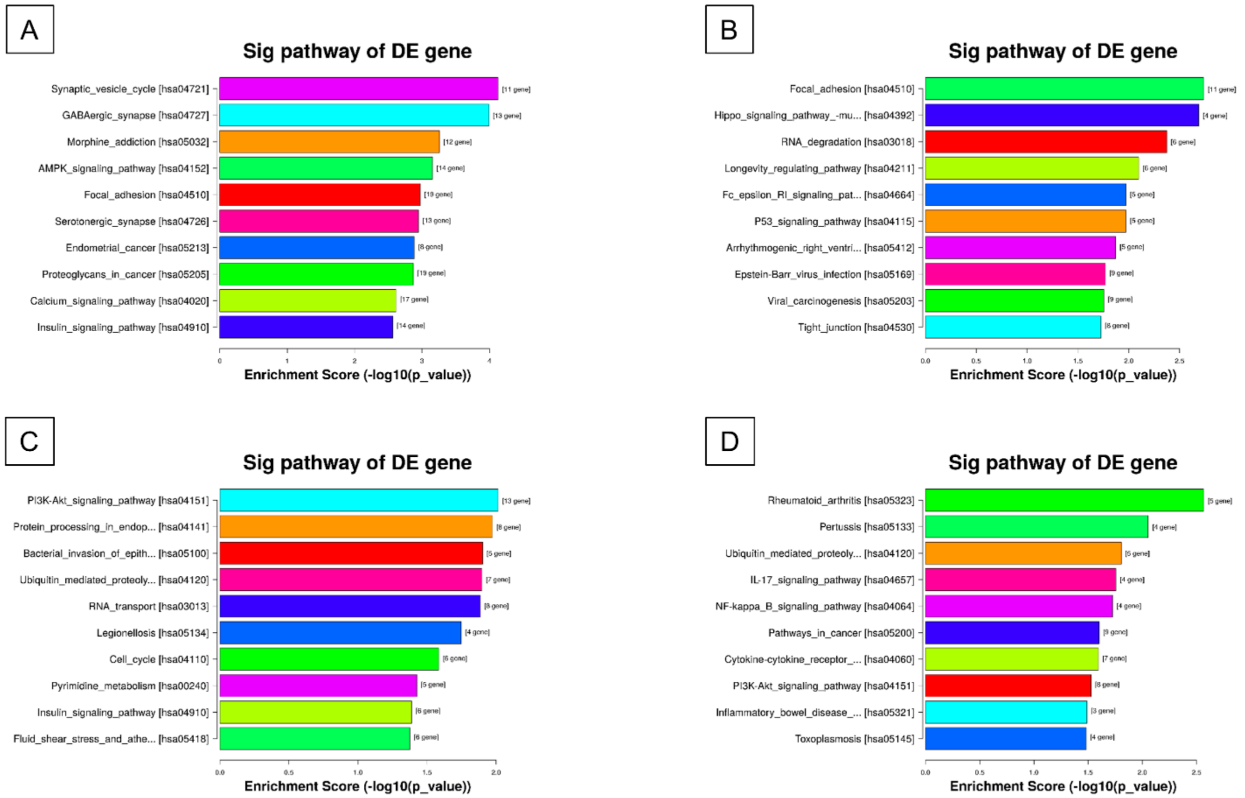

Figure S6: Volcano plots were used for the visualization of differentially expressed piRNAs in A) Day 7 osteogenic differentiation vs day 7 control (monolayer); and B) Day 7 chondrogenic differentiation vs day 7 control (pellet culture). X-axis represents the log2 of fold change expression (cut-off value: ± 0.585, corresponding to a fold change of 1.5), while y-axis shows the -log10 of the p-values (cut-off value: 1.30, corresponding to a p-value of 0.05). Upregulated piRNAs are represented as red dots on the right-hand side of the volcano plot, while downregulated piRNAs are represented as blue dots on the left side.

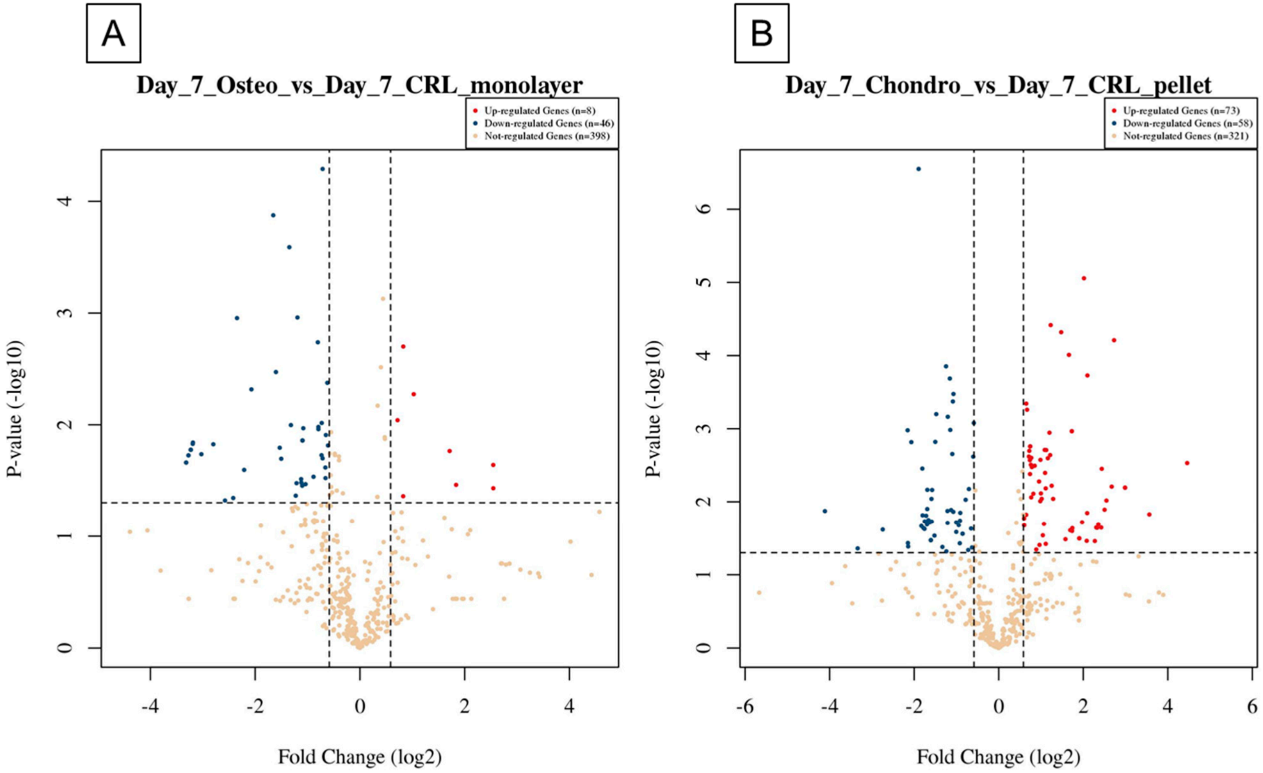

Table S1: Summary of donor cohorts used for experiments.

**Donor #**	#1, #2, #3	#4, #5, #6, #7, #8, #9 (osteogenic differentiation)<br>#4, #5, #6, #10, #11, #12 (chondrogenic differentiation)	#13, #14, #15, #16
**Experiment type**	Immunophenotype<br>Sequencing/arrays<br>Validation (total transcript)<br>Validation (circular transcripts)<br>Validation (miRNA-piRNA)	Validation (total transcripts)<br>Day 7 differentiation markers<br>Day 21 differentiation	Effect of dexamethasone only on total gene expression
**Figure no.**	2 A-C; 3; 4 A-C; 5; S1 A-B-D	2 A; 3; 4 A-C; S1 C-D	2D

Table S2 - List of assays used for gene expression analysis. Probe modification was 5'-FAM and 3'-NFQ-MGB for assays on demand, while 5'-FAM and 3'-TAMRA for other genes.

**ASSAYS ON DEMAND**		
**Gene**	**Assay ID**	**Transcripts identified**
AGPS	Hs01056969_m1	NM_003659.3
ALPL	Hs00758162_m1	NM_000478.5; NM_001127501.3; NM_001177520.2
BSCL2	Hs00949220_m1	NM_001130702.2
COL1A2	Hs01028956_m1	NM_000089.3
COL5A1	Hs00609088_m1	NM_000093.4; NM_001278074.1
COL6A1	Hs01095585_m1	NM_001848.2
COL8A1	Hs00156669_m1	NM_001850.4; NM_020351.3
COL11A1	Hs01097664_m1	NM_001190709.1; NM_001854.3; NM_080629.2; NM_080630.3
FADS2	Hs00927433_m1	NM_001281501.1; NM_001281502.1; NM_004265.3
FBLN1	Hs00972609_m1	NM_001996.3; NM_006485.3; NM_006486.2; NM_006487.2
FBN1	Hs00171191_m1	NM_000138.4
FGFR1	Hs00241111_m1	NM_001174063.1; NM_001174064.1; NM_001174065.1; NM_001174066.1; NM_001174067.1; NM_015850.3; NM_023105.2; NM_023106.2; NM_023110.2
FGFR2	Hs01552918_m1	NM_000141.4; NM_001144913.1; NM_001144914.1; NM_001144915.1; NM_001144916.1; NM_001144917.1; NM_001144918.1; NM_001144919.1; NM_001320654.1; NM_001320658.1; NM_022970.3; NM_023029.2
FKBP5	Hs01561006_m1	NM_001145775.2; NM_001145776.1; NM_001145777.1; NM_004117.3
HSPG2	Hs01078536_m1	NM_001291860.1; NM_005529.6
LDLRAD4	Hs00188966_m1	NM_001003674.3; NM_001276249.1; NM_001276251.1; NM_181481.4
LPAR1	Hs00173500_m1	NM_001401.3; NM_057159.2

LTBP1	Hs01558763_m1	NM_000627.3; NM_001166264.1; NM_001166265.1; NM_001166266.1; NM_206943.2
MT2A	Hs02379661_g1	NM_005953.3
P4HB	Hs01050257_m1	NM_000918.3
PDE1C	Hs01095682_m1	NM_001191056.3; NM_001191057.3; NM_001191058.3; NM_001191059.3; NM_001322055.1; NM_001322056.1; NM_001322057.1; NM_001322058.1; NM_001322059.1; NM_005020.4
PDE5A	Hs00153649_m1	NM_001083.3; NM_033430.2; NM_033437.3
PDK1	Hs01561847_m1	NM_001278549.1; NM_002610.4
PLOD2	Hs01118190_m1	NM_000935.2; NM_182943.2
SMURF2	Hs00224203_m1	NM_022739.3
SMYD3	Hs01585866_m1	NM_001167740.1; NM_022743.2
SOX9	Hs00165814_m1	NM_000346.3
SPARC	Hs00234160_m1	NM_001309443.1; NM_001309444.1; NM_003118.3
TDRD12	Hs00415527_m1	XM_011527471.2; XM_011527472.2; XM_011527473.2; XM_011527474.2; XM_011527475.2; XM_011527476.2; XM_017027458.1; XM_017027459.1
TGFB1	Hs00171257_m1	NM_000660.5
VCAN	Hs00171642_m1	NM_001126336.2; NM_001164097.1; NM_001164098.1; NM_004385.4
ZEB1	Hs01566408_m1	NM_001128128.2; NM_001174093.1; NM_001174094.1; NM_001174095.1; NM_001174096.1; NM_001323638.1; NM_001323641.1; NM_001323642.1; NM_001323643.1; NM_001323644.1; NM_001323645.1; NM_001323646.1; NM_001323647.1; NM_001323648.1; NM_001323649.1; NM_001323650.1; NM_001323651.1; NM_001323652.1; NM_001323653.1; NM_001323654.1; NM_001323655.1; NM_001323656.1; NM_001323657.1; NM_001323658.1; NM_001323659.1; NM_001323660.1; NM_001323661.1; NM_001323662.1; NM_001323663.1; NM_001323664.1; NM_001323665.1; NM_001323666.1; NM_001323671.1; NM_001323672.1; NM_001323673.1; NM_001323674.1; NM_001323675.1; NM_001323676.1; NM_001323677.1; NM_001323678.1; NM_030751.5

OTHER ASSAYS

**Gene**	**Forward primer**	**Reverse primer**	**Probe**	**Transcripts identified**
RPLP0	5'-TGGGCAAGAACACCATGATG-3'	5'-CGGATATGAGGCAGCAGTTTC-3'	5'-AGGGCACCTGGAAAACAACCCAGC-3'	NM_001002.4; NM_053275.3
ACAN	5'-AGTCCTCAAGCCTCCTGTACTCA-3'	5'-CGGGAAGTGGCGGTAACA-3'	5'-CCGGAATGGAAACGTGAATCAGAATCAACT-3'	NM_001369268.1; NM_001135.3; NM_013227.3
COL1A1	5'-CCCTGGAAAGAATGGAGATGAT-3'	5'-ACTGAAACCTCTGTGTCCCTTCA-3'	5'-CGGGCAATCCTCGAGCACCCT-3'	NM_000088.3
COL2A1	5'-GGCAATAGCAGGTTCACGTACA-3'	5'-GATAACAGTCTTGCCCCACTTACC-3'	5'-CCTGAAGGATGGCTGCACGAAACATAC-3'	NM_033150.3; NM_001844.5
RUNX2	5'-AGCAAGGTTCAACGATCTGAGAT-3'	5'-TTTGTGAAGACGGTTATGGTCAA-3'	5'-TGAAACTCTTGCCTCGTCCACTCCG-3'	NM_001024630.4; NM_001015051.3

Table S3 – Differentially expressed circRNA in osteogenic differentiation. CircRNAs are sorted by fold change.

Rank	probeID	P-value	FDR	FC (abs)	Reg	circRNA	Alias	Gene Symbol	miRNA response elements
#1	ASCRP3005562	6.047E-08	0.000720802	5.4280811	up	hsa_circRNA_104101	hsa_circ_0001599	*FKBP5*	hsa-miR-1468-5p<br>hsa-miR-642a-5p<br>hsa-miR-561-5p<br>hsa-miR-29b-1-5p<br>hsa-miR-708-5p
#2	ASCRP3000836	1.1812E-06	0.007039952	3.9089404	up	hsa_circRNA_002161	hsa_circ_0001600	*FKBP5*	hsa-miR-619-5p<br>hsa-miR-20b-3p<br>hsa-miR-367-3p<br>hsa-miR-153-5p<br>hsa-miR-665
#3	ASCRP3000835	8.88526E-06	0.02118246	3.3616827	up	hsa_circRNA_076155	hsa_circ_0076155	*FKBP5*	hsa-miR-6845-3p<br>hsa-miR-1468-5p<br>hsa-miR-642a-5p<br>hsa-miR-6834-3p<br>hsa-miR-561-5p
#4	ASCRP3000434	0.004935888	0.960907885	2.8301448	up	hsa_circRNA_022382	hsa_circ_0022382	*FADS2*	hsa-miR-5586-5p<br>hsa-miR-4726-5p<br>hsa-miR-4640-5p<br>hsa-miR-3138<br>hsa-miR-8080
#5	ASCRP3003129	0.019088529	0.960907885	2.8255694	up	hsa_circRNA_100833	hsa_circ_0022383	*FADS2*	hsa-miR-765<br>hsa-miR-495-3p<br>hsa-miR-665<br>hsa-miR-193b-5p<br>hsa-miR-124-5p
#6	ASCRP3001707	1.86216E-06	0.007398982	2.6965752	up	hsa_circRNA_406763		*FKBP5*	hsa-miR-1273g-3p<br>hsa-miR-1285-3p<br>hsa-miR-619-5p<br>hsa-miR-1183<br>hsa-miR-512-5p
#7	ASCRP3013118	0.003143209	0.960907885	2.304461	up	hsa_circRNA_100834	hsa_circ_0022392	*FADS2*	hsa-miR-873-5p<br>hsa-miR-23b-5p<br>hsa-miR-181a-2-3p<br>hsa-miR-93-3p<br>hsa-miR-299-3p
#8	ASCRP3005871	0.046552395	0.960907885	2.2820422	up	hsa_circRNA_103249	hsa_circ_0063756	*FBLN1*	hsa-miR-671-5p<br>hsa-let-7b-5p<br>hsa-let-7e-5p<br>hsa-let-7i-5p<br>hsa-miR-185-3p
#9	ASCRP3001839	0.000150493	0.172024477	2.0442825	up	hsa_circRNA_406308		*COL8A1*	hsa-miR-153-5p<br>hsa-miR-126-5p<br>hsa-miR-1277-5p<br>hsa-miR-548az-5p<br>hsa-miR-3607-3p
#10	ASCRP3013548	0.004719482	0.960907885	2.0061379	up	hsa_circRNA_405468		*MT2A*	hsa-miR-6791-5p<br>hsa-miR-6859-5p<br>hsa-miR-450a-1-3p<br>hsa-miR-4257<br>hsa-miR-1236-5p

#11	ASCRP3007140	0.044845909	0.960907885	1.9820909	up	hsa_circRNA_406174		*PITPNB*	hsa-miR-4739<br>hsa-miR-6797-5p<br>hsa-miR-6878-5p<br>hsa-miR-4716-3p<br>hsa-miR-4668-5p
#12	ASCRP3009465	0.007384491	0.960907885	1.9128242	up	hsa_circRNA_057186	hsa_circ_0057186	*AGPS*	hsa-miR-3938<br>hsa-miR-942-5p<br>hsa-miR-3935<br>hsa-miR-646<br>hsa-miR-4753-3p
#13	ASCRP3007511	0.005784275	0.960907885	1.8653692	up	hsa_circRNA_100832	hsa_circ_0022378	*FADS1*	hsa-miR-218-1-3p<br>hsa-miR-193a-5p<br>hsa-miR-764<br>hsa-miR-876-3p<br>hsa-miR-138-5p
#14	ASCRP3009116	0.007604484	0.960907885	1.8116207	up	hsa_circRNA_405763		*TDRD12*	hsa-miR-4438<br>hsa-miR-182-5p<br>hsa-miR-3188<br>hsa-miR-7-5p<br>hsa-miR-1226-3p
#15	ASCRP3002616	0.035838981	0.960907885	1.6842185	up	hsa_circRNA_104864	hsa_circ_0003611	*LPAR1*	hsa-miR-7-5p<br>hsa-miR-588<br>hsa-miR-135a-3p<br>hsa-miR-608<br>hsa-miR-140-3p
#16	ASCRP3003960	0.009707576	0.960907885	1.679094	up	hsa_circRNA_402206		*LTBP1*	hsa-miR-432-5p<br>hsa-miR-6797-3p<br>hsa-miR-3127-3p<br>hsa-miR-6804-5p<br>hsa-miR-18a-3p
#17	ASCRP3003038	0.011416289	0.960907885	1.6747001	up	hsa_circRNA_000782	hsa_circ_0000230	*ZEB1*	hsa-miR-141-3p<br>hsa-miR-200a-3p<br>hsa-miR-148a-5p<br>hsa-miR-136-5p<br>hsa-miR-494-5p
#18	ASCRP3007483	0.01687908	0.960907885	1.652923	up	hsa_circRNA_100497	hsa_circ_0017105	*NID1*	hsa-miR-765<br>hsa-miR-892a<br>hsa-miR-24-3p<br>hsa-miR-671-5p<br>hsa-miR-323a-5p
#19	ASCRP3008599	0.009374291	0.960907885	1.6022994	up	hsa_circRNA_103629	hsa_circ_0005991	*APBB2*	hsa-miR-30b-3p<br>hsa-miR-329-5p<br>hsa-miR-762<br>hsa-miR-422a<br>hsa-miR-378a-3p
#20	ASCRP3001742	0.044814485	0.960907885	1.5884727	up	hsa_circRNA_100582	hsa_circ_0004126	*ZEB1*	hsa-miR-141-3p<br>hsa-miR-200a-3p<br>hsa-miR-148a-5p<br>hsa-miR-452-3p<br>hsa-miR-877-3p
#21	ASCRP3001008	0.005187968	0.960907885	1.5636082	up	hsa_circRNA_103912	hsa_circ_0007747	*NR2F1-AS1*	hsa-miR-590-3p<br>hsa-miR-29b-1-5p<br>hsa-miR-587<br>hsa-miR-563<br>hsa-miR-330-5p

Rank	probeID	P-value	FDR	FC (abs)	Reg	circRNA	Alias	Gene Symbol	miRNA response elements
#1	ASCRP3007208	0.002787677	0.923030978	0.4292865	down	hsa_circRNA_100512	hsa_circ_0005090	*SMYD3*	hsa-miR-182-5p<br>hsa-miR-181a-5p<br>hsa-miR-532-3p<br>hsa-miR-1224-3p<br>hsa-miR-181b-5p
#2	ASCRP3006013	0.007116011	0.960907885	0.4726294	down	hsa_circRNA_100511	hsa_circ_0017289	*SMYD3*	hsa-miR-532-3p<br>hsa-miR-516a-3p<br>hsa-miR-516b-3p<br>hsa-miR-182-5p<br>hsa-miR-181a-5p
#3	ASCRP3004046	0.000140327	0.172024477	0.4904122	down	hsa_circRNA_100835	hsa_circ_0022502	*BSCL2*	hsa-miR-646<br>hsa-miR-199b-5p<br>hsa-let-7g-5p<br>hsa-let-7e-5p<br>hsa-miR-658
#4	ASCRP3000993	0.002372084	0.856825663	0.5218541	down	hsa_circRNA_104981	hsa_circ_0007933	*MID1*	hsa-miR-612<br>hsa-miR-888-5p<br>hsa-miR-216a-3p<br>hsa-miR-891a-3p<br>hsa-miR-597-3p
#5	ASCRP3008340	0.004827189	0.960907885	0.5413269	down	hsa_circRNA_002415	hsa_circ_0002415	*MID1*	hsa-miR-6721-5p<br>hsa-miR-6879-5p<br>hsa-miR-4436b-3p<br>hsa-miR-612<br>hsa-miR-4632-5p
#6	ASCRP3012578	7.30204E-05	0.108800441	0.5413913	down	hsa_circRNA_103729	hsa_circ_0002474	*PDE5A*	hsa-miR-670-3p<br>hsa-miR-583<br>hsa-miR-455-3p<br>hsa-miR-510-3p<br>hsa-miR-500a-5p
#7	ASCRP3008231	0.009384685	0.960907885	0.5464973	down	hsa_circRNA_100509	hsa_circ_0017286	*SMYD3*	hsa-miR-516b-3p<br>hsa-miR-516a-3p<br>hsa-miR-671-5p<br>hsa-miR-425-3p<br>hsa-miR-584-5p
#8	ASCRP3007154	0.004585789	0.960907885	0.5603480	down	hsa_circRNA_100510	hsa_circ_0017287	*SMYD3*	hsa-miR-516a-3p<br>hsa-miR-516b-3p<br>hsa-miR-182-5p<br>hsa-miR-181a-5p<br>hsa-miR-362-5p
#9	ASCRP3001523	0.002025898	0.832713912	0.5671141	down	hsa_circRNA_103415	hsa_circ_0008234	*FOXP1*	hsa-miR-370-3p<br>hsa-miR-558<br>hsa-miR-127-5p<br>hsa-miR-93-3p<br>hsa-miR-17-3p
#10	ASCRP3009267	0.000205329	0.180247633	0.5766464	down	hsa_circRNA_102854	hsa_circ_0057104	*PDK1*	hsa-miR-362-5p<br>hsa-miR-500a-5p<br>hsa-miR-30a-5p<br>hsa-miR-30e-5p<br>hsa-miR-21-3p
#11	ASCRP3006814	3.33654E-06	0.009942889	0.5795771	down	hsa_circRNA_001063	hsa_circ_0001063		hsa-miR-4698<br>hsa-miR-5095<br>hsa-miR-30c-5p

									hsa-miR-30b-5p<br>hsa-miR-30d-5p
#12	ASCRP3006806	0.01233779	0.960907885	0.5945437	down	hsa_circRNA_104566	hsa_circ_0004458	*PSD3*	hsa-miR-450b-5p<br>hsa-miR-205-3p<br>hsa-miR-95-5p<br>hsa-miR-329-5p<br>hsa-miR-19b-2-5p
#13	ASCRP3000367	0.026065507	0.960907885	0.6138192	down	hsa_circRNA_404643		*PIK3C2B*	hsa-miR-762<br>hsa-miR-4641<br>hsa-miR-6775-5p<br>hsa-miR-5090<br>hsa-miR-1224-5p
#14	ASCRP3003982	2.29715E-05	0.045636674	0.6169628	down	hsa_circRNA_406933		*PDE1C*	hsa-miR-138-5p<br>hsa-miR-1248<br>hsa-miR-3653-5p<br>hsa-miR-6755-3p<br>hsa-miR-26b-3p
#15	ASCRP3010519	0.006560581	0.960907885	0.6180983	down	hsa_circRNA_003552	hsa_circ_0003552	*MOCOS*	hsa-miR-3064-5p<br>hsa-miR-330-5p<br>hsa-miR-3921<br>hsa-miR-4303<br>hsa-miR-519d-5p
#16	ASCRP3007600	0.009304848	0.960907885	0.6298330	down	hsa_circRNA_103414	hsa_circ_0001320	*FOXP1*	hsa-miR-370-3p<br>hsa-miR-558<br>hsa-miR-127-5p<br>hsa-miR-93-3p<br>hsa-miR-17-3p
#17	ASCRP3003293	0.001644417	0.753902085	0.6392945	down	hsa_circRNA_104815	hsa_circ_0006702	*IARS*	hsa-miR-29b-3p<br>hsa-miR-29a-3p<br>hsa-miR-22-5p<br>hsa-miR-371a-5p<br>hsa-miR-29c-3p
#18	ASCRP3007618	0.022220401	0.960907885	0.6410978	down	hsa_circRNA_101063	hsa_circ_0026302	*CSRNP2*	hsa-miR-612<br>hsa-miR-485-5p<br>hsa-miR-571<br>hsa-miR-212-5p<br>hsa-miR-764
#19	ASCRP3010569	0.000767043	0.481218738	0.6493059	down	hsa_circRNA_102856	hsa_circ_0006006	*PDK1*	hsa-miR-637<br>hsa-miR-362-5p<br>hsa-miR-500a-5p<br>hsa-miR-30a-5p<br>hsa-miR-511-5p
#20	ASCRP3007619	0.00830555	0.960907885	0.6620863	down	hsa_circRNA_101062	hsa_circ_0004538	*CSRNP2*	hsa-miR-612<br>hsa-miR-212-5p<br>hsa-miR-764<br>hsa-let-7f-2-3p<br>hsa-miR-660-3p
#21	ASCRP3000965	0.001161463	0.604800463	0.6630723	down	hsa_circRNA_102855	hsa_circ_0057105	*PDK1*	hsa-miR-637<br>hsa-miR-362-5p<br>hsa-miR-500a-5p<br>hsa-miR-30a-5p<br>hsa-miR-511-5p

Table S4 – Full list of differentially expressed circRNA in chondrogenic differentiation.

Rank	probeID	P-value	FDR	FC (abs)	Reg	circRNA	Alias	Gene Symbol	miRNA response elements
#1	ASCRP3005562	1.63024E-06	0.003238743	5.3480122	up	hsa_circRNA_104101	hsa_circ_0001599	*FKBP5*	hsa-miR-1468-5p<br>hsa-miR-642a-5p<br>hsa-miR-561-5p<br>hsa-miR-29b-1-5p<br>hsa-miR-708-5p
#2	ASCRP3000434	0.000396713	0.055633194	4.6683484	up	hsa_circRNA_022382	hsa_circ_0022382	*FADS2*	hsa-miR-5586-5p<br>hsa-miR-4726-5p<br>hsa-miR-4640-5p<br>hsa-miR-3138<br>hsa-miR-8080
#3	ASCRP3000836	0.000102233	0.031215885	4.496583	up	hsa_circRNA_002161	hsa_circ_0001600	*FKBP5*	hsa-miR-619-5p<br>hsa-miR-20b-3p<br>hsa-miR-367-3p<br>hsa-miR-153-5p<br>hsa-miR-665
#4	ASCRP3009795	1.8396E-07	0.001096402	3.9770883	up	hsa_circRNA_081069	hsa_circ_0081069	*COL1A2*	hsa-miR-4733-3p<br>hsa-miR-665<br>hsa-miR-5096<br>hsa-miR-412-3p<br>hsa-miR-4459
#5	ASCRP3011615	1.79071E-05	0.016419442	3.7959286	up	hsa_circRNA_400633		*SCD*	hsa-miR-5589-5p<br>hsa-miR-1253<br>hsa-miR-3127-3p<br>hsa-miR-541-3p<br>hsa-miR-3691-3p
#6	ASCRP3006708	7.97805E-05	0.030676897	3.7163892	up	hsa_circRNA_102121	hsa_circ_0044556	*COL1A1*	hsa-miR-412-3p<br>hsa-miR-214-3p<br>hsa-miR-194-3p<br>hsa-miR-761<br>hsa-miR-362-5p
#7	ASCRP3003129	0.000497582	0.061309307	3.5929669	up	hsa_circRNA_100833	hsa_circ_0022383	*FADS2*	hsa-miR-765<br>hsa-miR-495-3p<br>hsa-miR-665<br>hsa-miR-193b-5p<br>hsa-miR-124-5p
#8	ASCRP3000835	0.000417696	0.057894568	3.1908719	up	hsa_circRNA_076155	hsa_circ_0076155	*FKBP5*	hsa-miR-6845-3p<br>hsa-miR-1468-5p<br>hsa-miR-642a-5p<br>hsa-miR-6834-3p<br>hsa-miR-561-5p
#9	ASCRP3005569	0.000173807	0.036347001	3.1659751	up	hsa_circRNA_050900	hsa_circ_0050900	*ACTN4*	hsa-miR-4678<br>hsa-miR-3667-3p<br>hsa-miR-6891-3p<br>hsa-miR-605-3p<br>hsa-miR-6507-3p
#10	ASCRP3010238	0.002407994	0.141134705	3.1462404	up	hsa_circRNA_080272	hsa_circ_0080272	*CCT6A*	hsa-miR-148b-3p<br>hsa-miR-152-3p<br>hsa-miR-148a-3p<br>hsa-miR-205-3p<br>hsa-miR-802

#11	ASCRP3006887	0.00618324	0.219733971	3.1226231	up	hsa_circRNA_051239	hsa_circ_0051239	*ATP5SL*	hsa-miR-6867-5p<br>hsa-miR-574-5p<br>hsa-miR-6799-5p<br>hsa-miR-3162-5p<br>hsa-miR-4739
#12	ASCRP3006491	0.0035179	0.173121225	3.0722204	up	hsa_circRNA_050898	hsa_circ_0050898	*ACTN4*	hsa-miR-3909<br>hsa-miR-4691-5p<br>hsa-miR-1226-5p<br>hsa-miR-6792-3p<br>hsa-miR-6762-3p
#13	ASCRP3002950	0.006522384	0.223713111	2.845595	up	hsa_circRNA_101002	hsa_circ_0025201	*GAPDH*	hsa-miR-382-5p<br>hsa-miR-212-5p<br>hsa-miR-410-5p<br>hsa-miR-490-5p
#14	ASCRP3009896	0.004092455	0.181538185	2.728959	up	hsa_circRNA_102116	hsa_circ_0003258	*ZNF652*	hsa-miR-520d-3p<br>hsa-miR-500a-5p<br>hsa-miR-518c-3p<br>hsa-miR-103a-2-5p<br>hsa-miR-518f-3p
#15	ASCRP3002750	8.236E-07	0.001963462	2.7030084	up	hsa_circRNA_400670		*FGFR2*	hsa-miR-449c-5p<br>hsa-miR-885-3p<br>hsa-miR-216b-3p<br>hsa-miR-3147<br>hsa-miR-4691-3p
#16	ASCRP3009380	0.000395625	0.055633194	2.6823979	up	hsa_circRNA_400294		*COL11A1*	hsa-miR-4668-3p<br>hsa-miR-4659a-3p<br>hsa-miR-4659b-3p<br>hsa-miR-2113<br>hsa-miR-548aq-3p
#17	ASCRP3007811	6.5711E-07	0.001958188	2.630363	up	hsa_circRNA_000551	hsa_circ_0000551	*SLC8A3*	hsa-miR-6868-3p<br>hsa-miR-1226-3p<br>hsa-miR-5193<br>hsa-miR-7161-3p<br>hsa-miR-3667-3p
#18	ASCRP3004582	6.12173E-05	0.026061058	2.544377	up	hsa_circRNA_103987	hsa_circ_0004104	*SPARC*	hsa-miR-194-3p<br>hsa-miR-22-5p<br>hsa-miR-342-5p<br>hsa-miR-328-3p<br>hsa-miR-30d-3p
#19	ASCRP3008303	0.000860449	0.080760259	2.4555827	up	hsa_circRNA_003567	hsa_circ_0003567	*ARHGEF19*	hsa-miR-1184<br>hsa-miR-6813-5p<br>hsa-miR-6846-5p<br>hsa-miR-3135b<br>hsa-miR-6085
#20	ASCRP3009903	0.004973687	0.20113116	2.3900741	up	hsa_circRNA_404808		*CNNM2*	hsa-miR-619-5p<br>hsa-miR-1303<br>hsa-miR-5787<br>hsa-miR-1285-3p<br>hsa-miR-645
#21	ASCRP3003332	0.00014103	0.033621593	2.3694258	up	hsa_circRNA_058196	hsa_circ_0058196	*IGFBP5*	hsa-miR-6734-3p<br>hsa-miR-4723-3p<br>hsa-miR-4651<br>hsa-miR-6766-5p<br>hsa-miR-6731-5p

#22	ASCRP3013118	0.0007731	0.075807131	2.2866119	up	hsa_circRNA_100834	hsa_circ_0022392	*FADS2*	hsa-miR-873-5p<br>hsa-miR-23b-5p<br>hsa-miR-181a-2-3p<br>hsa-miR-93-3p<br>hsa-miR-299-3p
#23	ASCRP3001707	0.006322882	0.219733971	2.2821711	up	hsa_circRNA_406763		*FKBP5*	hsa-miR-1273g-3p<br>hsa-miR-1285-3p<br>hsa-miR-619-5p<br>hsa-miR-1183<br>hsa-miR-512-5p
#24	ASCRP3005836	0.000343326	0.051727177	2.228761	up	hsa_circRNA_400724		*ANO5*	hsa-miR-2392<br>hsa-miR-6790-5p<br>hsa-miR-6830-3p<br>hsa-miR-3185<br>hsa-miR-6832-3p
#25	ASCRP3011466	0.001784567	0.124946705	2.1697675	up	hsa_circRNA_100770	hsa_circ_0021506	*ANO5*	hsa-miR-19b-2-5p<br>hsa-miR-19b-1-5p<br>hsa-miR-767-3p<br>hsa-miR-506-5p<br>hsa-miR-550a-3p
#26	ASCRP3004478	0.001383209	0.110656729	2.1118736	up	hsa_circRNA_102558	hsa_circ_0051218	*TGFB1*	hsa-miR-219a-2-3p<br>hsa-miR-514a-5p<br>hsa-miR-602<br>hsa-miR-1301-3p<br>hsa-miR-150-3p
#27	ASCRP3004032	0.001511479	0.115492518	2.1108296	up	hsa_circRNA_009618	hsa_circ_0009618	*ENO1*	hsa-miR-3157-5p<br>hsa-miR-6131<br>hsa-miR-6729-3p<br>hsa-miR-616-3p<br>hsa-miR-8083
#28	ASCRP3008865	0.017199139	0.338865686	2.0496219	up	hsa_circRNA_104597	hsa_circ_0084021	*PLEKHA2*	hsa-miR-659-3p<br>hsa-miR-548d-5p<br>hsa-miR-548c-5p<br>hsa-miR-651-3p<br>hsa-miR-548a-5p
#29	ASCRP3000121	0.018262477	0.347190958	2.0492794	up	hsa_circRNA_104385	hsa_circ_0006365	*TPST1*	hsa-miR-182-5p<br>hsa-miR-329-5p<br>hsa-miR-100-3p<br>hsa-miR-221-3p<br>hsa-miR-450b-3p
#30	ASCRP3000301	0.014745732	0.314434929	2.0471734	up	hsa_circRNA_003251	hsa_circ_0003251	*WNK1*	hsa-miR-301a-5p<br>hsa-miR-3191-5p<br>hsa-miR-761<br>hsa-miR-6805-5p<br>hsa-miR-4753-3p
#31	ASCRP3006649	0.001218636	0.100180294	2.0403323	up	hsa_circRNA_101842	hsa_circ_0007669	*SLC7A6*	hsa-miR-489-3p<br>hsa-miR-892b<br>hsa-miR-449c-5p<br>hsa-miR-665<br>hsa-miR-138-5p
#32	ASCRP3007140	0.008308972	0.244444956	2.0391947	up	hsa_circRNA_406174		*PITPNB*	hsa-miR-4739<br>hsa-miR-6797-5p<br>hsa-miR-6878-5p<br>hsa-miR-4716-3p<br>hsa-miR-4668-5p

#33	ASCRP3002980	0.000198815	0.036800403	2.0231353	up	hsa_circRNA_007482	hsa_circ_0007482	*COL5A1*	hsa-miR-6746-5p<br>hsa-miR-639<br>hsa-miR-608<br>hsa-miR-8082<br>hsa-miR-4534
#34	ASCRP3013535	0.000013325	0.015778114	2.0144106	up	hsa_circRNA_047037	hsa_circ_0047037	*LDLRAD4*	hsa-miR-4767<br>hsa-miR-2277-5p<br>hsa-miR-6737-3p<br>hsa-miR-5008-3p<br>hsa-miR-6089
#35	ASCRP3004566	0.000609916	0.066207613	1.9974046	up	hsa_circRNA_104099	hsa_circ_0076092	*SCUBE3*	hsa-miR-514a-3p<br>hsa-miR-203a-5p<br>hsa-miR-609<br>hsa-miR-449a<br>hsa-miR-449b-5p
#36	ASCRP3000476	0.018081851	0.345964142	1.986192	up	hsa_circRNA_087856	hsa_circ_0087856	*RAD23B*	hsa-miR-3692-5p<br>hsa-miR-6511b-5p<br>hsa-miR-4763-3p<br>hsa-miR-4518<br>hsa-miR-1266-5p
#37	ASCRP3013051	0.000119367	0.032253525	1.9765448	up	hsa_circRNA_006859	hsa_circ_0006859	*ENC1*	hsa-miR-335-3p<br>hsa-miR-3192-5p<br>hsa-miR-486-3p<br>hsa-miR-4692<br>hsa-miR-4498
#38	ASCRP3006591	0.000434972	0.058918981	1.955376	up	hsa_circRNA_076094	hsa_circ_0076094	*SCUBE3*	hsa-miR-941<br>hsa-miR-203a-5p<br>hsa-miR-6728-3p<br>hsa-miR-6805-3p<br>hsa-miR-4322
#39	ASCRP3004508	0.008994938	0.253508895	1.9524743	up	hsa_circRNA_104854	hsa_circ_0087862	*RAD23B*	hsa-miR-325<br>hsa-miR-593-3p<br>hsa-miR-512-3p<br>hsa-miR-653-5p<br>hsa-miR-766-5p
#40	ASCRP3003319	0.001157353	0.096473031	1.9298021	up	hsa_circRNA_007924	hsa_circ_0007924	*SAMD4A*	hsa-miR-6842-5p<br>hsa-miR-3135b<br>hsa-miR-608<br>hsa-miR-6752-5p<br>hsa-miR-1226-5p
#41	ASCRP3000681	1.45603E-05	0.015778114	1.9222447	up	hsa_circRNA_035152	hsa_circ_0035152	*FBN1*	hsa-miR-181b-5p<br>hsa-miR-181d-5p<br>hsa-miR-181a-5p<br>hsa-miR-181c-5p<br>hsa-miR-6841-3p
#42	ASCRP3003323	0.000124468	0.032253525	1.9115005	up	hsa_circRNA_008421	hsa_circ_0008421	*FBN1*	hsa-miR-519c-3p<br>hsa-miR-519a-3p<br>hsa-miR-519b-3p<br>hsa-miR-6762-5p<br>hsa-miR-136-5p
#43	ASCRP3005476	0.006131903	0.219733971	1.9103775	up	hsa_circRNA_019120	hsa_circ_0019120	*BTAF1*	hsa-miR-20b-3p<br>hsa-miR-5009-5p<br>hsa-miR-8058<br>hsa-miR-96-5p<br>hsa-miR-2355-3p

#44	ASCRP3006225	0.008275468	0.244444956	1.8866843	up	hsa_circRNA_063603	hsa_circ_0063603	*CCDC134*	hsa-miR-583<br>hsa-miR-1286<br>hsa-miR-6514-3p<br>hsa-miR-4673<br>hsa-miR-6735-3p
#45	ASCRP3003705	1.6525E-07	0.001096402	1.8849185	up	hsa_circRNA_051641	hsa_circ_0051641	*AP2S1*	hsa-miR-4685-5p<br>hsa-miR-5196-5p<br>hsa-miR-6089<br>hsa-let-7d-5p<br>hsa-miR-4747-5p
#46	ASCRP3012377	0.000184393	0.036347001	1.8787574	up	hsa_circRNA_001552	hsa_circ_0000229	*ZEB1*	hsa-miR-548a-5p<br>hsa-miR-153-5p<br>hsa-miR-548d-5p<br>hsa-miR-136-5p<br>hsa-miR-568
#47	ASCRP3012860	0.005358217	0.210791919	1.8736437	up	hsa_circRNA_103717	hsa_circ_0070617	*PAPSS1*	hsa-miR-520a-5p<br>hsa-miR-338-3p<br>hsa-miR-628-5p<br>hsa-miR-525-5p<br>hsa-miR-370-3p
#48	ASCRP3003765	4.28254E-05	0.020419141	1.8586598	up	hsa_circRNA_042437	hsa_circ_0042437	*SPECC1*	hsa-miR-6751-5p<br>hsa-miR-1182<br>hsa-miR-4787-5p<br>hsa-miR-8081<br>hsa-miR-3692-3p
#49	ASCRP3009986	0.003526107	0.173121225	1.8512586	up	hsa_circRNA_102402	hsa_circ_0048232	*DAZAP1*	hsa-miR-433-3p<br>hsa-miR-324-5p<br>hsa-miR-215-3p<br>hsa-miR-670-5p<br>hsa-miR-562
#50	ASCRP3005592	0.000101061	0.031215885	1.8468093	up	hsa_circRNA_076479	hsa_circ_0076479	*PTK7*	hsa-miR-3064-5p<br>hsa-miR-608<br>hsa-miR-5787<br>hsa-miR-4474-3p<br>hsa-miR-485-3p
#51	ASCRP3001381	0.002174269	0.137128528	1.8426934	up	hsa_circRNA_005882	hsa_circ_0005882	*STK39*	hsa-miR-4677-5p<br>hsa-miR-3160-5p<br>hsa-miR-6832-3p<br>hsa-miR-5003-3p<br>hsa-miR-103a-2-5p
#52	ASCRP3012658	8.81337E-05	0.031215885	1.8418985	up	hsa_circRNA_404737		*ZEB1*	hsa-miR-1277-5p<br>hsa-miR-3171<br>hsa-miR-2054<br>hsa-miR-548ab<br>hsa-miR-3149
#53	ASCRP3011119	0.010322763	0.271578899	1.8378136	up	hsa_circRNA_101003	hsa_circ_0025202	*GAPDH*	hsa-miR-194-3p<br>hsa-miR-212-5p<br>hsa-miR-449a<br>hsa-miR-34c-5p<br>hsa-miR-449b-5p
#54	ASCRP3006411	0.046072256	0.506585391	1.834815	up	hsa_circRNA_005812	hsa_circ_0005812	*AKT2*	hsa-miR-1-5p<br>hsa-miR-3130-3p<br>hsa-miR-4537<br>hsa-miR-4440<br>hsa-miR-130b-3p

#55	ASCRP3000740	0.000138804	0.033621593	1.822582	up	hsa_circRNA_002765	hsa_circ_0002765	*ZEB1*	hsa-miR-6728-3p<br>hsa-miR-1909-5p<br>hsa-miR-1277-5p<br>hsa-miR-6877-3p<br>hsa-miR-8089
#56	ASCRP3000889	0.000362634	0.052714578	1.8145884	up	hsa_circRNA_403286		*TRIP13*	hsa-miR-7974<br>hsa-miR-34a-3p<br>hsa-miR-6072<br>hsa-miR-6864-3p<br>hsa-miR-409-5p
#57	ASCRP3003764	4.47207E-06	0.007615296	1.8138909	up	hsa_circRNA_003545	hsa_circ_0003545	*SPECC1*	hsa-miR-6751-5p<br>hsa-miR-637<br>hsa-miR-6803-5p<br>hsa-miR-1182<br>hsa-miR-365a-5p
#58	ASCRP3008766	0.003947925	0.179735849	1.8067623	up	hsa_circRNA_104853	hsa_circ_0087861	*RAD23B*	hsa-miR-325<br>hsa-miR-593-3p<br>hsa-miR-512-3p<br>hsa-miR-766-5p<br>hsa-miR-28-5p
#59	ASCRP3007794	0.000206848	0.036800403	1.8025183	up	hsa_circRNA_103553	hsa_circ_0001377	*TNK2*	hsa-miR-508-5p<br>hsa-miR-665<br>hsa-miR-125a-3p<br>hsa-miR-24-3p<br>hsa-miR-1301-3p
#60	ASCRP3005602	0.000499834	0.061309307	1.7798365	up	hsa_circRNA_005044	hsa_circ_0005044	*PRR5L*	hsa-miR-665<br>hsa-miR-1285-3p<br>hsa-miR-6511a-5p<br>hsa-miR-4739<br>hsa-miR-6848-5p
#61	ASCRP3008067	3.50685E-05	0.020419141	1.7661931	up	hsa_circRNA_007719	hsa_circ_0007719	*CREB3L1*	hsa-miR-1248<br>hsa-miR-4778-3p<br>hsa-miR-6739-3p<br>hsa-miR-4279<br>hsa-miR-6780a-3p
#62	ASCRP3010790	0.009770335	0.264120629	1.7635126	up	hsa_circRNA_407254		*RXRA*	hsa-miR-4660<br>hsa-miR-3173-3p<br>hsa-miR-6717-5p<br>hsa-miR-6891-5p<br>hsa-miR-761
#63	ASCRP3010383	0.004955326	0.20113116	1.7629155	up	hsa_circRNA_102231	hsa_circ_0046263	*P4HB*	hsa-miR-1301-3p<br>hsa-miR-103a-2-5p<br>hsa-miR-541-5p<br>hsa-miR-133a-5p<br>hsa-miR-598-5p
#64	ASCRP3011336	0.031140462	0.440815869	1.7512013	up	hsa_circRNA_404433		*KIF1B*	hsa-miR-8065<br>hsa-miR-1294<br>hsa-miR-338-3p<br>hsa-miR-192-3p<br>hsa-miR-616-3p
#65	ASCRP3006888	0.032204398	0.444551468	1.7401319	up	hsa_circRNA_051238	hsa_circ_0051238	*ATP5SL*	hsa-miR-6867-5p<br>hsa-miR-574-5p<br>hsa-miR-6799-5p<br>hsa-miR-3162-5p<br>hsa-miR-4739

#66	ASCRP3003839	0.000780162	0.075807131	1.7353084	up	hsa_circRNA_103802	hsa_circ_0071991	*MYO10*	hsa-miR-892b<br>hsa-miR-512-5p<br>hsa-miR-450a-2-3p<br>hsa-miR-767-3p<br>hsa-miR-625-5p
#67	ASCRP3007861	0.010271564	0.271578899	1.7257946	up	hsa_circRNA_092447	hsa_circ_0000807	*SEPT9*	hsa-miR-4685-5p<br>hsa-miR-6836-5p<br>hsa-miR-3918<br>hsa-miR-6735-5p<br>hsa-miR-6764-5p
#68	ASCRP3001255	0.001452632	0.113812049	1.7034892	up	hsa_circRNA_004516	hsa_circ_0004516	*FAM216A*	hsa-miR-5047<br>hsa-miR-1301-3p<br>hsa-miR-3925-3p<br>hsa-miR-4666b<br>hsa-miR-4710
#69	ASCRP3007862	0.008488206	0.246779051	1.7031857	up	hsa_circRNA_000808	hsa_circ_0000808	*SEPT9*	hsa-miR-4685-5p<br>hsa-miR-6836-5p<br>hsa-miR-3918<br>hsa-miR-6735-5p<br>hsa-miR-6764-5p
#70	ASCRP3010538	0.000277766	0.045985724	1.7016049	up	hsa_circRNA_051640	hsa_circ_0051640	*AP2S1*	hsa-miR-4685-5p<br>hsa-miR-5196-5p<br>hsa-miR-4747-5p<br>hsa-miR-4685-3p<br>hsa-miR-5589-5p
#71	ASCRP3008500	2.9687E-07	0.001179563	1.6937109	up	hsa_circRNA_405335		*FBN1*	hsa-miR-181b-5p<br>hsa-miR-181d-5p<br>hsa-miR-181a-5p<br>hsa-miR-4801<br>hsa-miR-181c-5p
#72	ASCRP3009669	0.019785511	0.356924942	1.6936245	up	hsa_circRNA_104170	hsa_circ_0006404	*FOXO3*	hsa-miR-138-5p<br>hsa-miR-143-3p<br>hsa-miR-185-3p<br>hsa-miR-30c-1-3p<br>hsa-miR-762
#73	ASCRP3000479	0.00593686	0.218417816	1.6926753	up	hsa_circRNA_105023	hsa_circ_0002153	*MID2*	hsa-miR-125a-3p<br>hsa-miR-623<br>hsa-miR-598-5p<br>hsa-miR-541-3p<br>hsa-miR-342-5p
#74	ASCRP3009990	0.029144585	0.427426003	1.6853874	up	hsa_circRNA_405330		*PDIA3*	hsa-miR-3942-3p<br>hsa-miR-6844<br>hsa-miR-335-3p<br>hsa-miR-559<br>hsa-miR-548as-5p
#75	ASCRP3005633	0.000233803	0.040984219	1.6849159	up	hsa_circRNA_104372	hsa_circ_0008334	*GRB10*	hsa-miR-485-5p<br>hsa-miR-450a-2-3p<br>hsa-miR-187-3p<br>hsa-miR-423-5p<br>hsa-miR-182-3p
#76	ASCRP3003063	0.019020072	0.354620772	1.6830535	up	hsa_circRNA_102424	hsa_circ_0004720	*KDM4B*	hsa-miR-197-3p<br>hsa-miR-487a-3p<br>hsa-miR-524-5p<br>hsa-miR-154-3p<br>hsa-miR-323a-5p

#77	ASCRP3008577	0.000190553	0.036347001	1.6802753	up	hsa_circRNA_100042	hsa_circ_0009610	*ENO1*	hsa-miR-593-5p<br>hsa-miR-874-5p<br>hsa-let-7a-2-3p<br>hsa-miR-345-3p<br>hsa-miR-148a-3p
#78	ASCRP3005846	0.005602103	0.215854454	1.673856	up	hsa_circRNA_100094	hsa_circ_0010735	*HSPG2*	hsa-miR-185-3p<br>hsa-miR-671-3p<br>hsa-miR-638<br>hsa-miR-675-5p<br>hsa-miR-552-3p
#79	ASCRP3007282	0.023887278	0.387396404	1.6722959	up	hsa_circRNA_001880	hsa_circ_0001880	*RAD23B*	hsa-miR-4691-5p<br>hsa-miR-6792-3p<br>hsa-miR-548ax<br>hsa-miR-4691-3p<br>hsa-miR-548ao-5p
#80	ASCRP3005992	0.000153855	0.034452544	1.6644833	up	hsa_circRNA_104118	hsa_circ_0003563	*RUNX2*	hsa-miR-335-3p<br>hsa-miR-216a-5p<br>hsa-miR-450a-2-3p<br>hsa-miR-125a-3p<br>hsa-miR-502-5p
#81	ASCRP3013585	0.014121406	0.308169445	1.6590365	up	hsa_circRNA_005230	hsa_circ_0005230	*DNM3OS*	hsa-miR-548n<br>hsa-miR-4668-5p<br>hsa-miR-548ab<br>hsa-miR-3145-3p<br>hsa-miR-651-3p
#82	ASCRP3000655	0.005645943	0.215854454	1.649242	up	hsa_circRNA_007878	hsa_circ_0007878	*MAP4*	hsa-miR-212-5p<br>hsa-miR-362-5p<br>hsa-miR-6842-3p<br>hsa-miR-196a-5p<br>hsa-miR-6508-3p
#83	ASCRP3001419	0.000206664	0.036800403	1.6384689	up	hsa_circRNA_025177	hsa_circ_0025177	*GAPDH*	hsa-miR-4763-3p<br>hsa-miR-1207-5p<br>hsa-miR-6766-5p<br>hsa-miR-1275<br>hsa-miR-5698
#84	ASCRP3000599	0.031078966	0.440547223	1.6370963	up	hsa_circRNA_100363	hsa_circ_0014717	*CCT3*	hsa-miR-181a-2-3p<br>hsa-miR-378a-3p<br>hsa-miR-422a<br>hsa-miR-378d<br>hsa-miR-342-5p
#85	ASCRP3007586	0.013979813	0.306763318	1.6292787	up	hsa_circRNA_074595	hsa_circ_0074595	*ANXA6*	hsa-miR-4435<br>hsa-miR-3677-5p<br>hsa-miR-3167<br>hsa-miR-4469<br>hsa-miR-4642
#86	ASCRP3005915	0.000075735	0.030615042	1.6261506	up	hsa_circRNA_101997	hsa_circ_0042428	*SPECC1*	hsa-miR-1301-3p<br>hsa-miR-9-5p<br>hsa-miR-511-5p<br>hsa-miR-637<br>hsa-miR-627-3p
#87	ASCRP3002838	0.020658468	0.361822535	1.6247111	up	hsa_circRNA_102379	hsa_circ_0047821	*ZNF532*	hsa-miR-216a-3p<br>hsa-miR-646<br>hsa-miR-27a-3p<br>hsa-miR-27b-3p<br>hsa-miR-365b-5p

#88	ASCRP3000378	0.000251713	0.042513503	1.6228171	up	hsa_circRNA_103462	hsa_circ_0007841	*SEC61A1*	hsa-miR-29b-2-5p<br>hsa-miR-19b-1-5p<br>hsa-miR-185-3p<br>hsa-miR-1296-3p<br>hsa-miR-501-5p
#89	ASCRP3012967	0.031512201	0.441245674	1.6157221	up	hsa_circRNA_404163		*DCAF12*	hsa-miR-4756-3p<br>hsa-miR-520a-5p<br>hsa-miR-5010-3p<br>hsa-miR-525-5p<br>hsa-miR-4677-5p
#90	ASCRP3007084	0.043885174	0.495248236	1.6136429	up	hsa_circRNA_028628	hsa_circ_0028628	*RFC5*	hsa-miR-2355-3p<br>hsa-miR-6844<br>hsa-miR-4665-5p<br>hsa-miR-769-3p<br>hsa-miR-3144-5p
#91	ASCRP3003055	0.011456522	0.286111338	1.6111455	up	hsa_circRNA_402986		*PLOD2*	hsa-miR-6884-3p<br>hsa-miR-7151-5p<br>hsa-miR-6887-3p<br>hsa-miR-6873-3p<br>hsa-miR-1285-5p
#92	ASCRP3012598	0.008597966	0.246958447	1.6044765	up	hsa_circRNA_046265	hsa_circ_0046265	*P4HB*	hsa-miR-4763-3p<br>hsa-miR-6808-5p<br>hsa-miR-5047<br>hsa-miR-4692<br>hsa-miR-1207-5p
#93	ASCRP3000694	0.031416503	0.441073912	1.6042749	up	hsa_circRNA_040698	hsa_circ_0040698	*USP10*	hsa-miR-4685-5p<br>hsa-miR-4446-3p<br>hsa-miR-637<br>hsa-miR-888-3p<br>hsa-miR-7843-5p
#94	ASCRP3010648	0.001722361	0.12220558	1.5990799	up	hsa_circRNA_073237	hsa_circ_0073237	*VCAN*	hsa-miR-4778-3p<br>hsa-miR-122-5p<br>hsa-miR-2115-5p<br>hsa-miR-6854-5p<br>hsa-miR-335-3p
#95	ASCRP3004220	0.000559455	0.063511492	1.5979113	up	hsa_circRNA_102179	hsa_circ_0007580	*PRKCA*	hsa-miR-516b-5p<br>hsa-miR-1301-3p<br>hsa-miR-598-3p<br>hsa-miR-188-3p<br>hsa-miR-203a-5p
#96	ASCRP3010370	0.004631226	0.193021715	1.5951343	up	hsa_circRNA_102107	hsa_circ_0008586	*KPNB1*	hsa-miR-146a-3p<br>hsa-miR-488-5p<br>hsa-miR-127-5p<br>hsa-miR-128-1-5p<br>hsa-miR-185-3p
#97	ASCRP3000965	0.004929519	0.20113116	1.5916847	up	hsa_circRNA_102855	hsa_circ_0057105	*PDK1*	hsa-miR-637<br>hsa-miR-362-5p<br>hsa-miR-500a-5p<br>hsa-miR-30a-5p<br>hsa-miR-511-5p
#98	ASCRP3007079	0.026109005	0.405761843	1.5914636	up	hsa_circRNA_002758	hsa_circ_0002758	*TSPAN15*	hsa-miR-6770-5p<br>hsa-miR-4459<br>hsa-miR-4428<br>hsa-miR-4717-5p<br>hsa-miR-505-5p

#99	ASCRP3003038	0.001851474	0.125885791	1.5899916	up	hsa_circRNA_000782	hsa_circ_0000230	*ZEB1*	hsa-miR-141-3p<br>hsa-miR-200a-3p<br>hsa-miR-148a-5p<br>hsa-miR-136-5p<br>hsa-miR-494-5p
#100	ASCRP3004096	0.005079415	0.203176598	1.5839757	up	hsa_circRNA_002994	hsa_circ_0002994	*ACLY*	hsa-miR-3160-5p<br>hsa-miR-6808-5p<br>hsa-miR-612<br>hsa-miR-4518<br>hsa-miR-1237-3p
#101	ASCRP3006837	0.007408692	0.234600286	1.5821509	up	hsa_circRNA_101006	hsa_circ_0025278	*TPI1*	hsa-miR-204-5p<br>hsa-miR-211-5p<br>hsa-miR-629-3p<br>hsa-miR-770-5p<br>hsa-miR-642a-5p
#102	ASCRP3002022	0.006316022	0.219733971	1.5801227	up	hsa_circRNA_102110	hsa_circ_0044301	*KPNB1*	hsa-miR-146a-3p<br>hsa-miR-488-5p<br>hsa-miR-127-5p<br>hsa-miR-128-1-5p<br>hsa-miR-185-3p
#103	ASCRP3001742	0.001136042	0.096473031	1.5793354	up	hsa_circRNA_100582	hsa_circ_0004126	*ZEB1*	hsa-miR-141-3p<br>hsa-miR-200a-3p<br>hsa-miR-148a-5p<br>hsa-miR-452-3p<br>hsa-miR-877-3p
#104	ASCRP3002882	0.00036148	0.052714578	1.5782827	up	hsa_circRNA_403876		*PDAP1*	hsa-miR-4778-3p<br>hsa-miR-1184<br>hsa-miR-942-5p<br>hsa-miR-3127-5p<br>hsa-miR-5047
#105	ASCRP3009267	0.036604648	0.45977598	1.5758009	up	hsa_circRNA_102854	hsa_circ_0057104	*PDK1*	hsa-miR-362-5p<br>hsa-miR-500a-5p<br>hsa-miR-30a-5p<br>hsa-miR-30e-5p<br>hsa-miR-21-3p
#106	ASCRP3008619	0.005009046	0.201222672	1.5751344	up	hsa_circRNA_104473	hsa_circ_0082149	*SND1*	hsa-miR-891a-3p<br>hsa-miR-518c-5p<br>hsa-miR-487a-5p<br>hsa-miR-593-3p<br>hsa-miR-335-5p
#107	ASCRP3006910	0.001209567	0.100125248	1.5722326	up	hsa_circRNA_079245	hsa_circ_0079245	*SLC29A4*	hsa-miR-6829-3p<br>hsa-miR-125a-5p<br>hsa-miR-212-5p<br>hsa-miR-125b-5p<br>hsa-miR-874-3p
#108	ASCRP3003341	0.000671603	0.070223719	1.5702978	up	hsa_circRNA_103850	hsa_circ_0003503	*ADAMTS6*	hsa-miR-589-3p<br>hsa-miR-363-3p<br>hsa-miR-370-5p<br>hsa-miR-23a-3p<br>hsa-miR-23b-3p
#109	ASCRP3008147	0.037551443	0.46577856	1.5639144	up	hsa_circRNA_102378	hsa_circ_0003423	*ZNF532*	hsa-miR-29b-1-5p<br>hsa-miR-216a-3p<br>hsa-miR-449c-5p<br>hsa-miR-597-3p<br>hsa-miR-589-5p

#110	ASCRP3009579	0.012428788	0.293796682	1.5635119	up	hsa_circRNA_104052	hsa_circ_0008285	*CDYL*	hsa-miR-892a<br>hsa-miR-378a-5p<br>hsa-miR-887-5p<br>hsa-miR-22-5p<br>hsa-miR-328-3p
#111	ASCRP3012994	0.002654649	0.152131811	1.5599467	up	hsa_circRNA_100023	hsa_circ_0009463	*NPHP4*	hsa-miR-766-3p<br>hsa-miR-149-3p<br>hsa-miR-593-5p<br>hsa-miR-608<br>hsa-miR-597-3p
#112	ASCRP3011224	0.01784402	0.344506807	1.5574985	up	hsa_circRNA_102108	hsa_circ_0002854	*KPNB1*	hsa-miR-23b-5p<br>hsa-miR-146a-3p<br>hsa-miR-488-5p<br>hsa-miR-127-5p<br>hsa-miR-299-3p
#113	ASCRP3004078	0.01078962	0.278108977	1.5551129	up	hsa_circRNA_103244	hsa_circ_0008806	*CCDC134*	hsa-miR-625-5p<br>hsa-miR-766-3p<br>hsa-miR-615-5p<br>hsa-miR-22-5p<br>hsa-miR-561-5p
#114	ASCRP3011115	0.000335509	0.051272723	1.5454752	up	hsa_circRNA_103628	hsa_circ_0008453	*APBB2*	hsa-miR-608<br>hsa-miR-647<br>hsa-miR-192-3p<br>hsa-miR-30c-2-3p<br>hsa-miR-19b-3p
#115	ASCRP3012005	0.033960462	0.448568817	1.5422406	up	hsa_circRNA_405234		*SAMD4A*	hsa-miR-6883-5p<br>hsa-miR-2277-5p<br>hsa-miR-4523<br>hsa-miR-6780b-5p<br>hsa-miR-6797-5p
#116	ASCRP3009530	0.004422403	0.187598036	1.542223	up	hsa_circRNA_070616	hsa_circ_0070616	*PAPSS1*	hsa-miR-4786-3p<br>hsa-miR-6841-3p<br>hsa-miR-642a-5p<br>hsa-miR-5704<br>hsa-miR-574-5p
#117	ASCRP3006359	0.027384562	0.414769983	1.5421477	up	hsa_circRNA_104438	hsa_circ_0004312	*AP4M1*	hsa-miR-637<br>hsa-miR-647<br>hsa-miR-485-5p<br>hsa-miR-628-5p<br>hsa-miR-93-3p
#118	ASCRP3009602	0.03525238	0.45177302	1.5390024	up	hsa_circRNA_027966	hsa_circ_0027966	*SLC41A2*	hsa-miR-6501-5p<br>hsa-miR-17-3p<br>hsa-miR-653-3p<br>hsa-miR-29b-1-5p<br>hsa-miR-4506
#119	ASCRP3001427	0.000524627	0.061309307	1.537427	up	hsa_circRNA_104851	hsa_circ_0008812	*RAD23B*	hsa-miR-138-5p<br>hsa-miR-325<br>hsa-miR-593-3p<br>hsa-miR-512-3p<br>hsa-miR-766-5p
#120	ASCRP3006930	0.014025672	0.306763318	1.5374225	up	hsa_circRNA_402533		*OVOL2*	hsa-miR-129-1-3p<br>hsa-miR-129-2-3p<br>hsa-miR-5008-5p<br>hsa-miR-1915-5p<br>hsa-miR-3158-5p

#121	ASCRP3005224	0.030150947	0.434827134	1.5345168	up	hsa_circRNA_102392	hsa_circ_0048009	*ATP9B*	hsa-miR-485-3p<br>hsa-miR-23b-5p<br>hsa-miR-888-3p<br>hsa-miR-1264<br>hsa-miR-764
#122	ASCRP3009528	0.011520685	0.286111338	1.5273129	up	hsa_circRNA_005280	hsa_circ_0005280	*STK39*	hsa-miR-4324<br>hsa-miR-6856-5p<br>hsa-miR-526b-5p<br>hsa-miR-1200<br>hsa-miR-7976
#123	ASCRP3006565	0.019990877	0.358661343	1.5239974	up	hsa_circRNA_100831	hsa_circ_0000311	*SDHAF2*	hsa-miR-215-3p<br>hsa-miR-376b-3p<br>hsa-miR-376a-3p<br>hsa-miR-450a-1-3p<br>hsa-miR-650
#124	ASCRP3005963	0.001148645	0.096473031	1.5235693	up	hsa_circRNA_104119	hsa_circ_0076691	*RUNX2*	hsa-miR-7-5p<br>hsa-miR-663a<br>hsa-miR-149-3p<br>hsa-miR-885-3p<br>hsa-miR-661
#125	ASCRP3000737	0.00617047	0.219733971	1.5218712	up	hsa_circRNA_092536	hsa_circ_0001382	*MFI2*	hsa-miR-4448<br>hsa-miR-432-3p<br>hsa-miR-4268<br>hsa-miR-376b-3p<br>hsa-miR-6511b-3p
#126	ASCRP3003779	0.005316818	0.210553044	1.5172398	up	hsa_circRNA_005613	hsa_circ_0005613	*RAPH1*	hsa-miR-93-3p<br>hsa-miR-22-5p<br>hsa-miR-4716-5p<br>hsa-miR-4663<br>hsa-miR-141-5p
#127	ASCRP3009671	0.021018884	0.364241968	1.5142582	up	hsa_circRNA_006226	hsa_circ_0006226	*PTPRG*	hsa-miR-548a-5p<br>hsa-miR-548i<br>hsa-miR-4668-3p<br>hsa-miR-548ab<br>hsa-miR-548ar-5p
#128	ASCRP3005875	0.001632309	0.1201057	1.513113	up	hsa_circRNA_104603	hsa_circ_0008015	*SLC20A2*	hsa-miR-214-3p<br>hsa-miR-195-5p<br>hsa-miR-16-5p<br>hsa-miR-671-5p<br>hsa-miR-15a-5p
#129	ASCRP3002729	0.046834747	0.510768692	1.5088319	up	hsa_circRNA_104104	hsa_circ_0076254	*BTBD9*	hsa-miR-17-3p<br>hsa-miR-15b-5p<br>hsa-miR-15a-5p<br>hsa-miR-424-5p<br>hsa-miR-190a-5p
#130	ASCRP3011039	0.003615838	0.174497099	1.5006023	up	hsa_circRNA_101314	hsa_circ_0003643	*HNRNPC*	hsa-miR-889-3p<br>hsa-miR-329-3p<br>hsa-miR-708-5p<br>hsa-miR-588<br>hsa-miR-490-3p
	**probeID**	**P-value**	**FDR**	**FC (abs)**	**Reg**	**circRNA**	**Alias**	**Gene Symbol**	**miRNA response elements**
#1	ASCRP3011969	2.37327E-05	0.018170386	0.1500853	down	hsa_circRNA_101491	hsa_circ_0034762	*MAPKBP1*	hsa-miR-17-3p<br>hsa-miR-125b-5p<br>hsa-miR-125a-5p

									hsa-miR-584-5p<br>hsa-miR-562
#2	ASCRP3002845	4.23324E-05	0.020419141	0.1784827	down	hsa_circRNA_405814		*ZNF470*	hsa-miR-4262<br>hsa-miR-181c-5p<br>hsa-miR-181b-5p<br>hsa-miR-181a-5p<br>hsa-miR-181d-5p
#3	ASCRP3013015	2.01679E-05	0.017171552	0.1948888	down	hsa_circRNA_023016	hsa_circ_0023016	*RBM4*	hsa-miR-3916<br>hsa-miR-765<br>hsa-miR-650<br>hsa-miR-6510-5p<br>hsa-miR-7106-5p
#4	ASCRP3010305	3.76801E-05	0.020419141	0.2269408	down	hsa_circRNA_001653	hsa_circ_0001568	*DUSP22*	hsa-miR-342-3p<br>hsa-let-7g-3p<br>hsa-miR-486-3p<br>hsa-miR-377-3p<br>hsa-miR-612
#5	ASCRP3007870	0.000596273	0.066207613	0.2282861	down	hsa_circRNA_101379	hsa_circ_0032363	*SMOC1*	hsa-miR-612<br>hsa-miR-194-3p<br>hsa-miR-542-5p<br>hsa-miR-432-5p<br>hsa-miR-339-5p
#6	ASCRP3011550	0.005616287	0.215854454	0.3077343	down	hsa_circRNA_082319	hsa_circ_0082319	*ZC3HC1*	hsa-miR-3692-5p<br>hsa-miR-149-5p<br>hsa-miR-939-5p<br>hsa-miR-6760-5p<br>hsa-miR-760
#7	ASCRP3002501	0.001986746	0.131566752	0.3150224	down	hsa_circRNA_003057	hsa_circ_0003057	*ANKH*	hsa-miR-134-5p<br>hsa-miR-943<br>hsa-miR-4474-3p<br>hsa-miR-5187-5p<br>hsa-miR-3118
#8	ASCRP3001122	0.012859228	0.299378892	0.3171076	down	hsa_circRNA_402458		*CCNYL1*	hsa-miR-1236-3p<br>hsa-miR-3064-5p<br>hsa-miR-6504-5p<br>hsa-miR-943<br>hsa-miR-522-3p
#9	ASCRP3002172	0.000109446	0.031215885	0.3279136	down	hsa_circRNA_092523	hsa_circ_0001255	*PANX2*	hsa-miR-2276-5p<br>hsa-miR-6865-3p<br>hsa-miR-7112-3p<br>hsa-miR-593-5p<br>hsa-miR-6767-3p
#10	ASCRP3010582	0.000243062	0.041989814	0.3465939	down	hsa_circRNA_406655		*CTC-228N24.3*	hsa-miR-212-5p<br>hsa-miR-4723-3p<br>hsa-miR-6764-3p<br>hsa-miR-548l<br>hsa-miR-136-5p
#11	ASCRP3007842	0.000937826	0.085991393	0.3567060	down	hsa_circRNA_405137			hsa-miR-4656<br>hsa-miR-5197-3p<br>hsa-miR-3160-3p<br>hsa-miR-4744<br>hsa-miR-6721-5p
#12	ASCRP3007536	1.59006E-05	0.015794596	0.3622664	down	hsa_circRNA_406698			hsa-miR-3916<br>hsa-miR-6873-5p<br>hsa-miR-4644

									hsa-miR-5584-5p<br>hsa-miR-1183
#13	ASCRP3007568	0.007375988	0.23445808	0.3693646	down	hsa_circRNA_087631	hsa_circ_0087631	*HABP4*	hsa-miR-4723-3p<br>hsa-miR-3183<br>hsa-miR-7111-3p<br>hsa-miR-346<br>hsa-miR-5001-3p
#14	ASCRP3005758	0.000427762	0.058608342	0.3740897	down	hsa_circRNA_103372	hsa_circ_0003748	*IP6K2*	hsa-miR-494-5p<br>hsa-miR-577<br>hsa-miR-628-5p<br>hsa-miR-647<br>hsa-miR-526b-5p
#15	ASCRP3011877	0.000602343	0.066207613	0.3752304	down	hsa_circRNA_101213	hsa_circ_0029426	*RAN*	hsa-miR-431-3p<br>hsa-miR-493-3p<br>hsa-miR-372-5p
#16	ASCRP3009813	0.00051171	0.061309307	0.3839756	down	hsa_circRNA_034642	hsa_circ_0034642	*VPS18*	hsa-miR-6836-5p<br>hsa-miR-6752-5p<br>hsa-miR-6791-3p<br>hsa-miR-1184<br>hsa-miR-4763-3p
#17	ASCRP3009369	0.000177312	0.036347001	0.3986713	down	hsa_circRNA_001654	hsa_circ_0001605	*CNPY3*	hsa-miR-665<br>hsa-miR-129-5p<br>hsa-miR-539-3p<br>hsa-miR-520a-5p<br>hsa-miR-30b-3p
#18	ASCRP3012939	0.000156077	0.034452544	0.4032329	down	hsa_circRNA_104670	hsa_circ_0001818	*UBR5*	hsa-miR-17-3p<br>hsa-miR-433-3p<br>hsa-miR-367-5p<br>hsa-miR-335-3p<br>hsa-miR-642a-5p
#19	ASCRP3003019	0.000542827	0.062216365	0.4141731	down	hsa_circRNA_001490	hsa_circ_0001490	*KIF2A*	hsa-miR-659-3p<br>hsa-miR-5002-5p<br>hsa-miR-203b-5p<br>hsa-miR-4460<br>hsa-miR-6718-5p
#20	ASCRP3011719	0.000110563	0.031215885	0.4207323	down	hsa_circRNA_104310	hsa_circ_0079385	*ZDHHC4*	hsa-miR-499a-3p<br>hsa-miR-1271-3p<br>hsa-miR-216a-3p<br>hsa-miR-197-3p<br>hsa-miR-489-3p
#21	ASCRP3009988	0.014418745	0.309678281	0.4276269	down	hsa_circRNA_010027	hsa_circ_0010027	*PDPN*	hsa-miR-4315<br>hsa-miR-8058<br>hsa-miR-4254<br>hsa-miR-6765-3p<br>hsa-miR-221-5p
#22	ASCRP3008204	0.000190596	0.036347001	0.4330710	down	hsa_circRNA_400027	hsa_circ_0092367	*SNURF-SNRPN*	hsa-miR-324-5p<br>hsa-miR-561-5p<br>hsa-miR-671-5p<br>hsa-miR-613<br>hsa-miR-422a
#23	ASCRP3012086	0.010450464	0.27317879	0.4367439	down	hsa_circRNA_100632	hsa_circ_0018905	*SAMD8*	hsa-miR-505-5p<br>hsa-miR-16-1-3p<br>hsa-miR-636<br>hsa-miR-141-5p<br>hsa-miR-626

#24	ASCRP3010339	0.010332009	0.271578899	0.4465328	down	hsa_circRNA_101380	hsa_circ_0032368	*SMOC1*	hsa-miR-194-3p<br>hsa-miR-34b-5p<br>hsa-miR-199a-3p<br>hsa-miR-199b-3p<br>hsa-miR-612
#25	ASCRP3010469	0.000458935	0.060115425	0.4482451	down	hsa_circRNA_006773	hsa_circ_0006773	*HIBADH*	hsa-miR-548av-3p<br>hsa-miR-4716-5p<br>hsa-miR-510-3p<br>hsa-miR-302b-3p<br>hsa-miR-548ar-3p
#26	ASCRP3009275	0.008535841	0.246958447	0.4591004	down	hsa_circRNA_102213	hsa_circ_0045932	*USP36*	hsa-miR-544a<br>hsa-miR-873-5p<br>hsa-miR-25-5p<br>hsa-miR-152-5p<br>hsa-miR-506-5p
#27	ASCRP3005757	0.002445582	0.142201625	0.4605513	down	hsa_circRNA_404935		*ZBTB16*	hsa-miR-6764-5p<br>hsa-miR-1915-3p<br>hsa-miR-760<br>hsa-miR-3124-3p<br>hsa-miR-6741-3p
#28	ASCRP3002158	2.43898E-05	0.018170386	0.4631837	down	hsa_circRNA_033572	hsa_circ_0033572	*AHNAK2*	hsa-miR-612<br>hsa-miR-661<br>hsa-miR-939-3p<br>hsa-miR-365b-5p<br>hsa-miR-6774-5p
#29	ASCRP3006170	1.42158E-05	0.015778114	0.4699187	down	hsa_circRNA_101522	hsa_circ_0035277	*DMXL2*	hsa-miR-224-5p<br>hsa-miR-616-3p<br>hsa-miR-520g-5p<br>hsa-miR-576-3p<br>hsa-miR-708-3p
#30	ASCRP3013256	0.003010784	0.161660111	0.4706268	down	hsa_circRNA_085287	hsa_circ_0085287	*ATP6V1C1*	hsa-miR-5189-3p<br>hsa-miR-8068<br>hsa-miR-629-3p<br>hsa-miR-5195-3p<br>hsa-miR-4526
#31	ASCRP3009819	0.000573983	0.064545961	0.4765436	down	hsa_circRNA_006169	hsa_circ_0006169	*HNRNPA3P6*	hsa-miR-4715-3p<br>hsa-miR-6501-5p<br>hsa-miR-744-3p<br>hsa-miR-934<br>hsa-miR-598-3p
#32	ASCRP3003410	0.012333739	0.293796682	0.4821748	down	hsa_circRNA_007352	hsa_circ_0007352	*AKAP17A*	hsa-miR-2682-3p<br>hsa-miR-6836-5p<br>hsa-miR-6132<br>hsa-miR-6882-3p<br>hsa-miR-6739-3p
#33	ASCRP3008559	0.000192102	0.036347001	0.4864121	down	hsa_circRNA_000742	hsa_circ_0000742	*RP11-599B13.6*	hsa-miR-1247-3p<br>hsa-miR-7110-3p<br>hsa-miR-6799-3p<br>hsa-miR-6814-3p<br>hsa-miR-3173-5p
#34	ASCRP3009644	0.000105097	0.031215885	0.4914630	down	hsa_circRNA_001409	hsa_circ_0001204	*DGCR8*	hsa-miR-612<br>hsa-miR-657<br>hsa-miR-362-3p<br>hsa-miR-377-3p<br>hsa-miR-136-5p

#35	ASCRP3003659	0.018741221	0.351230204	0.5068439	down	hsa_circRNA_052372	hsa_circ_0052372	*TRIM28*	hsa-miR-1256<br>hsa-miR-3936<br>hsa-miR-125a-3p<br>hsa-miR-4498<br>hsa-miR-3665
#36	ASCRP3013327	0.00392484	0.179735849	0.5087105	down	hsa_circRNA_104193	hsa_circ_0077930	*AHI1*	hsa-miR-335-3p<br>hsa-miR-587<br>hsa-miR-1185-2-3p<br>hsa-miR-105-5p<br>hsa-miR-1185-1-3p
#37	ASCRP3007654	9.71392E-05	0.031215885	0.5105025	down	hsa_circRNA_400033	hsa_circ_0092360	*RPL23A*	hsa-miR-509-5p<br>hsa-miR-497-3p<br>hsa-miR-328-3p<br>hsa-miR-764<br>hsa-miR-512-5p
#38	ASCRP3000513	0.000519998	0.061309307	0.5121624	down	hsa_circRNA_103361	hsa_circ_0001296	*SMARCC1*	hsa-miR-449c-3p<br>hsa-miR-582-5p<br>hsa-miR-509-3p<br>hsa-miR-510-5p<br>hsa-miR-369-5p
#39	ASCRP3001732	0.002124286	0.136323166	0.5173450	down	hsa_circRNA_000479	hsa_circ_0000479	*EPSTI1*	hsa-miR-942-5p<br>hsa-miR-4753-3p<br>hsa-miR-6873-3p<br>hsa-miR-6739-3p<br>hsa-miR-6809-3p
#40	ASCRP3013396	0.001458667	0.113812049	0.5285033	down	hsa_circRNA_403517		*SQSTM1*	hsa-miR-660-5p<br>hsa-miR-5189-5p<br>hsa-miR-3187-5p<br>hsa-miR-151a-5p<br>hsa-miR-4664-5p
#41	ASCRP3004844	4.92131E-05	0.021904876	0.5300852	down	hsa_circRNA_405040		*PLXNC1*	hsa-miR-6507-5p<br>hsa-miR-5571-5p<br>hsa-miR-3927-3p<br>hsa-miR-3190-5p<br>hsa-miR-4529-5p
#42	ASCRP3004672	0.00622687	0.219733971	0.5303096	down	hsa_circRNA_003416	hsa_circ_0003416	*TMSB4X*	hsa-miR-1244<br>hsa-miR-4747-5p<br>hsa-miR-6755-3p<br>hsa-miR-548an<br>hsa-miR-3194-5p
#43	ASCRP3006528	0.007474084	0.23506882	0.5305689	down	hsa_circRNA_101969	hsa_circ_0041821	*NEURL4*	hsa-miR-18a-3p<br>hsa-miR-519a-5p<br>hsa-miR-522-5p<br>hsa-miR-519c-5p<br>hsa-miR-518e-5p
#44	ASCRP3008107	0.043726252	0.494982834	0.5386546	down	hsa_circRNA_055243	hsa_circ_0055243	*ALMS1*	hsa-miR-3192-5p<br>hsa-miR-130b-5p<br>hsa-miR-6876-3p<br>hsa-miR-1272<br>hsa-miR-6868-3p
#45	ASCRP3011446	0.000996744	0.089332257	0.5410147	down	hsa_circRNA_401041		*ATP2A2*	hsa-miR-4517<br>hsa-miR-130b-5p<br>hsa-miR-4743-3p<br>hsa-miR-4769-3p<br>hsa-miR-4722-3p

#46	ASCRP3010255	0.000393042	0.055633194	0.5411036	down	hsa_circRNA_103852	hsa_circ_0072665	*ADAMTS6*	hsa-miR-766-3p<br>hsa-miR-136-5p<br>hsa-miR-373-3p<br>hsa-miR-302c-3p<br>hsa-miR-138-5p
#47	ASCRP3013460	0.004020286	0.180435077	0.5499883	down	hsa_circRNA_100323	hsa_circ_0013958	*ACP6*	hsa-miR-545-3p<br>hsa-miR-134-5p<br>hsa-miR-660-3p<br>hsa-miR-509-3p<br>hsa-miR-629-3p
#48	ASCRP3004356	0.001609064	0.119329367	0.5556014	down	hsa_circRNA_006718	hsa_circ_0006718	*CWF19L1*	hsa-miR-181d-5p<br>hsa-miR-4427<br>hsa-miR-181b-5p<br>hsa-miR-3941<br>hsa-miR-892c-3p
#49	ASCRP3006087	0.000616531	0.066207613	0.5622419	down	hsa_circRNA_101282	hsa_circ_0030569	*ABCC4*	hsa-miR-30d-3p<br>hsa-miR-29a-5p<br>hsa-miR-584-3p<br>hsa-miR-30a-3p<br>hsa-miR-30e-3p
#50	ASCRP3009639	0.004041625	0.180435077	0.5642502	down	hsa_circRNA_101093	hsa_circ_0027470	*NUP107*	hsa-miR-892b<br>hsa-miR-335-3p<br>hsa-miR-505-5p<br>hsa-miR-520f-3p<br>hsa-miR-320b
#51	ASCRP3008557	0.002856554	0.157534825	0.5647120	down	hsa_circRNA_405421		*MCTP2*	hsa-miR-892b<br>hsa-miR-4778-3p<br>hsa-miR-4676-3p<br>hsa-miR-4494<br>hsa-miR-4768-3p
#52	ASCRP3006839	1.40887E-05	0.015778114	0.5667619	down	hsa_circRNA_002086	hsa_circ_0001693	*LOC401320*	hsa-miR-153-5p<br>hsa-miR-185-3p<br>hsa-miR-199a-5p<br>hsa-miR-627-3p<br>hsa-miR-485-5p
#53	ASCRP3010410	0.000176093	0.036347001	0.5668094	down	hsa_circRNA_000618	hsa_circ_0000708	*FAM65A*	hsa-miR-769-5p<br>hsa-miR-1224-3p<br>hsa-miR-449b-3p<br>hsa-miR-497-3p<br>hsa-miR-885-5p
#54	ASCRP3009877	0.00305907	0.162847264	0.5669598	down	hsa_circRNA_000921	hsa_circ_0001120	*SNED1*	hsa-miR-183-5p<br>hsa-miR-221-5p<br>hsa-miR-202-5p<br>hsa-miR-328-3p<br>hsa-miR-331-3p
#55	ASCRP3011056	0.002415393	0.141134705	0.5764779	down	hsa_circRNA_104484	hsa_circ_0082326	*ZC3HC1*	hsa-miR-34b-5p<br>hsa-miR-508-3p<br>hsa-miR-378a-3p<br>hsa-miR-378d<br>hsa-miR-30c-2-3p
#56	ASCRP3006502	0.002984365	0.160966674	0.5802392	down	hsa_circRNA_062035	hsa_circ_0062035	*COL6A1*	hsa-miR-5001-5p<br>hsa-miR-4685-5p<br>hsa-miR-661<br>hsa-miR-4739<br>hsa-miR-6089

#57	ASCRP3010303	0.001035924	0.091468236	0.5855551	down	hsa_circRNA_001397	hsa_circ_0001397	*ZCCHC4*	hsa-miR-5095<br>hsa-miR-3929<br>hsa-miR-6884-5p<br>hsa-miR-4478<br>hsa-miR-4419b
#58	ASCRP3009307	0.014859569	0.315889579	0.5960431	down	hsa_circRNA_005008	hsa_circ_0005008	*HNRNPA1P48*	hsa-miR-4691-5p<br>hsa-miR-6792-3p<br>hsa-miR-181a-2-3p<br>hsa-miR-653-3p<br>hsa-miR-4717-5p
#59	ASCRP3012226	0.000875666	0.081546406	0.6037654	down	hsa_circRNA_069774	hsa_circ_0069774	*TMEM165*	hsa-miR-6739-5p<br>hsa-miR-4739<br>hsa-miR-2054<br>hsa-miR-5787<br>hsa-miR-3192-5p
#60	ASCRP3007208	0.00578642	0.217584002	0.6086586	down	hsa_circRNA_100512	hsa_circ_0005090	*SMYD3*	hsa-miR-182-5p<br>hsa-miR-181a-5p<br>hsa-miR-532-3p<br>hsa-miR-1224-3p<br>hsa-miR-181b-5p
#61	ASCRP3003196	0.003838068	0.178014667	0.6086991	down	hsa_circRNA_401033		*CRY1*	hsa-miR-7109-3p<br>hsa-miR-5584-5p<br>hsa-miR-6807-3p<br>hsa-miR-26b-3p<br>hsa-miR-1272
#62	ASCRP3013228	0.002851139	0.157534825	0.6099294	down	hsa_circRNA_034093	hsa_circ_0034093	*NIPA1*	hsa-miR-599<br>hsa-miR-548s<br>hsa-miR-421<br>hsa-miR-3194-5p<br>hsa-miR-3972
#63	ASCRP3004258	0.000484763	0.061309307	0.6104002	down	hsa_circRNA_104739	hsa_circ_0086419	*BNC2*	hsa-miR-29a-5p<br>hsa-miR-214-5p<br>hsa-miR-652-5p<br>hsa-miR-431-5p<br>hsa-miR-100-3p
#64	ASCRP3004767	0.003903084	0.17963226	0.6120192	down	hsa_circRNA_100616	hsa_circ_0004606	*ADAMTS14*	hsa-miR-370-3p<br>hsa-miR-657<br>hsa-miR-152-5p<br>hsa-miR-197-3p<br>hsa-miR-15b-5p
#65	ASCRP3007900	3.67954E-05	0.020419141	0.6150648	down	hsa_circRNA_102838	hsa_circ_0056856	*ITGB6*	hsa-miR-1301-3p<br>hsa-miR-140-3p<br>hsa-miR-93-3p<br>hsa-miR-19b-3p<br>hsa-miR-187-5p
#66	ASCRP3013614	0.000108472	0.031215885	0.6166597	down	hsa_circRNA_404567		*PHTF1*	hsa-miR-7703<br>hsa-miR-25-3p<br>hsa-miR-338-3p<br>hsa-miR-296-5p<br>hsa-miR-1304-5p
#67	ASCRP3000548	0.011826362	0.290062212	0.6183655	down	hsa_circRNA_104084	hsa_circ_0075829	*CASC15*	hsa-miR-506-3p<br>hsa-miR-644a<br>hsa-miR-605-3p<br>hsa-miR-124-3p<br>hsa-miR-1224-5p

#68	ASCRP3007563	0.000204154	0.036800403	0.6192699	down	hsa_circRNA_001350	hsa_circ_0000253	*BLNK*	hsa-miR-141-5p<br>hsa-miR-363-3p<br>hsa-miR-92b-3p<br>hsa-miR-92a-3p<br>hsa-miR-25-3p
#69	ASCRP3010445	0.000444366	0.059515119	0.6215727	down	hsa_circRNA_000881	hsa_circ_0000788	*MSI2*	hsa-miR-557<br>hsa-miR-423-3p<br>hsa-miR-323a-5p<br>hsa-miR-541-3p<br>hsa-miR-105-5p
#70	ASCRP3010200	0.024263067	0.391360976	0.6250964	down	hsa_circRNA_405443		*NDE1*	hsa-miR-7109-5p<br>hsa-miR-6780b-5p<br>hsa-miR-548k<br>hsa-miR-6761-5p<br>hsa-miR-4653-5p
#71	ASCRP3007526	0.003247123	0.167106571	0.6257153	down	hsa_circRNA_001714	hsa_circ_0001714	*BAZ1B*	hsa-miR-877-3p<br>hsa-miR-6809-3p<br>hsa-miR-6875-3p<br>hsa-miR-6515-3p<br>hsa-miR-6870-3p
#72	ASCRP3007287	0.003060217	0.162847264	0.6258661	down	hsa_circRNA_006296	hsa_circ_0006296	*CHD1L*	hsa-miR-6834-5p<br>hsa-miR-874-5p<br>hsa-miR-212-5p<br>hsa-miR-4267<br>hsa-miR-1226-5p
#73	ASCRP3008295	0.012096956	0.29189416	0.6263406	down	hsa_circRNA_103914	hsa_circ_0073378	*MCTP1*	hsa-miR-877-3p<br>hsa-miR-103a-3p<br>hsa-miR-107<br>hsa-miR-634<br>hsa-miR-29b-3p
#74	ASCRP3009532	7.70513E-05	0.030615042	0.6281989	down	hsa_circRNA_405261		*YLPM1*	hsa-miR-6507-5p<br>hsa-miR-548ba<br>hsa-miR-548ag<br>hsa-miR-561-3p<br>hsa-miR-570-5p
#75	ASCRP3012926	3.73383E-05	0.020419141	0.6300315	down	hsa_circRNA_003605	hsa_circ_0003605	*ZMIZ1*	hsa-miR-6829-5p<br>hsa-miR-6762-3p<br>hsa-miR-1273e<br>hsa-miR-4530<br>hsa-miR-6833-5p
#76	ASCRP3000833	0.049246525	0.523189462	0.6319175	down	hsa_circRNA_072654	hsa_circ_0072654	*CWC27*	hsa-miR-4778-3p<br>hsa-miR-6885-3p<br>hsa-miR-5691<br>hsa-miR-6805-3p<br>hsa-miR-6809-3p
#77	ASCRP3010338	0.005350258	0.210791919	0.6319221	down	hsa_circRNA_102171	hsa_circ_0004789	*SMURF2*	hsa-miR-539-5p<br>hsa-let-7f-2-3p<br>hsa-miR-216a-3p<br>hsa-miR-578<br>hsa-miR-587
#78	ASCRP3012148	0.002195466	0.137736578	0.6325781	down	hsa_circRNA_005420	hsa_circ_0005420	*ZC3H7A*	hsa-miR-548av-3p<br>hsa-miR-1279<br>hsa-miR-6876-3p<br>hsa-miR-4742-5p<br>hsa-miR-660-3p

#79	ASCRP3008231	0.011029053	0.282723243	0.6357313	down	hsa_circRNA_100509	hsa_circ_0017286	*SMYD3*	hsa-miR-516b-3p<br>hsa-miR-516a-3p<br>hsa-miR-671-5p<br>hsa-miR-425-3p<br>hsa-miR-584-5p
#80	ASCRP3007867	0.000112567	0.031215885	0.6418315	down	hsa_circRNA_104738	hsa_circ_0008732	*BNC2*	hsa-miR-655-5p<br>hsa-miR-19b-1-5p<br>hsa-miR-19b-2-5p<br>hsa-miR-877-3p<br>hsa-miR-130b-5p
#81	ASCRP3012915	0.000708214	0.072775074	0.6423892	down	hsa_circRNA_103444	hsa_circ_0008797	*GSK3B*	hsa-miR-508-5p<br>hsa-miR-500a-5p<br>hsa-miR-7-5p<br>hsa-miR-29a-5p<br>hsa-miR-623
#82	ASCRP3011918	0.002782619	0.155722159	0.6441121	down	hsa_circRNA_000314	hsa_circ_0000314		hsa-miR-6784-3p<br>hsa-miR-891a-3p<br>hsa-miR-6750-3p<br>hsa-miR-1224-3p<br>hsa-miR-6777-3p
#83	ASCRP3007143	0.007315641	0.233786714	0.6453628	down	hsa_circRNA_406157		*AGPAT3*	hsa-miR-4739<br>hsa-miR-1273h-5p<br>hsa-miR-5787<br>hsa-miR-1273g-3p<br>hsa-miR-4656
#84	ASCRP3007487	0.000536444	0.062081618	0.6510311	down	hsa_circRNA_000407	hsa_circ_0000407	*SMARCC2*	hsa-miR-197-3p<br>hsa-miR-4639-3p<br>hsa-miR-5001-3p<br>hsa-miR-7110-3p<br>hsa-miR-5587-3p
#85	ASCRP3011773	0.005649882	0.215854454	0.6517090	down	hsa_circRNA_066596	hsa_circ_0066596	*EPHA3*	hsa-miR-4700-3p<br>hsa-miR-4731-3p<br>hsa-miR-1537-5p<br>hsa-miR-1225-5p<br>hsa-miR-103b
#86	ASCRP3007421	0.004161194	0.181639508	0.6520275	down	hsa_circRNA_000435	hsa_circ_0000435	*C12orf75*	hsa-miR-593-3p<br>hsa-miR-484<br>hsa-miR-6762-3p<br>hsa-miR-6804-3p<br>hsa-miR-203a-5p
#87	ASCRP3002476	0.000139579	0.033621593	0.6527286	down	hsa_circRNA_407205		*RP11-535M15.1*	hsa-miR-6791-5p<br>hsa-miR-4685-3p<br>hsa-miR-942-5p<br>hsa-miR-4292<br>hsa-miR-345-5p
#88	ASCRP3002996	0.001687923	0.122009484	0.6529650	down	hsa_circRNA_100748	hsa_circ_0020926	*STIM1*	hsa-miR-136-3p<br>hsa-miR-598-3p<br>hsa-miR-556-3p<br>hsa-miR-335-3p<br>hsa-miR-499a-3p
#89	ASCRP3006013	0.003248887	0.167106571	0.6532234	down	hsa_circRNA_100511	hsa_circ_0017289	*SMYD3*	hsa-miR-532-3p<br>hsa-miR-516a-3p<br>hsa-miR-516b-3p<br>hsa-miR-182-5p<br>hsa-miR-181a-5p

#90	ASCRP3007522	0.011671007	0.288523833	0.6535447	down	hsa_circRNA_101847	hsa_circ_0040000	*TANGO6*	hsa-miR-146a-3p<br>hsa-miR-708-5p<br>hsa-miR-141-3p<br>hsa-miR-370-5p<br>hsa-miR-200a-3p
#91	ASCRP3013164	0.000112608	0.031215885	0.6539596	down	hsa_circRNA_083999	hsa_circ_0083999	*FGFR1*	hsa-miR-6769a-5p<br>hsa-miR-6769b-5p<br>hsa-miR-3692-3p<br>hsa-miR-130a-5p<br>hsa-miR-1226-5p
#92	ASCRP3008127	0.001470391	0.113812049	0.6564251	down	hsa_circRNA_101373	hsa_circ_0005139	*RDH11*	hsa-miR-33a-5p<br>hsa-miR-20a-3p<br>hsa-miR-9-5p<br>hsa-miR-642a-3p<br>hsa-miR-33b-5p
#93	ASCRP3006695	0.005935278	0.218417816	0.6566900	down	hsa_circRNA_005198	hsa_circ_0005198	*PARP4*	hsa-miR-4459<br>hsa-miR-450b-5p<br>hsa-miR-4254<br>hsa-miR-4778-3p<br>hsa-miR-1237-3p
#94	ASCRP3004460	0.006102266	0.219733971	0.6580963	down	hsa_circRNA_022917	hsa_circ_0022917	*EFEMP2*	hsa-miR-4632-5p<br>hsa-miR-6893-5p<br>hsa-miR-505-5p<br>hsa-miR-6879-5p<br>hsa-miR-6721-5p
#95	ASCRP3007280	0.002334392	0.140535116	0.6589467	down	hsa_circRNA_027446	hsa_circ_0027446	*HMGA2*	hsa-miR-129-5p<br>hsa-miR-331-3p<br>hsa-miR-6882-3p<br>hsa-miR-3925-3p<br>hsa-miR-1236-3p
#96	ASCRP3004372	0.008188672	0.244124552	0.6595081	down	hsa_circRNA_402031		*ANGPTL4*	hsa-miR-4776-3p<br>hsa-miR-143-5p<br>hsa-miR-4685-5p<br>hsa-miR-500b-3p<br>hsa-miR-2355-3p
#97	ASCRP3005052	0.002076144	0.135233004	0.6664074	down	hsa_circRNA_084010	hsa_circ_0084010	*FGFR1*	hsa-miR-759<br>hsa-miR-298<br>hsa-miR-4435<br>hsa-miR-92b-5p<br>hsa-miR-490-5p

Table S5 – Differentially expressed miRNA in osteogenic differentiation.

Mature_ID	Pre_ID	Fold_Change	p_value	q_value	Day_7_Osteo - avg log2(CPM)	Day_7_CRL_monolayer - avg log2(CPM)
hsa-miR-5690	hsa-mir-5690	7.24955252	4.36979E-10	6.31872E-08	0.746389457	-2.11150249
hsa-miR-7704	hsa-mir-7704	3.185907327	0.002342098	0.036223051	0.894804642	-0.77689966

hsa-miR-4746-5p	hsa-mir-4746	2.684662606	0.009250521	0.098354803	0.38876923	-1.03597156
hsa-miR-199b-5p	hsa-mir-199b	2.655078161	0.002482677	0.037395316	10.60358228	9.194827948
hsa-miR-33b-3p	hsa-mir-33b	2.50410592	2.14544E-05	0.001410137	1.584791088	0.2604955
hsa-miR-6724-5p	hsa-mir-6724-1	2.419958005	0.010395162	0.103616164	0.070771897	-1.204210115
hsa-miR-6724-5p	hsa-mir-6724-2	2.419958005	0.010395162	0.103616164	0.070771897	-1.204210115
hsa-miR-6724-5p	hsa-mir-6724-3	2.419958005	0.010395162	0.103616164	0.070771897	-1.204210115
hsa-miR-6724-5p	hsa-mir-6724-4	2.419958005	0.010395162	0.103616164	0.070771897	-1.204210115
hsa-miR-1908-5p	hsa-mir-1908	2.384549993	0.000361658	0.010056883	1.3701895	0.116472471
hsa-miR-1275	hsa-mir-1275	2.153867057	0.01511122	0.128534258	1.022530901	-0.084398304
hsa-miR-3130-5p	hsa-mir-3130-2	2.034622042	0.022890153	0.174769164	0.075196674	-0.949564145
hsa-miR-3173-5p	hsa-mir-3173	1.995583172	0.006213005	0.074717877	1.707453528	0.710643119
hsa-miR-550a-3-5p	hsa-mir-550a-1	1.948254192	0.001024302	0.023142822	2.160016965	1.197835045
hsa-miR-550a-3-5p	hsa-mir-550a-2	1.948254192	0.001024302	0.023142822	2.160016965	1.197835045
hsa-miR-12135	hsa-mir-12135	1.941759956	0.003532656	0.049117512	3.452310924	2.494946061
hsa-miR-550a-5p	hsa-mir-550a-1	1.930070223	0.00126638	0.024745741	2.172623027	1.223969688
hsa-miR-550a-5p	hsa-mir-550a-2	1.930070223	0.00126638	0.024745741	2.172623027	1.223969688
hsa-miR-4421	hsa-mir-4421	1.905580044	0.023347395	0.175835066	0.449721325	-0.480508885
hsa-miR-3129-3p	hsa-mir-3129	1.904777516	3.87388E-05	0.00175051	3.19394816	2.264325663
hsa-miR-548az-5p	hsa-mir-548az	1.86382623	0.009224979	0.098354803	0.693159049	-0.205108311
hsa-miR-195-3p	hsa-mir-195	1.83663254	0.017601428	0.141398134	4.90414012	4.027077108
hsa-miR-3129-5p	hsa-mir-3129	1.797420558	0.001131625	0.024745741	2.595283069	1.749355061
hsa-miR-618	hsa-mir-618	1.769850458	0.000558156	0.013915399	5.386207396	4.56257993
hsa-miR-548ah-3p	hsa-mir-548ah	1.737754899	0.040104923	0.233837573	1.548609068	0.751384455
hsa-miR-92a-1-5p	hsa-mir-92a-1	1.736983525	0.045549116	0.255286908	3.075246538	2.278662467
hsa-miR-548au-5p	hsa-mir-548au	1.734410543	0.024343625	0.179596335	0.550942014	-0.243503418
hsa-miR-548av-3p	hsa-mir-548ah	1.730392617	0.047282051	0.25897669	1.52518817	0.734088755
hsa-miR-548av-3p	hsa-mir-548p	1.730392617	0.047282051	0.25897669	1.52518817	0.734088755
hsa-miR-652-5p	hsa-mir-652	1.656272161	0.019487204	0.153144003	1.589793133	0.861853375
hsa-miR-30a-3p	hsa-mir-30a	1.647588766	2.66131E-05	0.001480099	9.920437854	9.20008166

hsa-miR-7-5p	hsa-mir-7-1	1.646242568	0.001419466	0.025907101	10.85079215	10.13161522
hsa-miR-548am-3p	hsa-mir-548am	1.644735316	0.030211955	0.21003119	3.792914757	3.075059325
hsa-miR-7-5p	hsa-mir-7-3	1.641604019	0.001484153	0.025907101	10.83095395	10.11584778
hsa-miR-7-5p	hsa-mir-7-2	1.639532313	0.001486642	0.025907101	10.83767096	10.12438663
hsa-miR-548av-3p	hsa-mir-548o-2	1.631058877	0.016261283	0.134885426	5.348847417	4.643038557
hsa-miR-548o-3p	hsa-mir-548o-2	1.631058877	0.016261283	0.134885426	5.348847417	4.643038557
hsa-miR-4659a-3p	hsa-mir-4659a	1.626896908	0.049663713	0.259814355	0.174030541	-0.528092294
hsa-miR-548w	hsa-mir-548w	1.624986308	0.024809662	0.181185715	1.55445527	0.854027707
hsa-miR-1303	hsa-mir-1303	1.603468724	0.000484559	0.012512018	4.620403374	3.93920716
hsa-miR-942-5p	hsa-mir-942	1.588761341	0.012032518	0.115141689	2.943738127	2.275835704
hsa-miR-548aq-3p	hsa-mir-548h-3	1.577127082	0.01641759	0.134885426	2.027006417	1.369707503
hsa-miR-15b-3p	hsa-mir-15b	1.556578993	0.00797639	0.090108284	5.657381817	5.019003024
hsa-miR-3616-5p	hsa-mir-3616	1.546008631	0.044182922	0.251529547	1.010757649	0.382209275
hsa-miR-548aq-3p	hsa-mir-548aq	1.543303111	0.027987013	0.196452528	1.919882218	1.293860777
hsa-miR-30c-2-3p	hsa-mir-30c-2	1.517147231	0.00315942	0.044789431	6.419079424	5.817718326
hsa-let-7f-1-3p	hsa-let-7f-1	1.506573186	0.002354749	0.036223051	5.406223848	4.81495309
**Mature_ID**	**Pre_ID**	**Fold_Change**	**p_value**	**q_value**	**Day_7_Osteo - avg log2(CPM)**	**Day_7_CRL_monolayer - avg log2(CPM)**
hsa-miR-383-5p	hsa-mir-383	0.121428738	0.042928245	0.246326358	-1.279010889	1.762807304
hsa-miR-210-3p	hsa-mir-210	0.136514597	9.05421E-13	3.2731E-10	4.727892722	7.600765594
hsa-miR-9-5p	hsa-mir-9-2	0.149994571	0.033988065	0.217771254	5.105815765	7.842833572
hsa-miR-9-5p	hsa-mir-9-1	0.150007727	0.033993096	0.217771254	5.105815761	7.842707036
hsa-miR-9-5p	hsa-mir-9-3	0.150022397	0.033999439	0.217771254	5.105815759	7.842565955
hsa-miR-9-3p	hsa-mir-9-3	0.159479726	0.0359925	0.223909223	1.116750828	3.765305888
hsa-miR-9-3p	hsa-mir-9-1	0.16134191	0.037236638	0.223909223	1.128606078	3.760412931
hsa-miR-9-3p	hsa-mir-9-2	0.16134191	0.037236638	0.223909223	1.128606078	3.760412931
hsa-miR-210-5p	hsa-mir-210	0.184295331	1.70518E-06	0.00017612	0.068646785	2.508555355
hsa-miR-203a-3p	hsa-mir-203a	0.204603327	0.00768022	0.089125932	3.168228689	5.457327181
hsa-miR-129-1-3p	hsa-mir-129-1	0.261223963	0.026631583	0.192546346	-1.424887577	0.511753271
hsa-miR-335-5p	hsa-mir-335	0.265307392	1.29396E-22	9.35535E-20	5.082449209	6.996712434

hsa-miR-1297	hsa-mir-1297	0.278594835	0.033944025	0.217771254	-0.373395744	1.470363841
hsa-miR-122b-5p	hsa-mir-122b	0.291704032	1.79596E-05	0.001298482	-1.093770824	0.683651944
hsa-miR-31-3p	hsa-mir-31	0.294213687	1.9635E-06	0.000177452	3.956273326	5.721337056
hsa-miR-135b-5p	hsa-mir-135b	0.301838913	0.000306121	0.009221902	-0.469355543	1.258793742
hsa-miR-129-2-3p	hsa-mir-129-2	0.309293724	0.022648134	0.174769164	1.158370225	2.851320762
hsa-miR-200b-3p	hsa-mir-200b	0.332351348	0.023705494	0.176691465	4.026939679	5.61615857
hsa-miR-490-3p	hsa-mir-490	0.334171776	0.001504977	0.025907101	-0.79022674	0.791111464
hsa-miR-181c-5p	hsa-mir-181c	0.392693311	8.69593E-05	0.0028578	0.460208345	1.808733417
hsa-miR-181c-3p	hsa-mir-181c	0.398999739	3.57986E-06	0.000287582	2.346028818	3.671569112
hsa-miR-139-5p	hsa-mir-139	0.426258586	0.046171193	0.256782864	2.338236556	3.568435757
hsa-miR-335-3p	hsa-mir-335	0.426652103	7.90856E-11	1.90596E-08	8.742092061	9.970959998
hsa-miR-582-5p	hsa-mir-582	0.43803295	0.002202897	0.03539321	0.533094395	1.723983093
hsa-miR-224-3p	hsa-mir-224	0.440546341	0.001173689	0.024745741	2.345094411	3.527728721
hsa-miR-31-5p	hsa-mir-31	0.450182583	3.6179E-10	6.31872E-08	9.001074411	10.15249226
hsa-miR-542-3p	hsa-mir-542	0.455286102	4.31501E-08	5.19959E-06	4.824388609	5.959543282
hsa-miR-628-5p	hsa-mir-628	0.461969407	4.84429E-05	0.001941139	3.423971804	4.538102584
hsa-miR-585-3p	hsa-mir-585	0.464187052	0.047643433	0.258994002	2.806917848	3.914139661
hsa-miR-4497	hsa-mir-4497	0.465516137	0.003007731	0.043491793	0.966092837	2.069189752
hsa-miR-4683	hsa-mir-4683	0.474960834	0.012421925	0.115141689	-0.798834238	0.275285305
hsa-miR-616-3p	hsa-mir-616	0.488468571	0.013988776	0.123899262	-0.867394015	0.166268341
hsa-miR-4455	hsa-mir-4455	0.499434162	0.001420608	0.025907101	2.858799907	3.860433493
hsa-miR-424-5p	hsa-mir-424	0.507226843	2.46619E-05	0.001480099	4.896145781	5.87544278
hsa-let-7i-3p	hsa-let-7i	0.521642178	0.003692728	0.049852677	3.027581118	3.966448688
hsa-miR-217-5p	hsa-mir-217	0.531149337	0.010461936	0.103616164	0.588543058	1.501353609
hsa-miR-376a-2-5p	hsa-mir-376a-2	0.534038103	0.014598408	0.125650585	-0.492496348	0.412489067
hsa-miR-628-3p	hsa-mir-628	0.556373381	0.001241501	0.024745741	1.659296611	2.505171308
hsa-miR-664a-5p	hsa-mir-664a	0.558017064	0.013714582	0.123899262	1.883512578	2.725131433
hsa-let-7i-5p	hsa-let-7i	0.565135727	3.62724E-05	0.001748327	15.11791815	15.94124885
hsa-miR-582-3p	hsa-mir-582	0.565671764	0.006303998	0.074717877	2.662414256	3.484377192

hsa-miR-504-5p	hsa-mir-504	0.57395818	0.037669151	0.223909223	0.665789999	1.466772471
hsa-miR-452-5p	hsa-mir-452	0.584882841	0.005556542	0.069265172	7.782554052	8.556334481
hsa-miR-450a-2-3p	hsa-mir-450a-2	0.586213378	0.002970463	0.043491793	1.777741353	2.548243555
hsa-miR-143-5p	hsa-mir-143	0.591968512	4.20209E-05	0.001787125	8.952490558	9.708898216
hsa-miR-12136	hsa-mir-12136	0.595975038	0.012279751	0.115141689	3.040915556	3.787591746
hsa-miR-3200-3p	hsa-mir-3200	0.604271438	0.018069514	0.143563278	1.445536061	2.172267404
hsa-miR-328-3p	hsa-mir-328	0.604758352	3.2664E-05	0.00168686	6.957360645	7.682929951
hsa-miR-181d-5p	hsa-mir-181d	0.620227559	0.000952768	0.022961719	5.646070056	6.33520052
hsa-miR-450a-1-3p	hsa-mir-450a-1	0.625253778	0.012353529	0.115141689	0.903511964	1.580998188
hsa-miR-450a-5p	hsa-mir-450a-1	0.638075952	7.32504E-05	0.002521905	5.895566458	6.543766392
hsa-miR-1843	hsa-mir-1843	0.63812497	0.004380927	0.05758927	2.845201292	3.4932904
hsa-miR-450a-5p	hsa-mir-450a-2	0.638607031	7.22171E-05	0.002521905	5.899361623	6.546361282
hsa-miR-4521	hsa-mir-4521	0.649740125	0.009026509	0.098354803	7.457531997	8.079597289
hsa-miR-98-3p	hsa-mir-98	0.658037487	0.044679323	0.252368361	2.184849803	2.788608125
hsa-miR-15a-5p	hsa-mir-15a	0.660576783	0.004594207	0.059314492	3.910803017	4.509004847
hsa-miR-668-3p	hsa-mir-668	0.664439133	0.037135731	0.223909223	1.688391206	2.278182256

Table S6 – Differentially expressed miRNA in chondrogenic differentiation.

Mature_ID	Pre_ID	Fold_Change	p_value	q_value	Day_7_Chondro - avg log2(CPM)	Day_7_CRL_pellet - avg log2(CPM)
hsa-miR-3131	hsa-mir-3131	31.84911687	3.98357E-11	2.97882E-09	1.062890199	-3.930291265
hsa-miR-181b-3p	hsa-mir-181b-1	26.9962366	1.92153E-31	1.29319E-28	5.756585249	1.001898851
hsa-miR-675-5p	hsa-mir-675	24.65044939	1.29943E-09	5.46574E-08	1.371546641	-3.251995401
hsa-miR-181a-3p	hsa-mir-181a-1	20.56250572	8.74541E-28	2.94283E-25	9.74211181	5.380167635
hsa-miR-5690	hsa-mir-5690	20.38862738	4.18517E-08	1.22462E-06	0.415594092	-3.934098655
hsa-miR-140-3p	hsa-mir-140	19.79484789	3.07751E-25	6.90388E-23	13.83498883	9.527935758
hsa-miR-140-5p	hsa-mir-140	18.25034229	5.81955E-20	9.7914E-18	10.70041492	6.510563299
hsa-miR-675-3p	hsa-mir-675	12.61866767	6.57819E-07	1.34155E-05	0.888262736	-2.769224952
hsa-miR-7974	hsa-mir-7974	11.06304737	1.27969E-08	4.10109E-07	1.549858301	-1.917818631

hsa-miR-7973	hsa-mir-7973-2	8.998076518	3.90292E-05	0.000495598	0.357220217	-2.812396418
hsa-miR-95-3p	hsa-mir-95	7.776445328	0.001209953	0.007462192	2.648606478	-0.310504361
hsa-miR-181b-5p	hsa-mir-181b-2	6.486940124	2.90878E-13	3.01561E-11	11.55399912	8.856460993
hsa-miR-181b-5p	hsa-mir-181b-1	6.177897619	3.13659E-13	3.01561E-11	11.36622932	8.73911336
hsa-miR-449c-5p	hsa-mir-449c	4.813722066	4.91816E-10	2.54609E-08	2.397451295	0.130298448
hsa-miR-23b-5p	hsa-mir-23b	4.134856246	1.70311E-10	1.14619E-08	4.537879873	2.4900427
hsa-miR-4455	hsa-mir-4455	3.955469026	7.73037E-06	0.000115612	4.028602728	2.044753953
hsa-miR-27b-5p	hsa-mir-27b	3.936524668	1.1462E-12	9.64243E-11	6.51004221	4.533119691
hsa-miR-181a-5p	hsa-mir-181a-1	3.886138798	1.13787E-09	5.11667E-08	12.37383901	10.41550158
hsa-miR-181a-5p	hsa-mir-181a-2	3.886052006	1.14042E-09	5.11667E-08	12.37350137	10.41519617
hsa-miR-152-5p	hsa-mir-152	3.884748752	9.71807E-08	2.51549E-06	4.960462339	3.002641044
hsa-miR-760	hsa-mir-760	3.679689814	0.00828065	0.035049543	0.897827222	-0.981756935
hsa-miR-4521	hsa-mir-4521	3.599575612	3.46941E-10	1.94576E-08	8.919337759	7.071510935
hsa-miR-27b-3p	hsa-mir-27b	3.483302584	1.53601E-16	2.06747E-14	14.66307945	12.86262365
hsa-miR-3129-5p	hsa-mir-3129	3.358074622	7.12668E-07	1.41066E-05	2.492652124	0.745017835
hsa-miR-6716-3p	hsa-mir-6716	3.006662146	0.000398513	0.003155282	1.501641214	-0.086521549
hsa-miR-20a-5p	hsa-mir-20a	2.891875955	9.94903E-07	1.76203E-05	10.09396198	8.561956312
hsa-miR-1307-5p	hsa-mir-1307	2.852346597	1.04463E-07	2.60382E-06	3.317286699	1.8051374
hsa-miR-27a-5p	hsa-mir-27a	2.742297467	5.38497E-09	2.13182E-07	7.625401579	6.170016504
hsa-miR-17-5p	hsa-mir-17	2.721019763	3.15329E-08	9.64621E-07	9.257966775	7.81381934
hsa-miR-7-5p	hsa-mir-7-2	2.676376272	8.47972E-09	3.17047E-07	10.27728914	8.857008178
hsa-miR-7-5p	hsa-mir-7-3	2.670633775	1.05142E-08	3.72426E-07	10.26837596	8.851193809
hsa-miR-7-5p	hsa-mir-7-1	2.668922463	1.12688E-08	3.79196E-07	10.28359699	8.867339599
hsa-miR-301a-5p	hsa-mir-301a	2.588011824	0.000152272	0.001443371	1.909471059	0.53762685
hsa-miR-148a-5p	hsa-mir-148a	2.566513999	4.00115E-06	6.56775E-05	6.752075467	5.392265337
hsa-miR-16-1-3p	hsa-mir-16-1	2.521332694	0.008976164	0.037462149	2.110485922	0.776299425
hsa-miR-195-3p	hsa-mir-195	2.515846031	2.95104E-07	6.84846E-06	4.756541318	3.425497686
hsa-miR-877-5p	hsa-mir-877	2.43437927	5.71955E-05	0.000712826	2.888587171	1.605033217
hsa-miR-203a-3p	hsa-mir-203a	2.356186285	0.005777972	0.027578545	4.123615363	2.887161757

hsa-miR-21-3p	hsa-mir-21	2.286151009	0.000451983	0.003417803	7.720107142	6.52718644
hsa-miR-504-5p	hsa-mir-504	2.279070191	0.006138554	0.028889836	3.359514344	2.171068986
hsa-miR-92a-1-5p	hsa-mir-92a-1	2.25330037	0.000171083	0.001599154	3.45205133	2.28001169
hsa-miR-17-3p	hsa-mir-17	2.252035174	0.001102175	0.007064415	1.40442463	0.233195269
hsa-miR-18a-5p	hsa-mir-18a	2.237727221	0.005270805	0.025704725	2.371409413	1.20937523
hsa-miR-20b-5p	hsa-mir-20b	2.215805644	0.002004294	0.011056473	2.323290517	1.175459174
hsa-miR-9903	hsa-mir-9903	2.180647243	0.014121604	0.054619768	1.135516737	0.010760329
hsa-miR-199b-5p	hsa-mir-199b	2.148552684	3.68809E-07	8.27362E-06	12.05664563	10.95328048
hsa-miR-199a-5p	hsa-mir-199a-1	2.144464722	4.1445E-07	8.76531E-06	14.20565118	13.1050336
hsa-miR-199a-5p	hsa-mir-199a-2	2.144268174	4.16776E-07	8.76531E-06	14.20571581	13.10523046
hsa-miR-210-3p	hsa-mir-210	2.139524795	3.32512E-05	0.000430347	8.972919483	7.875629084
hsa-miR-26a-1-3p	hsa-mir-26a-1	2.135832853	0.023573858	0.080127305	0.823354641	-0.271444107
hsa-miR-651-5p	hsa-mir-651	2.122934419	0.000195527	0.001778238	2.982608166	1.896548361
hsa-miR-20a-3p	hsa-mir-20a	2.074729577	0.033277257	0.104667446	0.568436462	-0.484486844
hsa-miR-181c-3p	hsa-mir-181c	2.070594903	0.00042809	0.003279152	3.326205939	2.276160611
hsa-miR-143-3p	hsa-mir-143	2.062389989	1.03563E-05	0.000145205	14.95029697	13.90597981
hsa-miR-106a-5p	hsa-mir-106a	2.05749249	0.000285129	0.002369034	4.199227514	3.158340349
hsa-miR-181b-2-3p	hsa-mir-181b-2	2.049201677	0.018946706	0.068554479	0.502077865	-0.532984113
hsa-miR-143-5p	hsa-mir-143	2.029088129	0.000109237	0.001113887	8.391721017	7.37088949
hsa-miR-152-3p	hsa-mir-152	1.998831268	1.02707E-05	0.000145205	11.97619223	10.97703554
hsa-miR-1294	hsa-mir-1294	1.990723187	0.008171248	0.034805379	1.728681903	0.735389276
hsa-miR-548i	hsa-mir-548i-1	1.976262411	0.042445256	0.124198509	0.273815501	-0.708959022
hsa-miR-548i	hsa-mir-548i-2	1.976262411	0.042445256	0.124198509	0.273815501	-0.708959022
hsa-miR-548i	hsa-mir-548i-3	1.976262411	0.042445256	0.124198509	0.273815501	-0.708959022
hsa-miR-548i	hsa-mir-548i-4	1.976262411	0.042445256	0.124198509	0.273815501	-0.708959022
hsa-miR-1297	hsa-mir-1297	1.972373673	0.015667686	0.059572615	1.530543045	0.550610144
hsa-miR-23b-3p	hsa-mir-23b	1.96633967	5.08157E-06	8.1426E-05	11.37512268	10.39961012
hsa-miR-539-3p	hsa-mir-539	1.920745305	0.012404407	0.049397431	4.459288533	3.517622306
hsa-miR-652-5p	hsa-mir-652	1.917927429	0.009017635	0.037462149	1.174482173	0.234934041

hsa-miR-24-1-5p	hsa-mir-24-1	1.911147759	0.000242641	0.002101522	3.144580509	2.210141185
hsa-miR-191-3p	hsa-mir-191	1.885138197	0.007746749	0.033854299	1.656425537	0.741755247
hsa-miR-3616-5p	hsa-mir-3616	1.883102217	0.04173702	0.124198509	0.568083921	-0.345027393
hsa-miR-296-3p	hsa-mir-296	1.873881868	0.003289519	0.016899588	3.971428462	3.065398455
hsa-miR-548k	hsa-mir-548k	1.861393522	0.007356902	0.032986382	3.04706683	2.150683739
hsa-miR-548av-5p	hsa-mir-548k	1.856276738	0.008096962	0.034708634	3.042866255	2.150454449
hsa-miR-214-3p	hsa-mir-214	1.852842606	0.00027778	0.002336828	10.39961461	9.509874281
hsa-miR-145-5p	hsa-mir-145	1.831729803	0.004861744	0.023882873	10.70981121	9.8366045
hsa-miR-365a-5p	hsa-mir-365a	1.825539627	0.009840147	0.040380605	3.977970606	3.109647621
hsa-miR-181d-5p	hsa-mir-181d	1.82363282	0.003114264	0.016247285	6.155524072	5.288708793
hsa-miR-1285-3p	hsa-mir-1285-1	1.819312984	0.003466215	0.017539571	2.159173887	1.29578013
hsa-miR-148a-3p	hsa-mir-148a	1.802321663	0.000501595	0.003678027	14.83817022	13.98831371
hsa-miR-671-5p	hsa-mir-671	1.797190974	0.000631324	0.004472432	5.723683083	4.877939362
hsa-miR-149-5p	hsa-mir-149	1.789526739	0.001467324	0.008587034	5.598024542	4.758446441
hsa-miR-339-5p	hsa-mir-339	1.78027922	0.000484896	0.003625941	6.421845742	5.589742211
hsa-miR-497-5p	hsa-mir-497	1.776814839	0.001101189	0.007064415	4.517169579	3.687876233
hsa-miR-487a-3p	hsa-mir-487a	1.767996185	0.04575982	0.129942442	3.221557611	2.399442449
hsa-miR-145-3p	hsa-mir-145	1.739641023	0.003325453	0.016954773	9.643087892	8.844298257
hsa-miR-148b-3p	hsa-mir-148b	1.738233339	0.000229084	0.0020286	10.58917338	9.791551621
hsa-miR-32-5p	hsa-mir-32	1.736966852	0.021586622	0.074657161	4.124638042	3.32806782
hsa-miR-210-5p	hsa-mir-210	1.724130915	0.011885024	0.047610841	3.10048173	2.314612407
hsa-let-7f-2-3p	hsa-let-7f-2	1.7196814	0.002356365	0.012892959	4.366566819	3.584425513
hsa-miR-130b-3p	hsa-mir-130b	1.673764999	0.03328207	0.104667446	3.167113718	2.424016735
hsa-miR-3129-3p	hsa-mir-3129	1.656139635	0.024884998	0.083738018	2.836083042	2.108258725
hsa-miR-574-5p	hsa-mir-574	1.601790018	0.0042254	0.021064403	7.584225092	6.904540058
hsa-miR-30e-5p	hsa-mir-30e	1.557899116	0.003100865	0.016247285	8.644307903	8.004706091
hsa-miR-548e-3p	hsa-mir-548e	1.55157653	0.03846994	0.118365027	3.177334758	2.5435999
hsa-let-7a-3p	hsa-let-7a-3	1.535338145	0.001205632	0.007462192	8.283239046	7.664682615
hsa-let-7a-3p	hsa-let-7a-1	1.533912739	0.00122327	0.007462192	8.289602043	7.67238563

hsa-miR-92a-3p	hsa-mir-92a-2	1.522403282	0.000100534	0.00109502	11.86617586	11.25982528
hsa-miR-2277-5p	hsa-mir-2277	1.517402715	0.016638097	0.062555527	3.157616704	2.55601268
hsa-miR-340-5p	hsa-mir-340	1.512479413	0.02081268	0.073334731	7.489542707	6.892627202
hsa-miR-590-3p	hsa-mir-590	1.510884914	0.017178406	0.064228151	4.215092808	3.619699035
hsa-miR-195-5p	hsa-mir-195	1.510420884	0.042135653	0.124198509	6.239287055	5.644336437
hsa-miR-92a-3p	hsa-mir-92a-1	1.502547214	0.000174679	0.001610396	11.96462074	11.37721041
**Mature_ID**	**Pre_ID**	**Fold_Change**	**p_value**	**q_value**	**Day_7_Chondro - avg log2(CPM)**	**Day_7_CRL_pellet - avg log2(CPM)**
hsa-miR-873-3p	hsa-mir-873	0.030876724	6.92167E-08	1.94095E-06	-3.34787105	1.669465439
hsa-miR-144-3p	hsa-mir-144	0.087000139	1.88612E-05	0.000259053	-1.148385231	2.374453256
hsa-miR-451a	hsa-mir-451a	0.092468296	1.0254E-05	0.000145205	5.794669531	9.229566922
hsa-miR-144-5p	hsa-mir-144	0.0944533	0.00124329	0.007470843	-0.534783927	2.86947106
hsa-miR-7704	hsa-mir-7704	0.098740569	0.000391194	0.003134209	3.080257891	6.420471123
hsa-miR-549a-5p	hsa-mir-549a	0.12703665	0.001283592	0.007644756	-1.832700202	1.143983123
hsa-miR-383-5p	hsa-mir-383	0.12736292	0.000375964	0.003048481	-1.680990726	1.291992047
hsa-miR-1290	hsa-mir-1290	0.141731808	7.11024E-05	0.000870035	2.306173955	5.124938484
hsa-miR-146a-5p	hsa-mir-146a	0.164122241	8.91967E-05	0.001034989	8.017925192	10.62508253
hsa-miR-4301	hsa-mir-4301	0.172151709	0.000243564	0.002101522	1.078093292	3.616340884
hsa-miR-1246	hsa-mir-1246	0.180369887	0.000295772	0.002427493	5.845727757	8.316697352
hsa-miR-549a-3p	hsa-mir-549a	0.181420236	0.000929059	0.006315721	0.511363831	2.973956543
hsa-miR-335-3p	hsa-mir-335	0.208248937	8.79385E-08	2.3673E-06	4.370196279	6.633815239
hsa-miR-146b-5p	hsa-mir-146b	0.230667774	1.73312E-07	4.16568E-06	10.30298847	12.4191001
hsa-miR-363-3p	hsa-mir-363	0.236665644	0.01948138	0.069219037	0.631676615	2.710754423
hsa-miR-1291	hsa-mir-1291	0.241621324	8.43371E-05	0.000997889	0.934693326	2.983873637
hsa-miR-146b-3p	hsa-mir-146b	0.242395033	2.72337E-06	4.58207E-05	4.147879725	6.192447686
hsa-miR-4488	hsa-mir-4488	0.259208557	0.005418819	0.026236442	-0.279033218	1.668781534
hsa-miR-139-3p	hsa-mir-139	0.297324694	0.000404396	0.00316463	-0.95546177	0.794427034
hsa-miR-212-5p	hsa-mir-212	0.313169712	0.000757996	0.005259089	3.778248772	5.453232176
hsa-miR-132-3p	hsa-mir-132	0.316338692	0.000211604	0.001898796	6.79999588	8.460453949
hsa-miR-486-5p	hsa-mir-486-1	0.323196414	0.00107596	0.007064415	4.250161968	5.879678872

hsa-miR-147b-3p	hsa-mir-147b	0.323776453	0.015333721	0.058634059	-1.516088562	0.110841463
hsa-miR-132-5p	hsa-mir-132	0.328339084	0.001102142	0.007064415	5.511017176	7.11775878
hsa-miR-486-5p	hsa-mir-486-2	0.335153717	0.001499718	0.008700948	4.34414305	5.92124821
hsa-miR-139-5p	hsa-mir-139	0.338420624	2.56579E-06	4.42763E-05	4.045064022	5.608174626
hsa-miR-3195	hsa-mir-3195	0.340844733	0.005708175	0.027440015	0.695755146	2.248568549
hsa-miR-4485-3p	hsa-mir-4485	0.34166661	0.00169112	0.009645116	0.357246346	1.906585178
hsa-miR-3617-5p	hsa-mir-3617	0.351941014	0.025360348	0.084913006	-1.465412873	0.04118157
hsa-miR-4773	hsa-mir-4773-1	0.368921382	0.01948905	0.069219037	-1.224005019	0.21460967
hsa-miR-4773	hsa-mir-4773-2	0.368921382	0.01948905	0.069219037	-1.224005019	0.21460967
hsa-miR-212-3p	hsa-mir-212	0.372105126	0.007851921	0.034092536	2.689218398	4.115436229
hsa-miR-665	hsa-mir-665	0.374046448	0.001800188	0.010171878	2.330682948	3.749393614
hsa-miR-335-5p	hsa-mir-335	0.388519192	0.002613452	0.014184301	4.092615866	5.456558093
hsa-miR-142-3p	hsa-mir-142	0.396885064	0.003725629	0.018711556	1.615106305	2.948313129
hsa-miR-664a-3p	hsa-mir-664a	0.397639516	8.81796E-07	1.64847E-05	4.274517363	5.604984323
hsa-miR-766-3p	hsa-mir-766	0.40929941	0.008612668	0.036227036	0.004328003	1.293099511
hsa-miR-222-3p	hsa-mir-222	0.415481065	2.31018E-10	1.41341E-08	11.25203572	12.51918109
hsa-miR-12136	hsa-mir-12136	0.415647814	0.007627184	0.033549641	2.172972468	3.439538939
hsa-miR-616-5p	hsa-mir-616	0.417385216	0.001813708	0.010171878	-0.43945564	0.821092954
hsa-miR-150-5p	hsa-mir-150	0.419141797	0.044941847	0.129810571	0.691766196	1.946255895
hsa-miR-4492	hsa-mir-4492	0.4208134	0.039978133	0.122296744	1.037931903	2.286679352
hsa-miR-134-3p	hsa-mir-134	0.428408899	0.010797256	0.043774419	-1.001068253	0.221871395
hsa-miR-369-5p	hsa-mir-369	0.461455176	5.46902E-06	8.55965E-05	4.926438768	6.042176346
hsa-miR-29b-1-5p	hsa-mir-29b-1	0.463082669	0.006211301	0.028992615	0.279594382	1.390252712
hsa-miR-222-5p	hsa-mir-222	0.468744185	0.000502791	0.003678027	1.143791647	2.236918948
hsa-miR-3609	hsa-mir-3609	0.474880696	0.009116671	0.037641223	0.942264671	2.016627654
hsa-miR-708-5p	hsa-mir-708	0.479840012	9.40901E-07	1.71142E-05	8.091990842	9.151365473
hsa-miR-4423-5p	hsa-mir-4423	0.480637505	0.009977026	0.040694173	-0.238847565	0.818131301
hsa-miR-3613-3p	hsa-mir-3613	0.481622158	0.008013696	0.034571905	1.03316868	2.087195006
hsa-miR-9901	hsa-mir-9901	0.483676139	0.013676342	0.053813466	-0.052746373	0.995140356

hsa-miR-1270	hsa-mir-1270	0.484094395	0.049064636	0.136448348	-0.802664346	0.243975357
hsa-miR-485-5p	hsa-mir-485	0.48848404	6.69782E-06	0.000102446	3.944223777	4.977840446
hsa-miR-543	hsa-mir-543	0.490800937	0.000511207	0.003699382	6.35883453	7.385624621
hsa-miR-433-3p	hsa-mir-433	0.495476259	8.45166E-05	0.000997889	4.465946775	5.479058937
hsa-miR-4683	hsa-mir-4683	0.496177535	0.040486678	0.123158872	-0.643902251	0.367169428
hsa-miR-4636	hsa-mir-4636	0.496695563	0.017789782	0.065402103	0.04298746	1.052553694
hsa-miR-668-3p	hsa-mir-668	0.499850223	0.000654257	0.004586613	1.355341335	2.355773566
hsa-miR-99a-5p	hsa-mir-99a	0.504737223	0.017885358	0.065402103	12.12576167	13.11215728
hsa-miR-193a-3p	hsa-mir-193a	0.509466241	0.02976708	0.097618364	1.008571406	1.981512949
hsa-miR-4791	hsa-mir-4791	0.510460299	0.040717015	0.123158872	0.449755715	1.41988505
hsa-miR-486-3p	hsa-mir-486-1	0.511166514	0.0467854	0.132296532	-0.536356028	0.431778737
hsa-miR-486-3p	hsa-mir-486-2	0.514144957	0.047270691	0.132554895	-0.564085628	0.395667301
hsa-miR-377-3p	hsa-mir-377	0.519741772	0.000968757	0.006455182	2.468181859	3.412314939
hsa-miR-125b-1-3p	hsa-mir-125b-1	0.520422023	0.000149667	0.001443371	7.456207435	8.398453516
hsa-miR-134-5p	hsa-mir-134	0.524429661	7.85783E-07	1.51095E-05	7.201491993	8.132670803
hsa-miR-628-5p	hsa-mir-628	0.526771129	0.000428775	0.003279152	2.288669431	3.213421248
hsa-miR-370-3p	hsa-mir-370	0.533308852	0.000140548	0.001391013	8.290801234	9.197758055
hsa-miR-1248	hsa-mir-1248	0.53844619	0.03246167	0.103538881	1.927363459	2.820489379
hsa-miR-495-3p	hsa-mir-495	0.540373259	0.001582077	0.009100321	6.837037497	7.72500931
hsa-miR-127-3p	hsa-mir-127	0.545293155	0.003273208	0.016899588	8.579075087	9.453971136
hsa-miR-4324	hsa-mir-4324	0.545799922	0.024463311	0.082732705	3.603853871	4.477409776
hsa-miR-10395-3p	hsa-mir-10395	0.559232569	0.022620405	0.077609858	2.102513813	2.940993523
hsa-miR-493-5p	hsa-mir-493	0.560082755	0.000115116	0.001156316	9.589132394	10.42542048
hsa-miR-625-5p	hsa-mir-625	0.563182662	0.011039414	0.044488179	1.536047313	2.364372487
hsa-let-7a-2-3p	hsa-let-7a-2	0.565915169	0.002878356	0.015497069	1.70840454	2.529746828
hsa-miR-154-5p	hsa-mir-154	0.572937646	0.004858929	0.023882873	3.882419317	4.685969276
hsa-miR-432-5p	hsa-mir-432	0.580164185	0.0007727	0.005306396	5.144629257	5.930096114
hsa-miR-337-3p	hsa-mir-337	0.581527029	0.001893385	0.010530974	5.536029266	6.318111111
hsa-miR-193a-5p	hsa-mir-193a	0.583736781	0.001230763	0.007462192	5.551760777	6.328370896

hsa-miR-628-3p	hsa-mir-628	0.586106882	0.045673299	0.129942442	0.723532835	1.494297152
hsa-miR-379-3p	hsa-mir-379	0.593147269	0.000939313	0.006321575	4.444476861	5.198014608
hsa-miR-125b-5p	hsa-mir-125b-2	0.594766314	2.23185E-05	0.000300052	12.83563187	13.58523702
hsa-miR-125b-5p	hsa-mir-125b-1	0.59892948	2.27379E-05	0.000300052	12.86776651	13.60730846
hsa-miR-654-5p	hsa-mir-654	0.600401651	0.00117843	0.007411996	5.154806407	5.890806557
hsa-miR-758-3p	hsa-mir-758	0.600816833	0.00053905	0.003859366	4.751932897	5.486935759
hsa-miR-450a-2-3p	hsa-mir-450a-2	0.600873165	0.031314543	0.100835825	0.477271	1.212138603
hsa-miR-137-3p	hsa-mir-137	0.603898175	0.019541779	0.069219037	2.875273723	3.602896506
hsa-miR-382-5p	hsa-mir-382	0.605323449	0.000269159	0.002292964	7.246827158	7.971049013
hsa-miR-411-3p	hsa-mir-411	0.607763794	0.00113772	0.00722345	4.83224833	5.550665693
hsa-miR-221-3p	hsa-mir-221	0.610832637	0.000151363	0.001443371	12.61280915	13.3239601
hsa-miR-1306-5p	hsa-mir-1306	0.611301234	0.030177119	0.098112083	1.010474549	1.720519163
hsa-miR-431-3p	hsa-mir-431	0.611346246	0.015823402	0.059826684	1.573025762	2.282964151
hsa-miR-664a-5p	hsa-mir-664a	0.612327544	0.0179535	0.065402103	2.060252056	2.767876569
hsa-miR-377-5p	hsa-mir-377	0.612625395	0.006957206	0.031424159	3.23598958	3.942912504
hsa-miR-299-3p	hsa-mir-299	0.612995194	0.002978331	0.01578281	3.667565897	4.373618229
hsa-miR-345-5p	hsa-mir-345	0.61509325	0.001456288	0.008587034	5.816548674	6.517671625
hsa-miR-218-5p	hsa-mir-218-1	0.615868233	0.006429002	0.02943346	7.770527425	8.469833805
hsa-miR-218-5p	hsa-mir-218-2	0.616061097	0.006672474	0.030341722	7.745984652	8.444839312
hsa-miR-409-3p	hsa-mir-409	0.620214227	0.005997268	0.028423672	9.072430505	9.76159198
hsa-miR-450a-5p	hsa-mir-450a-1	0.625736524	0.013833179	0.053813466	4.89129602	5.567668797
hsa-miR-450a-5p	hsa-mir-450a-2	0.627337777	0.013779659	0.053813466	4.895909265	5.568594919
hsa-miR-5100	hsa-mir-5100	0.629531764	0.043096549	0.125017145	2.309496767	2.977145689
hsa-miR-1287-5p	hsa-mir-1287	0.631978283	0.033163398	0.104667446	2.172638161	2.834691272
hsa-miR-487b-3p	hsa-mir-487b	0.6462167	0.022717893	0.077609858	5.764493595	6.394403655
hsa-miR-221-5p	hsa-mir-221	0.650335005	0.002969571	0.01578281	8.188057578	8.808802592
hsa-miR-410-3p	hsa-mir-410	0.651883829	0.03038081	0.098299448	4.434194506	5.051507713
hsa-miR-654-3p	hsa-mir-654	0.655406704	0.006289631	0.028992615	8.734971585	9.344509249

Table S7 – Differentially expressed piRNA in osteogenic differentiation.

ID_piRBase	ID_NCBI	Day_7_CRL_ monolayer	Day_7_Osteo	Fold_change ABS	p_value	FDR	accession	length	Expression found in	derive
piR-hsa-27208	piR-34984	5.106956188	7.653179497	5.841032083	0.037010995	0.260251916	DQ596918	27	Testis [1]<br>↑ in bladder cancer [2]<br>↑ in metastatic renal carcinoma [3]<br>↑ in Alzheimer patients [4]	
piR-hsa-26940	piR-34804	3.555533998	6.100915562	5.837625109	0.02289369	0.202838094	DQ596738	32	Testis [1]	
piR-hsa-23617	piR-33466	4.147860165	5.983837447	3.570131668	0.034522942	0.254894386	DQ593354	28	Testis [1]<br>↓ in bladder cancer [2]	
piR-hsa-23533	piR-33382	9.969332094	11.68163307	3.276830336	0.017175749	0.193435737	DQ593270	31	Testis [1]	
piR-hsa-28875	piR-36741	6.668594839	7.695794257	2.038064087	0.005325497	0.181476557	DQ598675	31	Testis [1]	
piR-hsa-12790	piR-32679	6.959103141	7.786688792	1.774712895	0.00199393	0.110413872	DQ582567	29	Testis [1]	
piR-hsa-27007	piR-34871	4.078014085	4.903710837	1.772390811	0.043639363	0.2762892	DQ596805	27	Testis [1]	
piR-hsa-27222	piR-34998	6.943561276	7.661766718	1.64513439	0.009097849	0.193435737	DQ596932	27	Testis [1]<br>Ovary [5]<br>↑ in bladder cancer [2]	
**ID_piRBase**	**ID_NCBI**	**Day_7_CRL_ monolayer**	**Day_7_Osteo**	**Fold_change (ABS)**	**p_value**	**FDR**	**accession**	**length**	**Expression found in**	**derive**
piR-hsa-28392	piR-36243	5.455083753	2.137399963	0.100294626	0.0217925	0.197021991	DQ598177	32	Testis [1]<br>Ovary [5]	
piR-hsa-28404	piR-36255	5.455083753	2.137399963	0.100294626	0.0217925	0.197021991	DQ598189	31	Testis [1]	
piR-hsa-28391	piR-36242	5.410686314	2.137399963	0.103429071	0.01878541	0.193435737	DQ598176	31	Testis [1]<br>Ovary [5]	
piR-hsa-28403	piR-36254	5.410686314	2.137399963	0.103429071	0.01878541	0.193435737	DQ598188	30	Testis [1]	
piR-hsa-28393	piR-36244	5.545837239	2.316250112	0.106609867	0.016749564	0.193435737	DQ598178	32	Testis [1]	
piR-hsa-28407	piR-36258	5.545837239	2.316250112	0.106609867	0.016749564	0.193435737	DQ598192	32	Testis [1]	
piR-hsa-28421	piR-36272	5.545837239	2.316250112	0.106609867	0.016749564	0.193435737	DQ598206	31	Testis [1]	
piR-hsa-4047	piR-41880	2.488058186	-0.703248395	0.109476523	0.014825131	0.193435737	DQ573768	30	Testis [1]	
piR-hsa-28406	piR-36257	5.50418608	2.316250112	0.109732595	0.014422867	0.193435737	DQ598191	30	Testis [1]	
piR-hsa-28405	piR-36256	5.497610173	2.470424343	0.122666582	0.018341239	0.193435737	DQ598190	32	Testis [1]	
piR-hsa-28819	piR-36685	6.667561074	3.868241353	0.143655017	0.01494971	0.193435737	DQ598619	31	Testis [1]	
piR-hsa-21707	piR-58555	1.024973711	-1.550800347	0.167731545	0.047674218	0.289310664	DQ591443	29	Testis [1]	
piR-hsa-14884	piR-51671	0.946982239	-1.471837149	0.18700913	0.045278362	0.278587698	DQ584559	29	Testis [1]	SINE

**piR-hsa-19207**	piR-56037	**2.30984312**	**-0.037073953**	**0.196565621**	**0.001110127**	**0.081964345**	**DQ588925**	**29**	**Testis [2]**<br>**↑ in bladder cancer [3]**	**LTR, SINE**
piR-hsa-5632	piR-43452	1.507169481	-0.703248395	0.216071714	0.025354863	0.216003932	DQ575340	31	Testis [1]<br>↓ in bladder cancer [2]	
piR-hsa-17520	piR-54341	9.101798654	7.029864629	0.237840445	0.004822006	0.178012375	DQ587229	29	Testis [1]	
**piR-hsa-32187**		**7.818001266**	**6.163172968**	**0.31757554**	**0.000133351**	**0.0295373**		**23**	**Epididymis [6]**	
piR-hsa-23679	piR-33543	6.877377516	5.273694448	0.329035906	0.00337271	0.149411061	DQ593431	32	Testis [1]<br>↑ in bladder cancer [2]	
piR-hsa-32260		2.562593118	1.031700949	0.346063294	0.016056485	0.193435737		27	Epididymis [6]	
piR-hsa-6463	piR-44312	7.127865752	5.625286612	0.3529219	0.020150725	0.194060247	DQ576200	28	Testis [1]<br>Ovary [5]	
**piR-hsa-32161**		**8.154212823**	**6.80642216**	**0.392893264**	**0.000257397**	**0.038009022**		**24**	**Epididymis [6]**	
piR-hsa-14647	piR-52729	6.858931272	5.541215135	0.401169509	0.010066325	0.193435737	DQ585617	28	Testis [1]	
piR-hsa-30937	piR-39980	3.076343349	1.851139092	0.427736949	0.043163278	0.2762892	DQ601914	29	Testis [1]	LINE, SINE
piR-hsa-26371	piR-34221	6.204054826	4.994394465	0.432370392	0.033330364	0.254575023	DQ596155	28	Testis [1]	
**piR-hsa-32160**		**4.961010065**	**3.76821745**	**0.437455261**	**0.001095945**	**0.081964345**		**23**	**Epididymis [6]**	
piR-hsa-32163		5.702053557	4.579383727	0.45924317	0.030651942	0.242478756		28	Epididymis [6]	
piR-hsa-3200	piR-41004	9.362257967	8.243234205	0.460405265	0.032921608	0.254575023	DQ572892	29	Testis [1]<br>Epididymis [6]	
piR-hsa-27489	piR-35280	3.964338597	2.862320574	0.465864395	0.03512953	0.255121012	DQ597214	27	Testis [1]	LTR
piR-hsa-11257	piR-49040	7.127867009	6.031032846	0.467541339	0.0138246	0.193435737	DQ580928	31	Testis [1]	LTR
piR-hsa-23220	piR-33054	7.127867009	6.031032846	0.467541339	0.0138246	0.193435737	DQ592942	30	Testis [1]	LTR
piR-hsa-30708	piR-38574	7.127867009	6.031032846	0.467541339	0.0138246	0.193435737	DQ600508	29	Testis [1]	LTR
piR-hsa-23330	piR-33164	3.280879695	2.19994017	0.472720873	0.010744456	0.193435737	DQ593052	28	Testis [1]	
piR-hsa-26441	piR-34291	7.094207824	6.055481866	0.486757138	0.034026442	0.254894386	DQ596225	30	Testis [1]	
piR-hsa-12423	piR-32311	6.745064259	5.858856822	0.541034525	0.029217255	0.239689701	DQ582199	32	Testis [1]<br>Ovary [5]	
piR-hsa-23621	piR-33470	9.128810358	8.325273606	0.572942891	0.001826134	0.110413872	DQ593358	26	Testis [1]<br>↑ in bladder cancer [2]	
piR-hsa-12485	piR-32374	8.567901944	7.774924796	0.577151851	0.010457299	0.193435737	DQ582262	31	Testis [1]	
piR-hsa-27400	piR-35176	8.580468195	7.787918063	0.577322704	0.010947381	0.193435737	DQ597110	32	Testis [1]	
piR-hsa-27138	piR-35467	7.251664759	6.519044792	0.60181002	0.018728714	0.193435737	DQ597401	30	Testis [1]	
piR-hsa-1100	piR-30961	8.663276725	7.933734331	0.603095178	0.009638264	0.193435737	DQ570849	31	Testis [1]	
piR-hsa-1823	piR-31612	11.93771801	11.22474979	0.610063695	0.019976465	0.194060247	DQ571500	30	Testis [1]	

**piR-hsa-28223**	**piR-36074**	**12.33730461**	**11.62546881**	**0.610542741**	**5.1333E-05**	**0.022740516**	**DQ598008**	**30**	**Testis [1]**	
piR-hsa-23327	piR-33161	7.653871483	6.994534552	0.633169237	0.024131215	0.209610357	DQ593049	29	Testis [1]	
piR-hsa-1834	piR-31623	7.581229699	6.924376726	0.634260335	0.030018408	0.241784631	DQ571511	30	Testis [1]	
piR-hsa-426	piR-30229	8.804519376	8.154820671	0.637413418	0.012350521	0.193435737	DQ570117	31	Testis [1]<br>Ovary [5]	
piR-hsa-28487	piR-36338	7.15679813	6.534192305	0.649496733	0.004212363	0.169643355	DQ598272	30	Testis [1]<br>Ovary [5]	
piR-hsa-27139	piR-35468	8.303537463	7.694205522	0.65550017	0.01531931	0.193435737	DQ597402	31	Testis [1]	

Table S8 – Differentially expressed piRNA in chondrogenic differentiation.

ID_piRBase	ID_NCBI	Day_7_CRL_ pellet	Day_7_Chondro	fold_change	p_value	FDR	accession	length	pubmed	derive
**piR-hsa-28397**	**piR-36248**	**-1.072260336**	**3.386386057**	**21.98802916**	**0.002945706**	**0.035268858**	**DQ598182**	**29**	**Testis [1]**	
piR-hsa-27134	piR-35463	-0.285512967	3.278387371	11.82608247	0.014896466	0.085533989	DQ597397	31	Testis [1]	
piR-hsa-11257	piR-49040	2.398606667	5.388198397	7.942491994	0.006396667	0.055156292	DQ580928	31	Testis [1]	LTR
piR-hsa-23220	piR-33054	2.398606667	5.388198397	7.942491994	0.006396667	0.055156292	DQ592942	30	Testis [1]	LTR
piR-hsa-30708	piR-38574	2.398606667	5.388198397	7.942491994	0.006396667	0.055156292	DQ600508	29	Testis [1]	LTR
**piR-hsa-28398**	**piR-36249**	**1.826697734**	**4.556179972**	**6.632175749**	**6.16764E-05**	**0.005464528**	**DQ598183**	**30**	**Testis [1]**<br>**↓ in breast cancer [7]**	
piR-hsa-20757	piR-57660	7.034113323	9.707358018	6.378621595	0.006194159	0.055156292	DQ590548	27	Testis [1]	
piR-hsa-26491	piR-34341	0.5405795	3.089531464	5.852090027	0.009602785	0.064455058	DQ596275	30	Testis [1]	
piR-hsa-27222	piR-34998	3.725532079	6.230312886	5.675631043	0.012834728	0.081987921	DQ596932	27	Testis [1]<br>Ovary [5]<br>↑ in bladder cancer [2]	
**piR-hsa-456**	**piR-30259**	**3.0389135**	**5.477064408**	**5.419466765**	**0.003534522**	**0.037280791**	**DQ570147**	**28**	**Testis [1]**	
piR-hsa-5952	piR-43786	2.245885692	4.670868708	5.370226791	0.022633895	0.097109748	DQ575674	31	Testis [1]	
piR-hsa-28185	piR-36036	2.124933427	4.482852524	5.126304217	0.020428442	0.095043373	DQ597970	26	Testis [1]	
piR-hsa-28183	piR-36034	2.124933427	4.45186935	5.017385966	0.022810142	0.097109748	DQ597968	26	Testis [1]	
piR-hsa-28184	piR-36035	2.124933427	4.45186935	5.017385966	0.022810142	0.097109748	DQ597969	31	Testis [1]<br>Ovary [5]	
piR-hsa-28384	piR-36235	2.247814147	4.556070905	4.952842549	0.02263758	0.097109748	DQ598169	31	Testis [1]	
piR-hsa-28186	piR-36037	2.311863363	4.586645288	4.83924481	0.034713526	0.121087339	DQ597971	26	Testis [1]	

**piR-hsa-1245**	**piR-31106**	**4.950746977**	**7.049734965**	**4.284087623**	**0.000187004**	**0.010141521**	**DQ570994**	**26**	**Testis [1]**<br>**Ovary [5]**<br>**Epididymis [6]**<br>**↑ in breast cancer[7]**	
piR-hsa-23087	piR-59892	1.119902383	3.212915568	4.266382115	0.014279179	0.084342353	DQ592780	30	Testis [1]	
piR-hsa-1556	piR-31331	0.125925003	2.208176504	4.234675736	0.034517663	0.121087339	DQ571219	28	Testis [1]	
**piR-hsa-28400**	**piR-36251**	**2.584685343**	**4.601929398**	**4.048097547**	**8.78314E-06**	**0.001945466**	**DQ598185**	**30**	**Testis [1]**	
piR-hsa-5302	piR-43108	0.399492555	2.372624208	3.926194523	0.019134699	0.094301156	DQ574996	30	Testis [1]	
piR-hsa-29714	piR-37523	1.555895889	3.462476092	3.749193282	0.031878659	0.115756116	DQ599457	27	Testis [1]<br>↑ in bladder cancer [2]	
piR-hsa-29715	piR-37524	1.555895889	3.462476092	3.749193282	0.031878659	0.115756116	DQ599458	29	Testis [1]<br>↑ in bladder cancer [2]	
piR-hsa-29716	piR-37525	1.555895889	3.462476092	3.749193282	0.031878659	0.115756116	DQ599459	30	Testis [1]<br>↑ in bladder cancer [2]	
piR-hsa-26593	piR-34443	4.991868614	6.734026677	3.3453521	0.023016983	0.097109748	DQ596377	32	Testis [1]	
piR-hsa-26594	piR-34444	4.991868614	6.72771075	3.330738604	0.025282382	0.099115889	DQ596378	32	Testis [1]<br>Ovary [5]	
**piR-hsa-1344**	**piR-31686**	**5.905414756**	**7.635284839**	**3.31697947**	**0.001080885**	**0.02389523**	**DQ571574**	**32**	**Testis [1]**	
**piR-hsa-23230**	**piR-33064**	**5.905414756**	**7.635284839**	**3.31697947**	**0.001080885**	**0.02389523**	**DQ592952**	**30**	**Testis [1]**	
piR-hsa-26589	piR-34439	4.991868614	6.720169179	3.313372867	0.024667432	0.097568504	DQ596373	30	Testis [1]<br>Ovary [5]	
piR-hsa-26591	piR-34441	4.991868614	6.720169179	3.313372867	0.024667432	0.097568504	DQ596375	30	Testis [1]<br>Ovary [5]	
piR-hsa-26592	piR-34442	4.991868614	6.720169179	3.313372867	0.024667432	0.097568504	DQ596376	31	Testis [1]<br>Ovary [5]	
piR-hsa-26590	piR-34440	5.033450183	6.725980908	3.232231935	0.024663884	0.097568504	DQ596374	31	Testis [1]<br>Ovary [5]	
**piR-hsa-27138**	**piR-35467**	**5.038594157**	**6.700776918**	**3.164950117**	**9.77961E-05**	**0.007220615**	**DQ597401**	**30**	**Testis [1]**	
piR-hsa-10934	piR-48820	0.89663331	2.476161785	2.988721514	0.032741736	0.117923488	DQ580708	30	Testis [1]	SINE
**piR-hsa-27139**	**piR-35468**	**6.765267241**	**8.243157219**	**2.785410534**	**4.79393E-05**	**0.005309279**	**DQ597402**	**31**	**Testis [1]**	
piR-hsa-12487	piR-32376	2.500427299	3.788698509	2.442352116	0.009112105	0.06337349	DQ582264	31	Testis [1]	
piR-hsa-31237	piR-39017	2.076433351	3.329776574	2.383932228	0.006021532	0.055156292	DQ600951	31	Testis [1]<br>↑ in bladder cancer [2]	
**piR-hsa-27140**	**piR-35469**	**7.824207534**	**9.053573367**	**2.344639036**	**3.83459E-05**	**0.005309279**	**DQ597403**	**32**	**Testis [1]**	
**piR-hsa-15603**	**piR-52404**	**7.947167933**	**9.164400461**	**2.325002919**	**0.002293052**	**0.032979166**	**DQ585292**	**28**	**Testis [1]**	
**piR-hsa-23621**	**piR-33470**	**6.943279294**	**8.144176432**	**2.298825788**	**0.001132731**	**0.02389523**	**DQ593358**	**26**	**Testis [1]**<br>**↑ in bladder cancer [2]**	
**piR-hsa-3645**	**piR-41464**	**10.86087377**	**12.02338586**	**2.238468609**	**0.002531132**	**0.032979166**	**DQ573352**	**26**	**Testis [1]**	**LINE**

**piR-hsa-28382**	**piR-36233**	**11.67700007**	**12.80046652**	**2.178698331**	**0.001958186**	**0.031790858**	**DQ598167**	**26**	**Testis [1]**	
piR-hsa-5936	piR-43770	7.88640433	8.999973716	2.163803343	0.03791441	0.126286342	DQ575658	26	Testis [1]<br>Ovary [5]	
**piR-hsa-1043**	**piR-30890**	**7.031848679**	**8.144065096**	**2.161775064**	**0.006541799**	**0.055156292**	**DQ570778**	**31**	**Testis [1]**	
**piR-hsa-1731**	**piR-31520**	**8.807654062**	**9.904849624**	**2.139384157**	**0.004019052**	**0.040464549**	**DQ571408**	**30**	**Testis [1]**	
**piR-hsa-19620**	**piR-56450**	**10.50492066**	**11.59361352**	**2.126812521**	**0.001949619**	**0.031790858**	**DQ589338**	**29**	**Testis [1]**	
**piR-hsa-12488**	**piR-32377**	**2.766513008**	**3.83745945**	**2.100811098**	**0.020143262**	**0.095043373**	**DQ582265**	**32**	**Testis [1]**<br>**Ovary [5]**	
**piR-hsa-2153**	**piR-31985**	**5.581877926**	**6.626124842**	**2.062289567**	**0.028937732**	**0.108639112**	**DQ571873**	**26**	**Testis [1]**<br>**Ovary [5]**	
**piR-hsa-28401**	**piR-36252**	**3.470843787**	**4.488092318**	**2.024055052**	**0.00908264**	**0.06337349**	**DQ598186**	**31**	**Testis [1]**<br>**Ovary [5]**	
**piR-hsa-14870**	**piR-51657**	**9.460675832**	**10.45931285**	**1.998111402**	**0.007673052**	**0.058167632**	**DQ584545**	**28**	**Testis [1]**	
**piR-hsa-1219**	**piR-31080**	**8.420403869**	**9.407947044**	**1.982805513**	**0.002664524**	**0.033116891**	**DQ570968**	**27**	**Testis [1]**	
**piR-hsa-28525**	**piR-36376**	**11.73763451**	**12.7212097**	**1.977359503**	**0.009798074**	**0.064784282**	**DQ598310**	**32**	**Testis [1]**	
**piR-hsa-26399**	**piR-34249**	**8.204153399**	**9.169566944**	**1.952623156**	**0.039206255**	**0.128654599**	**DQ596183**	**28**	**Testis [1]**	
piR-hsa-28212	piR-36063	12.84058645	13.79506601	1.937880426	0.005270221	0.05075452	DQ597997	30	Testis [1]	
piR-hsa-17520	piR-54341	7.047310028	7.936546288	1.852195342	0.045240373	0.142138193	DQ587229	29	Testis [1]	
**piR-hsa-12719**	**piR-32608**	**10.03834842**	**10.88918593**	**1.803547607**	**0.003208547**	**0.036445803**	**DQ582496**	**26**	**Testis [1]**	
piR-hsa-27619	piR-35410	6.045751707	6.865423893	1.765004896	0.007746931	0.058167632	DQ597344	29	Testis [1]	
**piR-hsa-27623**	**piR-35414**	**9.99628936**	**10.78149469**	**1.723337572**	**0.003344104**	**0.037035949**	**DQ597348**	**32**	**Testis [1]**	
**piR-hsa-25786**	**piR-61651**	**11.09281489**	**11.86406648**	**1.706749808**	**0.00249822**	**0.032979166**	**DQ595539**	**27**	**Testis [1]**	**LINE**
piR-hsa-29218	piR-36984	8.913090635	9.682626042	1.704720721	0.008692135	0.06337349	DQ598918	31	Testis [1]<br>↑ in bladder cancer [2]	
**piR-hsa-2117**	**piR-31935**	**12.23166071**	**12.99308202**	**1.695159843**	**0.0031098**	**0.036253721**	**DQ571823**	**31**	**Testis [1]**	**LINE**
**piR-hsa-1191**	**piR-31052**	**11.60305151**	**12.35044072**	**1.678752103**	**0.00174222**	**0.03092551**	**DQ570940**	**29**	**Testis [1]**	
**piR-hsa-23533**	**piR-33382**	**14.29567449**	**15.03724253**	**1.671992105**	**0.001745232**	**0.03092551**	**DQ593270**	**31**	**Testis [1]**	
**piR-hsa-28478**	**piR-36329**	**13.25172422**	**13.99256429**	**1.671148657**	**0.004165421**	**0.041006254**	**DQ598263**	**29**	**Testis [1]**	
**piR-hsa-12525**	**piR-32414**	**11.0422514**	**11.78016641**	**1.667763833**	**0.002691215**	**0.033116891**	**DQ582302**	**28**	**Testis [1]**	
**piR-hsa-20613**	**piR-57516**	**11.39223795**	**12.11670931**	**1.652295085**	**0.002009354**	**0.031790858**	**DQ590404**	**31**	**Testis [1]**	**LINE**
**piR-hsa-182**	**piR-30438**	**11.83768939**	**12.55243854**	**1.64119783**	**0.002398855**	**0.032979166**	**DQ570326**	**29**	**Testis [1]**	
**piR-hsa-5770**	**piR-43604**	**4.459593517**	**5.130613193**	**1.592197912**	**0.000550329**	**0.018753519**	**DQ575492**	**29**	**Testis [1]**	
**piR-hsa-11080**	**piR-48966**	**9.092410248**	**9.74392613**	**1.570817833**	**0.000454244**	**0.016769188**	**DQ580854**	**29**	**Testis [1]**	

piR-hsa-28646	piR-36511	8.7906587	9.440217941	1.568688871	0.015060161	0.085533989	DQ598445	28	Testis [1]<br>Ovary [5]	
piR-hsa-1242	piR-31103	6.719082386	7.321606855	1.518371133	0.021025396	0.095043373	DQ570991	31	Testis [1]<br>Ovary [5]	
piR-hsa-27620	piR-35411	6.719082386	7.321606855	1.518371133	0.021025396	0.095043373	DQ597345	30	Testis [1]	
piR-hsa-23588	piR-33437	6.755867961	7.356566978	1.516451142	0.016933774	0.092613111	DQ593325	28	Testis [1]	
**ID_piRBase**	**ID_NCBI**	**Day_7_CRL_ pellet**	**Day_7_Chondro**	**fold_change**	**p_value**	**FDR**	**accession**	**length**	**pubmed**	**derive**
piR-hsa-13940	piR-50786	3.216310727	-0.894389382	0.057883657	0.01340623	0.082485556	DQ583674	30	Testis [1]	
piR-hsa-32186		6.01694676	2.680689129	0.099011669	0.0437138	0.138322953		23	Epididymis [6]	
piR-hsa-8757	piR-46628	1.848742258	-0.894389382	0.149360272	0.024105774	0.097568504	DQ578516	29	Testis [1]<br>Ovary [5]	
**piR-hsa-28190**	**piR-36041**	**11.2584113**	**9.104318336**	**0.224674303**	**0.00105046**	**0.02389523**	**DQ597975**	**30**	**Testis [1]**<br>**Ovary [5]**<br>**↑ in breast cancer [8]**	
piR-hsa-1359	piR-31701	7.080870204	4.933446732	0.225715364	0.036935954	0.124905555	DQ571589	32	Testis [1]	
piR-hsa-28593	piR-36444	8.133108839	5.991579712	0.226639444	0.041137177	0.133020215	DQ598378	32	Testis [1]	
**piR-hsa-7193**	**piR-44984**	**10.80410993**	**8.736909219**	**0.238622054**	**0.001524756**	**0.029368135**	**DQ576872**	**31**	**Testis [1]**<br>**↑ in breast cancer [8]**	
**piR-hsa-26508**	**piR-34358**	**6.587909773**	**4.694108537**	**0.269097103**	**2.81426E-07**	**0.000124672**	**DQ596292**	**28**	**Testis [1]**	
piR-hsa-32187		12.01552646	10.18723397	0.281597712	0.02150971	0.09625052		23	Epididymis [6]	
**piR-hsa-12790**	**piR-32679**	**11.20641275**	**9.399715225**	**0.285844508**	**0.003506128**	**0.037280791**	**DQ582567**	**29**	**Testis [1]**	
piR-hsa-32179		9.981110216	8.181429801	0.287238211	0.015334137	0.085987628		24	Epididymis [6]	
piR-hsa-32160		9.789522929	8.008461982	0.290969341	0.022984046	0.097109748		23	Epididymis [6]	
piR-hsa-24684	piR-60577	8.212238426	6.456969538	0.296217979	0.023666781	0.097568504	DQ594465	32	Testis [1]	
piR-hsa-32235		9.928355408	8.179627313	0.297564001	0.018614885	0.094301156		24	Epididymis [6]	
piR-hsa-32162		12.53831638	10.82274148	0.304481209	0.015542891	0.086068759		25	Epididymis [6]	
piR-hsa-32238		9.939574104	8.23246718	0.306273633	0.019146027	0.094301156		25	Epididymis [6]	
piR-hsa-32161		12.34890574	10.65239447	0.308531292	0.020498686	0.095043373		24	Epididymis [6]	
piR-hsa-28319	piR-36170	8.106114852	6.41583136	0.30986603	0.012556167	0.081799732	DQ598104	30	Testis [1]<br>Ovary [5]	
piR-hsa-32167		10.24249255	8.554278073	0.310310736	0.006847398	0.055156292		25	Epididymis [6]	
piR-hsa-24683	piR-60576	7.373944747	5.691091761	0.311466091	0.019583987	0.094301156	DQ594464	30	Testis [1]	
piR-hsa-32195		12.49271127	10.83003513	0.315852711	0.017860551	0.094301156		25	Epididymis [6]	

piR-hsa-25779	piR-61644	5.275947937	3.645290352	0.322940977	0.019218662	0.094301156	DQ595532	27	Testis [1]	
piR-hsa-23662	piR-33526	5.885813116	4.279058384	0.328336096	0.033876129	0.120057002	DQ593414	27	Testis [1]	
piR-hsa-2106	piR-31924	11.23656655	9.63332746	0.329137179	0.033648053	0.120057002	DQ571812	29	Testis [1]	
piR-hsa-28318	piR-36169	6.69810998	5.10777096	0.332093405	0.009119301	0.06337349	DQ598103	29	Testis [1]	
piR-hsa-23210	piR-33044	12.51875559	10.9326909	0.333078771	0.018727361	0.094301156	DQ592932	26	Testis [1]<br>Epididymis [6]	
piR-hsa-32159		12.91713209	11.34157871	0.335514407	0.006879744	0.055156292		26	Epididymis [6]	
piR-hsa-23663	piR-33527	6.058341439	4.53848123	0.348719704	0.029247994	0.108881187	DQ593415	28	Testis [1]	
**piR-hsa-26872**	**piR-34736**	**8.81369211**	**7.313800182**	**0.353579876**	**0.001512532**	**0.029368135**	**DQ596670**	**27**	**Testis [1]<br>↓ in breast cancer [7]**	
**piR-hsa-23231**	**piR-33065**	**10.75456517**	**9.277385575**	**0.359190326**	**0.000631684**	**0.019988295**	**DQ592953**	**30**	**Testis [1]<br>Ovary [5]**	
piR-hsa-2107	piR-31925	11.80261201	10.47078523	0.397264895	0.041808795	0.134212291	DQ571813	31	Testis [1]<br>Sperm [9]	
**piR-hsa-24672**	**piR-60565**	**14.43347228**	**13.18808576**	**0.421794879**	**0.000140409**	**0.008885885**	**DQ594453**	**31**	**Testis [1]<br>Ovary [5]**	
piR-hsa-28848	piR-36714	7.177693275	5.939115093	0.423790108	0.047981885	0.148245513	DQ598648	30	Testis [1]	
piR-hsa-29114	piR-37213	3.666057247	2.454288917	0.431739103	0.013369463	0.082485556	DQ599147	31	Testis [1]<br>Ovary [5]	
piR-hsa-27065	piR-34929	8.315051091	7.105150141	0.432298295	0.019563726	0.094301156	DQ596863	31	Testis [1]	
**piR-hsa-5937**	**piR-43771**	**15.5984394**	**14.39450826**	**0.434090834**	**0.000686611**	**0.020277916**	**DQ575659**	**31**	**Testis [1]<br>Ovary [5]**	
**piR-hsa-32182**		**11.11332294**	**9.95736207**	**0.448767197**	**0.000206035**	**0.010141521**		**26**	**Ovary [5]<br>Epididymis [6]**	
**piR-hsa-20266**	**piR-57125**	**8.311148561**	**7.16545419**	**0.451972101**	**0.001039292**	**0.02389523**	**DQ590013**	**27**	**Testis [1]<br>↓ in renal carcinoma [10]**	
piR-hsa-14625	piR-52475	2.627183826	1.501360198	0.458240339	0.012955202	0.081987921	DQ585363	30	Testis [1]	SINE
**piR-hsa-26570**	**piR-34420**	**7.99816947**	**6.900086977**	**0.467136962**	**0.002216204**	**0.032979166**	**DQ596354**	**31**	**Testis [1]<br>↓ in bladder cancer [2]**	
piR-hsa-23209	piR-33043	14.00550255	12.92179947	0.47181622	0.000424358	0.016769188	DQ592931	27	Testis [1]<br>Ovary [5]<br>Epididymis [6]<br>↑ in bladder cancer [2]	
piR-hsa-18287	piR-55152	4.61213852	3.538153314	0.475005062	0.013713215	0.083218549	DQ588040	32	Testis [1]	
piR-hsa-5938	piR-43772	15.85413439	14.78391684	0.476247178	0.000334894	0.014835788	DQ575660	32	Testis [1]<br>Ovary [5]	
piR-hsa-18286	piR-55151	4.446356409	3.441717211	0.498394759	0.019219961	0.094301156	DQ588039	31	Testis [1]	
piR-hsa-26526	piR-34376	4.892336963	3.888063626	0.498521165	0.026014639	0.101091973	DQ596310	30	Testis [1]<br>↓ in bladder cancer [2]	LINE
piR-hsa-28849	piR-36715	7.619051383	6.67290302	0.519016258	0.020983526	0.095043373	DQ598649	31	Testis [1]	

piR-hsa-25981	piR-61861	2.427875894	1.507664114	0.528431444	0.03723116	0.12495003	DQ595749	29	Testis [1]	SINE
piR-hsa-23387	piR-33221	3.593729534	2.677825293	0.53001157	0.018216555	0.094301156	DQ593109	31	Testis [1]	LINE, LTR, SINE
piR-hsa-28850	piR-36716	7.842502502	6.933602137	0.532590882	0.01417269	0.084342353	DQ598650	32	Testis [1]	
piR-hsa-1580	piR-31355	7.335933361	6.481661881	0.553144578	0.027583296	0.104439317	DQ571243	29	Testis [1]	
piR-hsa-28189	piR-36040	7.335933361	6.481661881	0.553144578	0.027583296	0.104439317	DQ597974	28	Testis [1]<br>Ovary [5]	
piR-hsa-18071	piR-54907	9.591267377	8.809401983	0.581614284	0.009379157	0.063922564	DQ587795	29	Testis [1]<br>Ovary [5]	
piR-hsa-28188	piR-36039	7.148030244	6.426574356	0.606485102	0.046031974	0.143606791	DQ597973	27	Testis [1]<br>Ovary [5]	
piR-hsa-28487	piR-36338	6.485291881	5.78111644	0.613793197	0.006684231	0.055156292	DQ598272	30	Testis [1]<br>Ovary [5]	
piR-hsa-26527	piR-34377	9.196251298	8.543494471	0.636063705	0.023364228	0.097568504	DQ596311	31	Testis [1]<br>↓ in bladder cancer [2]<br>↓ in breast cancer [7]	LINE
piR-hsa-27493	piR-35284	12.9158631	12.28489601	0.645743403	0.042446913	0.135280448	DQ597218	30	Testis [1]<br>Ovary [5]<br>↑ in breast cancer [8]	
**piR-hsa-28527**	**piR-36378**	**8.07962149**	**7.477399354**	**0.65873854**	**0.002409832**	**0.032979166**	**DQ598312**	**29**	**Testis [1]<br>↑ in breast cancer [8]**	
**piR-hsa-28877**	**piR-36743**	**13.83424207**	**13.24574165**	**0.665033801**	**0.000834765**	**0.023112568**	**DQ598677**	**32**	**Testis [1]<br>↑ in breast cancer [7]**	

Table references:

1. Girard, A., et al., *A germline-specific class of small RNAs binds mammalian Piwi proteins.* Nature, 2006. **442**: p. 199.
2. Chu, H., et al., *Identification of novel piRNAs in bladder cancer.* Cancer Letters, 2015. **356**(2, Part B): p. 561-567.
3. Li, Y., et al., *Piwi-Interacting RNAs (piRNAs) Are Dysregulated in Renal Cell Carcinoma and Associated with Tumor Metastasis and Cancer-Specific Survival.* Molecular medicine (Cambridge, Mass.), 2015. **21**(1): p. 381-388.
4. Roy, J., et al., *Small RNA sequencing revealed dysregulated piRNAs in Alzheimer's disease and their probable role in pathogenesis.* Molecular BioSystems, 2017. **13**(3): p. 565-576.
5. Roovers, Elke F., et al., *Piwi Proteins and piRNAs in Mammalian Oocytes and Early Embryos.* Cell Reports, 2015. **10**(12): p. 2069-2082.
6. Li, Y., et al., *Deep sequencing analysis of small non-coding RNAs reveals the diversity of microRNAs and piRNAs in the human epididymis.* Gene, 2012. **497**(2): p. 330-335.
7. Hashim, A., et al., *RNA sequencing identifies specific PIWI-interacting small non-coding RNA expression patterns in breast cancer.* Oncotarget, 2014. **5**(20): p. 9901-9910.
8. Huang, G., et al., *Altered expression of piRNAs and their relation with clinicopathologic features of breast cancer.* Clinical and Translational Oncology, 2013. **15**(7): p. 563-568.
9. Krawetz, S.A., et al., *A survey of small RNAs in human sperm.* Hum Reprod, 2011. **26**(12): p. 3401-12.
10. Busch, J., et al., *Piwi-interacting RNAs as novel prognostic markers in clear cell renal cell carcinomas.* Journal of Experimental & Clinical Cancer Research, 2015. **34**(1): p. 61.
